# Causal relationship between gut microbiota and chronic renal failure: a two-sample Mendelian randomization study

**DOI:** 10.3389/fmicb.2024.1356478

**Published:** 2024-04-03

**Authors:** Xingzheng Liu, Jinying Mo, Xuerui Yang, Ling Peng, Youjia Zeng, Yihou Zheng, Gaofeng Song

**Affiliations:** ^1^The Fourth Clinical Medical College of Guangzhou University of Chinese Medicine, Shenzhen, China; ^2^Department of Nephrology, Shenzhen Traditional Chinese Medicine Hospital, Shenzhen, China

**Keywords:** chronic renal failure, gut microbiota, gut-kidney axis, Mendelian randomization, causal inference

## Abstract

**Background:**

Observational studies and some experimental investigations have indicated that gut microbiota are closely associated with the incidence and progression of chronic renal failure. However, the causal relationship between gut microbiota and chronic renal failure remains unclear. The present study employs a two-sample Mendelian randomization approach to infer the causal relationship between gut microbiota and chronic renal failure at the genetic level. This research aims to determine whether there is a causal effect of gut microbiota on the risk of chronic renal failure, aiming to provide new evidence to support targeted gut therapy for the treatment of chronic renal failure.

**Methods:**

Employing genome-wide association study (GWAS) data from the public MiBioGen and IEU OpenGWAS platform, a two-sample Mendelian randomization analysis was conducted. The causal relationship between gut microbiota and chronic renal failure was inferred using five different methods: Inverse Variance Weighted, MR-Egger, Weighted Median, Simple Mode, and Weighted Mode. The study incorporated sensitivity analyses that encompassed evaluations for pleiotropy and heterogeneity. Subsequently, the results of the Mendelian randomization analysis underwent a stringent correction for multiple testing, employing the False Discovery Rate method to enhance the validity of our findings.

**Results:**

According to the results from the Inverse Variance Weighted method, seven bacterial genera show a significant association with the outcome variable chronic renal failure. Of these, Ruminococcus (gauvreauii group) (OR = 0.82, 95% CI = 0.71–0.94, *p* = 0.004) may act as a protective factor against chronic renal failure, while the genera Escherichia-Shigella (OR = 1.22, 95% CI = 1.08–1.38, *p* = 0.001), Lactococcus (OR = 1.1, 95% CI = 1.02–1.19, *p* = 0.013), Odoribacter (OR = 1.23, 95% CI = 1.03–1.49, *p* = 0.026), Enterorhabdus (OR = 1.14, 95% CI = 1.00–1.29, *p* = 0.047), Eubacterium (eligens group) (OR = 1.18, 95% CI = 1.02–1.37, *p* = 0.024), and Howardella (OR = 1.18, 95% CI = 1.09–1.28, *p* < 0.001) may be risk factors for chronic renal failure. However, after correction for multiple comparisons using False Discovery Rate, only the associations with Escherichia-Shigella and Howardella remain significant, indicating that the other genera have suggestive associations. Sensitivity analyses did not reveal any pleiotropy or heterogeneity.

**Conclusion:**

Our two-sample Mendelian randomization study suggests that the genera Escherichia-Shigella and Howardella are risk factors for chronic renal failure, and they may serve as potential targets for future therapeutic interventions. However, the exact mechanisms of action are not yet clear, necessitating further research to elucidate their precise roles fully.

## 1 Introduction

Chronic renal failure (CRF) refers to a state in which the kidneys gradually lose function and can no longer maintain the normal internal environment of the body. It is the consequence of the progression of various chronic kidney diseases (CKD), with some patients having to wait for renal replacement therapy. The pathogenesis of CRF is relatively complex, as CKD can typically cause chronic inflammation of the renal tubules and interstitium. This inflammatory response leads to kidney damage and promotes fibrosis, ultimately resulting in the deterioration of renal function (Peppa et al., 2004; Fang et al., 2023). Patients with CRF are also affected by oxidative stress; an excess generation of reactive oxygen species causes an imbalance between oxidation and antioxidation in the body, ultimately leading to pathologies such as lipid peroxidation of cell membranes, renal mitochondrial homeostasis disturbance, and tissue damage (Ho and Shirakawa, 2022; Piko et al., 2023). Additionally, the onset of CRF involves genetic susceptibility and epigenetics (Smyth et al., 2014). The 2023 ISN Global Kidney Health Atlas (ISN-GKHA) released by the International Society of Nephrology (ISN) shows that the global median prevalence of CKD is 9.5%, and the global median mortality rate is 2.4%. Due to the high cost of renal disease treatment and its significant impacts on health, the global burden of renal failure remains substantial. However, there are currently no methods to fully control the progression of CRF. For some renal failure patients, particularly those entering end-stage renal disease (ESRD), treatment still relies mainly on dialysis. Given the expense and complexity of CRF treatment, actively exploring new potential intervention measures to delay the progression of the disease is particularly important.

The gut microbiome is not only crucial for intestinal health but also affects the function of numerous other organs in the body. An increasing body of evidence supports the association between the human gut microbiome and extraintestinal organ function, including a close correlation between the gut microbiome and CRF (Lozupone et al., 2012). Studies have indicated that patients with CKD exhibit dysbiosis of the gut microbiota, with an increase in pathogenic bacteria, impaired gut barrier function, increased intestinal permeability, and generation of uremic toxins such as indoles (e.g., indoxyl sulfate), phenols (e.g., p-cresyl sulfate), and amines (e.g., trimethylamine-N-oxide) through the fermentation of undigested products in the colon by the gut microbiota. These uremic toxins and the impairment of gut barrier function are closely related to inflammatory responses and oxidative stress, which further lead to the progression of the disease and the occurrence of related complications (Mafra et al., 2014; Cigarran Guldris et al., 2017). The gut and kidney can be interconnected through a Metabolism-dependent pathway and an Immune pathway, forming the “gut-kidney axis” (Yang et al., 2018). With the proposal of the “gut-kidney axis” concept, targeting the gut for treatment may become an emerging potential therapeutic strategy for CRF (Martínez-Hernández et al., 2023). While some observational studies and experiments suggest that the gut microbiota plays a significant role in the development and progression of CRF (Tian et al., 2022), the specific causal relationship between the gut microbiota and CRF remains unclear, and there is inconsistency in the findings of gut microbiota studies among dialysis patients, nondialysis patients, and those undergoing hemodialysis or peritoneal dialysis (Stadlbauer et al., 2017; Hu J. et al., 2020). Therefore, in this study, a two-sample Mendelian randomization approach was employed from a genetic perspective to clarify the causal relationship between gut microbiota and CRF, providing new evidence to support targeted gut therapy for CRF.

Mendelian randomization (MR) studies are commonly used to assess the causal relationships between exposures or risk factors and outcomes (Sekula et al., 2016). Observational studies are often subject to confounding by extraneous factors, whereas MR uses genetic variants as instrumental variables to infer causal effects of exposures or risk factors on outcomes. According to Mendel’s laws, alleles of genetic variants are randomly allocated and less likely to be confounded by other factors; thus, MR serves as an analytical method that sits between randomized controlled trials and observational studies (Ference et al., 2021). It can circumvent the confounding inherent to observational studies and overcome issues such as the costly nature and practical challenges associated with randomized trials. To date, research integrating gut microbiota with MR has been widely applied, including in areas such as gut microbiota and cancer (Long et al., 2023), gut microbiota and autoimmune diseases (Xu et al., 2021), and gut microbiota and mental disorders (Ni et al., 2021). This study employs MR to infer the causal relationship between the gut microbiota and CRF.

## 2 Materials and methods

### 2.1 Study design

**Figure 1:**
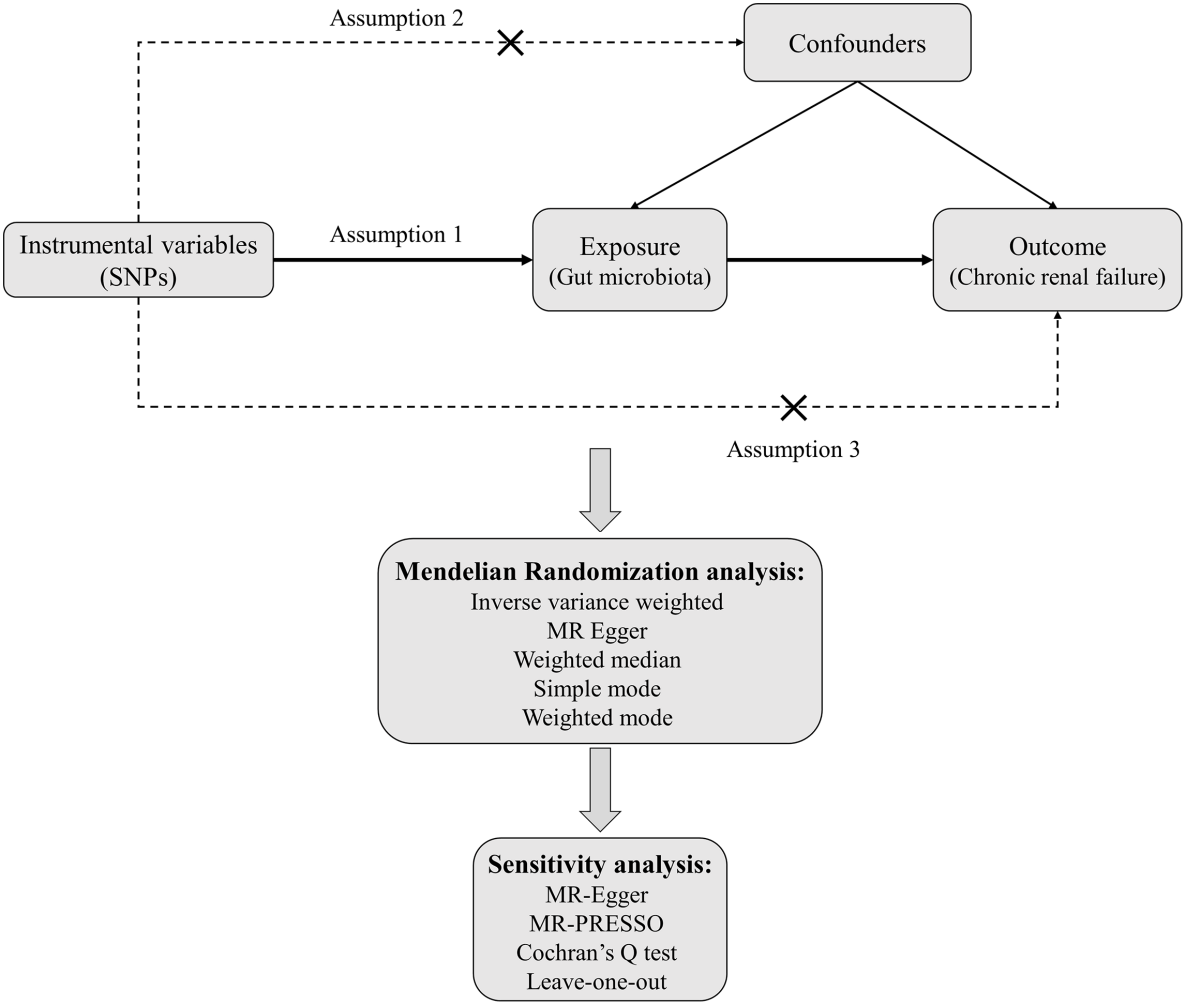
The flowchart for MR analysis between gut microbiota and CRF.

Mendelian randomization analysis must satisfy three critical assumptions: (1) Relevance assumption: the genetic variants chosen as instrumental variables should be associated with the exposure; (2) Independence assumption: the instrumental variables used should not be associated with any confounders; (3) Exclusion-restriction assumption: the instrumental variables must not have a direct effect on the outcome but should only influence the outcome through the exposure. In this study, gut microbiota is considered the exposure, and chronic renal failure is the outcome. Significant single-nucleotide polymorphisms (SNPs) are selected as instrumental variables (IVs). Two-sample Mendelian randomization methods are employed for the causal inference, supplemented by sensitivity analyses such as horizontal pleiotropy and heterogeneity tests. The specific study flowchart can be seen in Figure 1.

### 2.2 Data sources

The gut microbiota GWAS data are obtained from the MiBioGen consortium data (Kurilshikov et al., 2021).^1^ Researchers employed high-throughput gene sequencing technologies to analyze the gut microbiota of a large cohort of individuals, classifying the microbiota into respective taxonomic groups based on their genetic information by targeting three different variable regions (V4, V3–V4, and V1–V2) of the 16S rRNA gene. This dataset on gut microbiome comprises 24 cohorts, encompassing a total of 18,340 participants. Predominantly, the study population consisted of Europeans, accounting for more than 70% of the dataset (16 cohorts, *N* = 13,266). The GWAS data on CRF was sourced from the IEU OpenGWAS platform^2^ based on the research data by Sakaue et al. (2021). This CRF dataset included 482,858 individuals, with 8,287 cases and 474,571 controls, featuring 24,185,976 SNPs, with the study population being of European descent. These GWAS datasets are largely independent of each other, and all original research data have been ethically approved and consented to by the participants.

### 2.3 Instrumental variable

In this study, the gut microbiome GWAS data encompassed 211 taxonomic groups, including 131 genera, 35 families, 20 orders, 16 classes, and 9 phyla, with phyla representing the highest taxonomic level and genera being the lowest. The lower the taxonomic rank, the more common characteristics and similarities are shared among the microorganisms. Here, we conducted Mendelian randomization analyses using the genera level of gut microbiota, excluding 12 genera with unknown bacterial names (Kurilshikov et al., 2021), resulting in the inclusion of the remaining 119 genera in the study. The selection of suitable SNPs was based on the following criteria: (1) To ensure a sufficient number of instrumental variables, SNPs significantly associated at *p* < 1.0 × 10^−5^ were selected; (2) The chosen SNPs were required not to be in linkage disequilibrium with each other, generally measured by the parameters *r*^2^ and distance in kb. In this study, we set *r*^2^ < 0.001 and distance at 10,000 kb, thus removing SNPs within a 10,000 kb range that had an *r*^2^ > 0.001 with the most significant SNP to reduce the impact of linkage disequilibrium and retaining more independent SNPs; (3) The F-Statistic was used to assess the statistical strength of the association between SNPs and the exposure factor, where an *F* > 10 suggested a strong relationship with the exposure, and instrumental variables with an *F* < 10 were excluded. Finally, we harmonized SNPs with the same alleles in the GWAS data for gut microbiota and CRF, removing incompatible and palindromic SNPs to align the effect sizes for the exposure and outcome.

### 2.4 Statistical analysis

This study primarily utilized the “TwoSampleMR” package within R language (version 4.3.1) for analysis. The causal relationship between gut microbiota and CRF was assessed using five MR methods: Inverse Variance Weighted (IVW), MR-Egger, Weighted Median, Simple Mode, and Weighted Mode. Each statistical method relies on its specific model assumptions, and if these prerequisites are not met, the statistical power may be weakened or even rendered ineffective. The IVW method weights the effects between SNPs and causal traits by the inverse of their variance and combines them to estimate the overall effect. The results of IVW will be unbiased in the absence of horizontal pleiotropy (Burgess et al., 2015). The MR Egger method, based on the Egger regression in MR analyses, tests for horizontal pleiotropy through the MR-Egger regression. A significant intercept in the MR-Egger analysis, with *p* < 0.05, suggests the presence of horizontal pleiotropy. If the intercept is close to zero, the MR-Egger estimate will be close to the IVW estimate (Burgess and Thompson, 2017). The Weighted Median method can provide consistent estimates even if up to 50% of the SNP instruments are invalid, estimating the causal effect by weighting the effects of different SNPs (Bowden et al., 2016). The Simple Mode is a straightforward method of combining MR results by directly taking the mode of effect size estimates from different studies for a combined result. The Weighted Mode is an improved mode method that adds a step of weighting the effects of different studies on the basis of Simple Mode, calculating the combined result according to each study’s sample size or weight. Under certain conditions, the IVW method is more capable of effectively handling the synthesis of estimates, providing more precise results and demonstrating higher efficacy compared to other statistical methods (Bowden et al., 2016). Therefore, we primarily employed the IVW method for our analysis, with the remaining four methods serving as supplementary approaches. MR-PRESSO was utilized to detect significant outliers among the SNPs, and if the MR-PRESSO global test statistic *p* < 0.05, it indicates the presence of significant outliers that should be removed before repeating the MR analysis. Cochran’s Q test was applied to assess if there was significant heterogeneity among the SNPs. A statistically significant Cochran’s Q test (*p* < 0.05) indicates significant heterogeneity in the results, necessitating a discussion of the possible sources of heterogeneity. Leave-one-out sensitivity analysis was conducted, systematically excluding each SNP and calculating the combined effect of the remaining SNPs, to evaluate the impact of any single SNP on the relationship between exposure and outcome. Finally, the risk association between gut microbiota and CRF was expressed in terms of the odds ratio (OR) and its 95% confidence interval (CI), where *p* < 0.05 provided evidence for a potential causal relationship. Additionally, as the *p*-value threshold for SNP selection was set at *p* < 1.0 × 10^−5^, not meeting the conventional GWAS significance threshold of *p* < 1.0 × 10^−8^, False Discovery Rate (FDR) correction was applied for multiple testing to minimize the likelihood of type I statistical errors, setting the threshold for the FDR-adjusted *q*_value at 0.1. If *p* < 0.05 and *q*_value < 0.1, this suggests a significant association; if *p* < 0.05 but *q*_value > 0.1, it indicates an association of suggestive significance (Storey and Tibshirani, 2003).

## 3 Results

**Figure 2:**
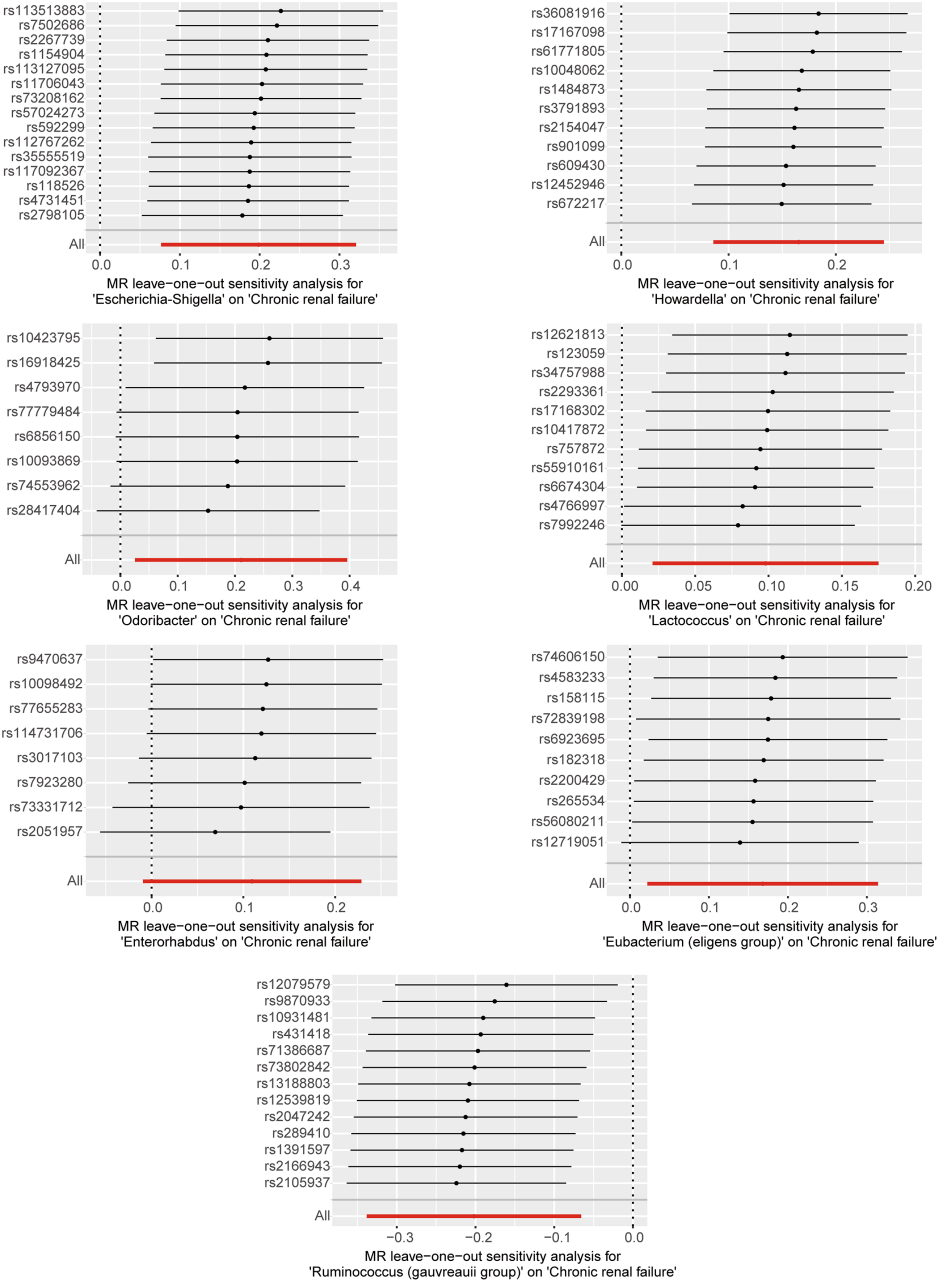
Leave-one-out plots for the causal effect of gut microbiota on CRF.

Based on the selection criteria for instrumental variables, a total of 1,480 SNPs were ultimately included as instrumental variables for MR analysis; all SNPs had an *F*-value greater than 10, and details can be found in Supplementary Table 1. MR analysis revealed that among the gut microbiota of 119 bacterial genera, according to the results of the IVW method (*p* < 0.05), 7 genera were closely related to the outcome variable CRF. Among these, Ruminococcus (gauvreauii group) (OR = 0.82, 95% CI = 0.71–0.94, *p* = 0.004) may be a protective factor for CRF. In contrast, Escherichia-Shigella (OR = 1.22, 95% CI = 1.08–1.38, *p* = 0.001), Lactococcus (OR = 1.1, 95% CI = 1.02–1.19, *p* = 0.013), Odoribacter (OR = 1.23, 95% CI = 1.03–1.49, *p* = 0.026), Enterorhabdus (OR = 1.14, 95% CI = 1.00–1.29, *p* = 0.047), Eubacterium (eligens group) (OR = 1.18, 95% CI = 1.02–1.37, *p* = 0.024), and Howardella (OR = 1.18, 95% CI = 1.09–1.28, *p* < 0.001) may be risk factors for CRF. However, after FDR correction, only the genera Escherichia-Shigella and Howardella had significant positive associations, while the other genera showed suggestive correlations. The detailed analysis results are available in Table 1. The complete MR analysis results of gut microbiota from 119 genera related to CRF can be found in Supplementary Table 2. Sensitivity analysis was conducted on the seven genera closely related to CRF, and based on the results of MR-Egger regression analysis and Cochran’s Q test, it was evident that there was no horizontal pleiotropy or heterogeneity among these genera, with the MR-PRESSO analysis yielding a global test *p* > 0.05, indicating no significant outliers. Specific results of the sensitivity analysis are presented in Table 2. The results of the pleiotropy and heterogeneity tests can be found in Supplementary Tables 3, 4. A leave-one-out analysis was performed for these seven genera (Figure 2), which showed that the results did not change significantly after sequentially removing individual SNPs, reflecting the robustness of the study findings to some extent. Scatter plots, Funnel plots, and Forest plots for these seven genera can be found in Supplementary Figures 1–3, respectively. Table 3 displays the relevant information of SNPs for the genera Escherichia-Shigella and Howardella used in this study. We further annotated the SNPs of these two genera using the VannoPortal database (Huang et al., 2022).

**Table 1:** MR results of causal links between gut microbiota and CRF.

Exposure (Bacterial taxa)	MR method	No. of snp	Beta	OR	95% CI	*p*-value	*q*_value
Escherichia-Shigella	MR egger	15	−0.06	0.94	0.65–1.35	0.74	
Escherichia-Shigella	Weighted median	15	0.15	1.16	0.98–1.37	0.09	
Escherichia-Shigella	Inverse variance weighted	15	0.20	1.22	1.08–1.38	<0.01	0.086
Escherichia-Shigella	Simple mode	15	0.34	1.41	1.03–1.92	0.05	
Escherichia-Shigella	Weighted mode	15	0.33	1.39	1.01–1.90	0.06	
Lactococcus	MR egger	11	0.06	1.06	0.73–1.54	0.77	
Lactococcus	Weighted median	11	0.09	1.09	0.98–1.21	0.11	
Lactococcus	Inverse variance weighted	11	0.10	1.10	1.02–1.19	0.01	0.377
Lactococcus	Simple mode	11	0.10	1.10	0.93–1.31	0.28	
Lactococcus	Weighted mode	11	0.08	1.09	0.93–1.27	0.31	
Odoribacter	MR egger	8	0.36	1.44	0.76–2.72	0.31	
Odoribacter	Weighted median	8	0.25	1.28	1.01–1.64	0.04	
Odoribacter	Inverse variance weighted	8	0.21	1.23	1.03–1.49	0.03	0.509
Odoribacter	Simple mode	8	0.25	1.29	0.88–1.87	0.23	
Odoribacter	Weighted mode	8	0.25	1.29	0.90–1.85	0.21	
Enterorhabdus	MR egger	7	−0.01	0.99	0.71–1.37	0.95	
Enterorhabdus	Weighted median	7	0.11	1.12	0.95–1.31	0.18	
Enterorhabdus	Inverse variance weighted	7	0.13	1.14	1.00–1.29	0.05	0.799
Enterorhabdus	Simple mode	7	0.04	1.04	0.82–1.32	0.75	
Enterorhabdus	Weighted mode	7	0.11	1.11	0.90–1.37	0.35	
Eubacterium (eligens group)	MR egger	10	0.07	1.07	0.72–1.61	0.74	
Eubacterium (eligens group)	Weighted median	10	0.14	1.15	0.95–1.39	0.16	
Eubacterium (eligens group)	Inverse variance weighted	10	0.17	1.18	1.02–1.37	0.02	0.573
Eubacterium (eligens group)	Simple mode	10	0.09	1.10	0.82–1.47	0.55	
Eubacterium (eligens group)	Weighted mode	10	0.10	1.10	0.88–1.39	0.42	
Ruminococcus (gauvreauii group)	MR egger	13	−0.59	0.55	0.30–1.03	0.09	
Ruminococcus (gauvreauii group)	Weighted median	13	−0.14	0.87	0.72–1.06	0.16	
Ruminococcus (gauvreauii group)	Inverse variance weighted	13	−0.20	0.82	0.71–0.94	<0.01	0.145
Ruminococcus (gauvreauii group)	Simple mode	13	−0.10	0.90	0.65–1.25	0.54	
Ruminococcus (gauvreauii group)	Weighted mode	13	−0.11	0.90	0.68–1.19	0.47	
Howardella	MR egger	11	−0.04	0.97	0.69–1.36	0.84	
Howardella	Weighted median	11	0.18	1.20	1.08–1.33	<0.01	
Howardella	Inverse variance weighted	11	0.17	1.18	1.09–1.28	<0.01	0.005
Howardella	Simple mode	11	0.22	1.25	1.03–1.51	0.04	
Howardella	Weighted mode	11	0.21	1.23	1.02–1.49	0.05	

MR, Mendelian randomization; CRF, chronic renal failure; SNP, single nucleotide polymorphism; OR, odds ratio; CI, confidence interval.

**Table 2:** Sensitivity analysis results.

Exposure (bacterial taxa)	Pleiotropy test			Heterogeneity test		
	Egger intercept	*p*-value	MR-PRESSO	Method	Cochran’s Q	Q_pval
Howardella	0.03	0.27	0.68	IVW	8.21	0.61
Escherichia-Shigella	0.02	0.16	0.85	IVW	8.73	0.85
Ruminococcus (gauvreauii group)	0.03	0.23	0.63	IVW	10.31	0.59
Eubacterium (eligens group)	0.01	0.63	0.90	IVW	4.43	0.88
Enterorhabdus	0.01	0.73	0.63	IVW	5.53	0.60
Odoribacter	−0.01	0.64	0.49	IVW	6.79	0.45
Lactococcus	0.01	0.83	0.55	IVW	9.41	0.49

IVW, inverse variance weighted.

**Table 3:** Summary information on SNPs of Escherichia-Shigella and Howardella used for the Mendelian randomization analyses.

Bacterial taxa	SNP	Effect allele	Other allele	EAF	Chr	Position*	Gene	*p*-value
Escherichia-Shigella	rs112767262	T	C	0.196	16	3,750,623	TRAP1	8.21E-06
Escherichia-Shigella	rs113127095	A	G	0.052	13	23,253,392	MTND3P1/FTH1P7	3.33E-06
Escherichia-Shigella	rs113513883	A	G	0.058	7	140,678,199	CCT4P1/RP4-813F11.3	5.28E-06
Escherichia-Shigella	rs1154904	A	G	0.464	11	134,774,845	B3GAT1/AP003062.1	3.04E-06
Escherichia-Shigella	rs11706043	T	A	0.157	3	3,173,016	TRNT1	5.87E-06
Escherichia-Shigella	rs117092367	A	T	0.089	10	69,394,914	CTNNA3	9.65E-06
Escherichia-Shigella	rs118526	C	A	0.393	5	79,863,544	ANKRD34B	8.00E-06
Escherichia-Shigella	rs2267739	G	C	0.086	7	31,136,897	ADCYAP1R1	1.42E-06
Escherichia-Shigella	rs2798105	A	G	0.118	1	48,961,903	SPATA6/RP11-329A14.2	8.25E-06
Escherichia-Shigella	rs35555519	C	G	0.114	16	77,711,641	ADAMTS18/NUDT7	4.92E-06
Escherichia-Shigella	rs4731451	G	A	0.372	7	128,054,100	IMPDH1/RP11-155G14.1	7.47E-06
Escherichia-Shigella	rs57024273	T	C	0.318	2	236,515,155	AGAP1	9.70E-06
Escherichia-Shigella	rs592299	T	C	0.468	9	136,179,347	LCN1P1/LCN1P2	4.77E-06
Escherichia-Shigella	rs73208162	A	G	0.059	21	39,324,484	DSCR4	2.19E-06
Escherichia-Shigella	rs7502686	G	C	0.063	17	14,173,290	HS3ST3B1/CDRT15	5.90E-06
Howardella	rs10048062	C	T	0.107	15	98,476,920	ARRDC4	8.59E-06
Howardella	rs12452946	A	G	0.501	17	17,253,288	NT5M/RPL13P12	3.80E-06
Howardella	rs1484873	A	G	0.069	18	43,206,985	SLC14A2	2.56E-06
Howardella	rs17167098	G	A	0.113	7	133,154,356	EXOC4	1.12E-06
Howardella	rs2154047	C	A	0.068	14	95,452,797	DICER1/RPL15P2	9.97E-06
Howardella	rs36081916	T	C	0.111	7	93,527,414	GNGT1	4.70E-06
Howardella	rs3791893	A	G	0.123	2	218,819,396	TNS1	9.50E-06
Howardella	rs609430	T	G	0.339	4	169,178,315	DDX60	3.34E-06
Howardella	rs61771805	A	T	0.187	1	72,972,799	RPL31P12/KRT8P21	4.03E-06
Howardella	rs672217	G	A	0.141	18	60,125,134	ZCCHC2/ACTBP9	3.52E-06
Howardella	rs901099	T	G	0.312	11	77,901,287	USP35	6.53E-07

SNP, single nucleotide polymorphism; EAF, effect allele frequency; Chr, chromosome; *Position based on GRCh37.

## 4 Discussion

Previous studies on the gut microbiome and CKD have primarily utilized animal models (Vaziri et al., 2013), with observations often showing inconsistencies. While there have been reports on the causal relationship between the gut microbiome and CKD (Li et al., 2023; Ren et al., 2023), significant variations in the richness and evenness (α-diversity) of bacterial species across different CKD stages have been observed (Wu et al., 2020). CRF represents a more severe stage of kidney pathology. This study focuses on the later stages of the disease, CRF, and evaluates the causal relationship between the gut microbiota of 119 genera and CRF at a genetic level, utilizing large-scale GWAS data. After MR analysis and FDR correction of the results, we ultimately discovered that Escherichia-Shigella and Howardella may be risk factors for CRF, as they exhibited a significant association with CRF.

Escherichia and Shigella both belong to the family Enterobacteriaceae, which is part of the phylum Proteobacteria, and are Gram-negative rod-shaped bacteria. In many classification systems, Escherichia and Shigella are treated as separate genera; however, due to their high genomic similarity and the potential inaccuracy of current metagenomic sequencing techniques in distinguishing members of the two genera, some bioinformatics analyses classify them as a single genus, especially in genomic studies of microbial communities, where these two genera are commonly represented together as “Escherichia-Shigella.” Certain strains of the Escherichia-Shigella genus are pathogenic, and thus this genus is often reported as a pathogen (Kamada et al., 2013). The genus Escherichia is most commonly represented by *Escherichia coli* (Tenaillon et al., 2010). It has been reported that Shiga toxin-producing *E. coli* can cause classic hemolytic uremic syndrome (HUS), which is the most common cause of acute renal failure in children. This condition is caused by endothelial cell damage and leukocyte activation due to the production of Shiga-like toxins and lipopolysaccharides, leading to the formation of glomerular capillary thrombosis through the interaction of platelets with endothelial cells, ultimately affecting renal hemodynamics and causing acute kidney injury (Palermo et al., 2014; Fakhouri et al., 2017). CRF is also one of the complications of HUS (Razzaq, 2006). Shigella typically causes bacterial dysentery, also known as Shigellosis, and is genetically related to *E. coli* (Beld and Reubsaet, 2011). Wu et al. (2020) discovered through 16S rRNA gene sequencing that the genus Escherichia-Shigella is associated with various stages of CKD, particularly enriched in CRF, and that the abundance of Escherichia-Shigella is highly correlated with the levels of serum Indoxyl Sulfate. Research by Hu X. et al. (2020) indicates an increase in the quantities of the genus Escherichia-Shigella and the phylum Proteobacteria in the gut of CRF patients, especially prominent at stage 5 of the disease, with several genera including Escherichia-Shigella showing high sensitivity and specificity in distinguishing CKD patients from healthy controls. Furthermore, Bao et al. (2023) also found a close association between the genus Escherichia-Shigella and vascular calcification in hemodialysis patients, identifying it as a risk factor for vascular calcification in these patients.

Howardella is a Gram-positive anaerobic bacterium that can produce ATP by decomposing urea (Cook et al., 2007). However, while some gut microbes hydrolyze urea through urease, they can generate large amounts of ammonia, which may affect the growth of the intestinal microbiota (Ramezani and Raj, 2014). Moreover, increased levels of ammonia are associated with tubulointerstitial damage in the kidneys. The rise in renal ammonia levels can potentially activate the complement alternative pathway, resulting in the production of chemotactic and lytic complement components that lead to tubulointerstitial inflammation (Nath et al., 1985). Additionally, the production of ammonia by the intestinal microbiota through urea hydrolysis can also lead to the disruption of the structure and function of the intestinal epithelium. Consequently, this promotes the translocation of uremic toxins, endotoxins, antigens, and other microbial metabolites into the circulation, which could be an important pathway for endogenous infections (Simões-Silva et al., 2018). Studies have reported an increase in the abundance of the genus Howardella associated with prediabetes (Yang et al., 2015), while a lower abundance of Howardella has been observed in the gut of patients with depression (Barandouzi et al., 2020). Furthermore, Yang et al. (2023) found a positive correlation between exposure to nitrogen dioxide and the abundance of the genus Howardella. However, there are limited reports on the association between the genus Howardella and CRF, warranting further in-depth research in the future.

Increasing research indicates the presence of gut microbiota dysbiosis in patients with CRF. Vaziri et al. (2013) observed a significant increase in the abundance of genera such as Brachybacterium and Catenibacterium in CRF patients, while Jiang et al. found a decrease in the abundance of butyrate-producing bacteria, including Roseburia, Faecalibacterium, Clostridium, Coprococcus, and Prevotella in CRF patients. They also discovered a negative correlation between bacteria such as Roseburia spp., Faecalibacterium, Prevotella, and markers like C-reactive protein and Cystatin C (Jiang et al., 2017). Furthermore, a systematic review reported a decrease in microbial diversity in CKD patients compared to healthy individuals, with a consistent reduction in Roseburia abundance, especially in patients with end-stage kidney disease (Voroneanu et al., 2023). An increase in the abundance of the phylum Proteobacteria is a potential microbial diagnostic marker of dysbiosis (Shin et al., 2015), which is mainly associated with an increase in the Enterobacteriaceae family and the genus Escherichia. The inflammation-driven overgrowth of Enterobacteriaceae is closely linked to the development of various host diseases (Lupp et al., 2007). The findings on the gut microbiota of CRF patients are varied, possibly limited by the nature of observational studies, and these studies have not further explored their causal relationships. This study addresses the gap by investigating the causal relationship between bacterial genera and CRF at the genus level, addressing the current inadequacies in gut microbiota research in CRF. However, the mechanisms of action remain unclear. Based on the gut-kidney axis concept and current research, we hypothesize that the mechanisms of action of the gut microbiota on CRF relate to the following aspects: (1) Microbiome: The microbial communities within the gut can affect intestinal health and the systemic inflammatory state, and they can influence renal function through their metabolic products. (2) Metabolic Products: Urea and other nitrogenous wastes produced in the gut can affect kidney health through the circulatory system. (3) Mucosal Barrier Function and Intestinal Permeability: Impairment of the gut barrier may allow bacteria and endotoxins access to the bloodstream, from where they can reach the kidneys and potentially cause inflammatory responses. (4) Immune System: Immune cells in the gut may impact kidney function by promoting systemic inflammatory responses. Therefore, correcting gut microbial dysbiosis, improving gut barrier function, and reducing endotoxin load, among other measures, may contribute to better kidney health and slow the progression of CRF.

In the gut of CRF patients, there is a significant increase in the number of bacterial families capable of producing urease as well as enzymes that generate indoles and p-cresol, whereas the number of bacterial families that can convert dietary fiber into short-chain fatty acids (such as butyrate) significantly decreases (Wong et al., 2014). Urease can break down urea into ammonia and carbon dioxide. In CRF patients, this could lead to excessive production of ammonia, thereby increasing the concentration of ammonia in the gut and blood, potentially exacerbating symptoms of uremia. Enzymes that produce indoles and p-cresol are involved in the metabolism of tryptophan and phenolic compounds in the intestine, ultimately generating toxic substances to the human body, such as Indoxyl Sulfate (IS) and p-Cresyl Sulfate (pCS). IS and pCS are metabolites in the blood associated with renal failure. They are commonly used as biomarkers to measure kidney function and damage extent. Studies have shown that high levels of IS and pCS play a crucial role in the progression of CKD, with their accumulation closely linked to accelerated renal fibrosis, reduced kidney function, and CKD progression (Meijers and Evenepoel, 2011; Mutsaers et al., 2015). Indoxyl sulfate can trigger cellular oxidative stress response by generating reactive oxygen species, activate NF-κB promoting cellular senescence, and activate p53 accelerating the aging process of proximal tubular cells, ultimately exacerbating CRF progression (Shimizu et al., 2011). In CRF patients, due to renal function decline, the accumulation of these compounds in the serum further aggravates disease progression. The accumulation of these toxins causes damage not only to the kidneys but also to other tissues and organs of the body. Short-chain fatty acids (SCFAs), particularly butyrate, are extremely important for intestinal health, providing energy for intestinal cells, regulating local and systemic immune responses, and promoting the integrity of the gut barrier. In CRF patients, the reduction in this probiotic conversion capability may impact gut health, increase inflammation and intestinal permeability, thereby further affecting the overall health of the patients. Gut microbiota dysbiosis, especially in the context of kidney disease, by reducing beneficial microbes producing SCFAs and increasing protein-fermenting microbes, may lead to the production of uremic toxins, endotoxemia, systemic inflammation, and various complications associated with CKD, thus exacerbating the progression of kidney disease (Pan and Kang, 2017). However, research by Mitrović et al. has shown that the use of Synbiotic can effectively reduce the serum levels of Indoxyl Sulfate and high-sensitivity C-reactive protein in patients with CRF. This finding supports the effectiveness of Synbiotic targeted gut therapy in reducing uremic toxin levels and improving the micro-inflammatory state in CRF patients, proving it to be a safe and effective treatment strategy (Mitrović et al., 2023). This indicates that correcting gut microbiota dysbiosis, improving gut barrier function, and reducing endotoxin load can all contribute to improving kidney health and delaying the progression of CRF.

It is noteworthy that the gut microbiota encompasses bacteria, fungi, viruses, archaea, and other microorganisms, together constituting a complex ecosystem. Bacteria are the most abundant microorganisms in the gut microbiota, and hence, current research on gut microbiota primarily focuses on the bacterial component. However, other microorganisms can also influence the health and disease states of the host under certain conditions.

The gut mycobiome, a component of the gut microbiome, plays an indispensable role in maintaining the balance of the intestinal environment and host health, despite fungi being less numerous than bacteria in the gut. Research by Qiu et al. has identified significant alterations in the composition of the gut fungi in individuals with both hypertension and CKD. Compared to healthy controls, this specific population exhibited increased richness and diversity of gut fungi, particularly elevated levels of the genera Apiotrichum and Saccharomyces, while the abundance of Candida decreased. Further analysis revealed a negative correlation between Candida and tumor necrosis factor alpha (TNF-α), and a positive correlation was found between the filtration rate of the glomeruli and the genera Apiotrichum, Ophiocordyceps, Saccharomyces, Nakaseomyces, and Septoria (Qiu et al., 2023). These findings suggest that these specific gut fungi may play an important role in protecting kidney function. Research by Ren et al. also found that patients with ESRD have a higher diversity of gut fungi compared to healthy individuals, characterized by a decrease in *Saccharomyces cerevisiae* and an increase in various opportunistic pathogens (such as *Aspergillus fumigatus*, Exophiala spinifera, Hortaea werneckii, etc.). Further metabolomic analysis revealed that the enriched opportunistic pathogens correlated positively with levels of serum creatinine, homocysteine, and phenylacetylglycine, while the yeast community negatively correlated with the levels of toxic metabolites in feces (Ren et al., 2024). Additionally, studies have shown a certain link between the risk of inflammatory bowel disease and CKD (Vajravelu et al., 2020), while *Saccharomyces cerevisiae* has been found to inhibit and reduce chemically induced colonic inflammation (Sivignon et al., 2015). Hu et al. (2022) also discovered a correlation between yeast and serum levels of gamma light chains. These findings indicate that the gut mycobiome is closely associated with the inflammatory state, immune response, and toxin levels in patients with CRF.

Viral infections are involved in a variety of glomerular diseases (Sallustio et al., 2023). The gut virome, particularly the part dominated by bacteriophages, plays a crucial role in the ecological balance of gut microbiota. These bacteriophages, by invading and replicating within bacterial hosts, not only regulate the population of specific bacterial groups to maintain microbial diversity but also significantly influence the host’s immune system regulation. Studies have reported that bacteriophages can activate the host’s immune response via the TLR9 sensing pathway, promoting the production of cytokines such as IL-12, IL-6, IL-10, and IFN-γ (Gogokhia et al., 2019; Sweere et al., 2019). Bacteriophages can also enter the systemic circulation through intestinal epithelial cells and then affect the human immune system by modulating the release of cytokines and the activity of T and B cells (Popescu et al., 2021). Research by Fan et al. (2023) revealed an imbalance in the gut virome in patients with diabetic nephropathy (DN), characterized by a significant decrease in overall viral richness and diversity. Notably, levels of Shigella phage and Xylella phage were elevated in the guts of DN patients. Further analysis of the functional aspects of the gut virome found multiple viral functional losses in DN patients, particularly a weakened ability of bacteriophages to lyse their bacterial hosts. This reduction in function could have important implications for the ecological balance of the gut microbiota.

Archaea represent a unique form of life, distinct from bacteria and eukaryotes, possessing special biological characteristics and metabolic pathways. Despite their relatively low abundance in the human body, archaea play a crucial role in maintaining human health and environmental balance (Mafra et al., 2022). Particularly in the human gut, methanogenic archaea, such as *Methanobrevibacter smithii*, dominate. These archaea not only promote the development of other microbes by consuming hydrogen, maintaining the stability and diversity of the gut microbial community, but also regulate the structure and function of the gut microbiome through their unique metabolic products (e.g., methane), significantly impacting host health. Trimethylamine-N-oxide (TMAO) has been shown to promote oxidative stress, inflammation, and the progression of CKD (Borges et al., 2016; Castillo-Rodriguez et al., 2018), and is an independent predictor of the burden of coronary artery atherosclerosis and mortality (Stubbs et al., 2016). Research has reported that methanogenic archaea can reduce the conversion of trimethylamine (TMA) to TMAO (Brugère et al., 2013), thereby decreasing the production of TMAO associated with health issues such as cardiovascular diseases CKD, and trimethylaminuria. Therefore, modulating the metabolic activity of archaea, especially increasing beneficial archaeal populations, may help mitigate inflammation and treat related disease states. This reveals the potential positive role of archaea in regulating the gut microbiome, reducing the generation of harmful metabolic products, and combating chronic inflammation and related diseases through possible other mechanisms, such as modulating host immune responses, offering a new perspective for the treatment of related diseases.

In summary, our study addresses the current gaps in research on the gut microbiota in CRF and identifies gut genera with potential causal relationships to CRF, offering more possibilities for future targeted gut interventions to prevent or treat CRF. However, our research has certain limitations. Firstly, since our data were derived from public databases, which only provided aggregated data related to single nucleotide polymorphisms without information on individual confounding factors such as age, gender, and antibiotic usage, we were unable to conduct more detailed subgroup analyses. Secondly, our study merely infers the causal relationship between gut microbiota and CRF from a theoretical perspective, with a focus on the correlation analysis between gut genera and CRF. The gut microbiome is a complex system influenced by diet, medication, environment, and other factors (Stepkowski et al., 2017; Asnicar et al., 2021). Future large-scale randomized controlled trials incorporating metagenomics, metabolomics, genomics, and *in vitro* cultures are needed to further investigate its specific mechanisms of action. Lastly, the GWAS data we used primarily involved European populations, which may not be generalizable to other ethnic groups. Moreover, our exposure data focused on the genus level of microbial communities, indicating that future research should delve deeper into species or strain-level analyses. We believe that as research progresses, modern medicine may develop new therapeutic strategies to prevent or treat various kidney diseases, including CRF, by improving the interaction between the gut and the kidney axis.

## 5 Conclusion

The results of this study suggest that the genera Escherichia-Shigella and Howardella are potential risk factors for CRF, highlighting their significance in CRF. Future interventions for patients with CRF could potentially include dietary modifications, probiotics, and fecal transplantation to slow the progression of the disease. However, our research can only infer part of the causation. The onset and progression of CRF are also related to many other factors beyond the gut microbiota, and further in-depth, large-scale studies are needed to fully elucidate their specific mechanisms of action.

## Data availability statement

The original contributions presented in the study are included in the article/Supplementary material, further inquiries can be directed to the corresponding author.

## Author contributions

XL: Conceptualization, Data curation, Methodology, Writing – original draft, Writing – review & editing. JM: Data curation, Methodology, Writing – review & editing. XY: Formal analysis, Methodology, Writing – review & editing. LP: Conceptualization, Writing – review & editing. YoZ: Formal analysis, Writing – review & editing. YiZ: Supervision, Writing – review & editing. GS: Supervision, Writing – review & editing.

## Footnotes

^1^ https://mibiogen.gcc.rug.nl/

^2^ https://gwas.mrcieu.ac.uk/
