# Supplementary material for: Causal relationship between gut microbiota and chronic renal failure: a two-sample Mendelian randomization study
Source: Front Microbiol. 2024 Apr 3;15:1356478. doi: 10.3389/fmicb.2024.1356478 (PMC11021586; doi:10.3389/fmicb.2024.1356478)
Supplement: Supplementary file 1 [file Table_1.DOCX]

Supplementary Material

# Supplementary Figures and Tables

## Supplementary Figures


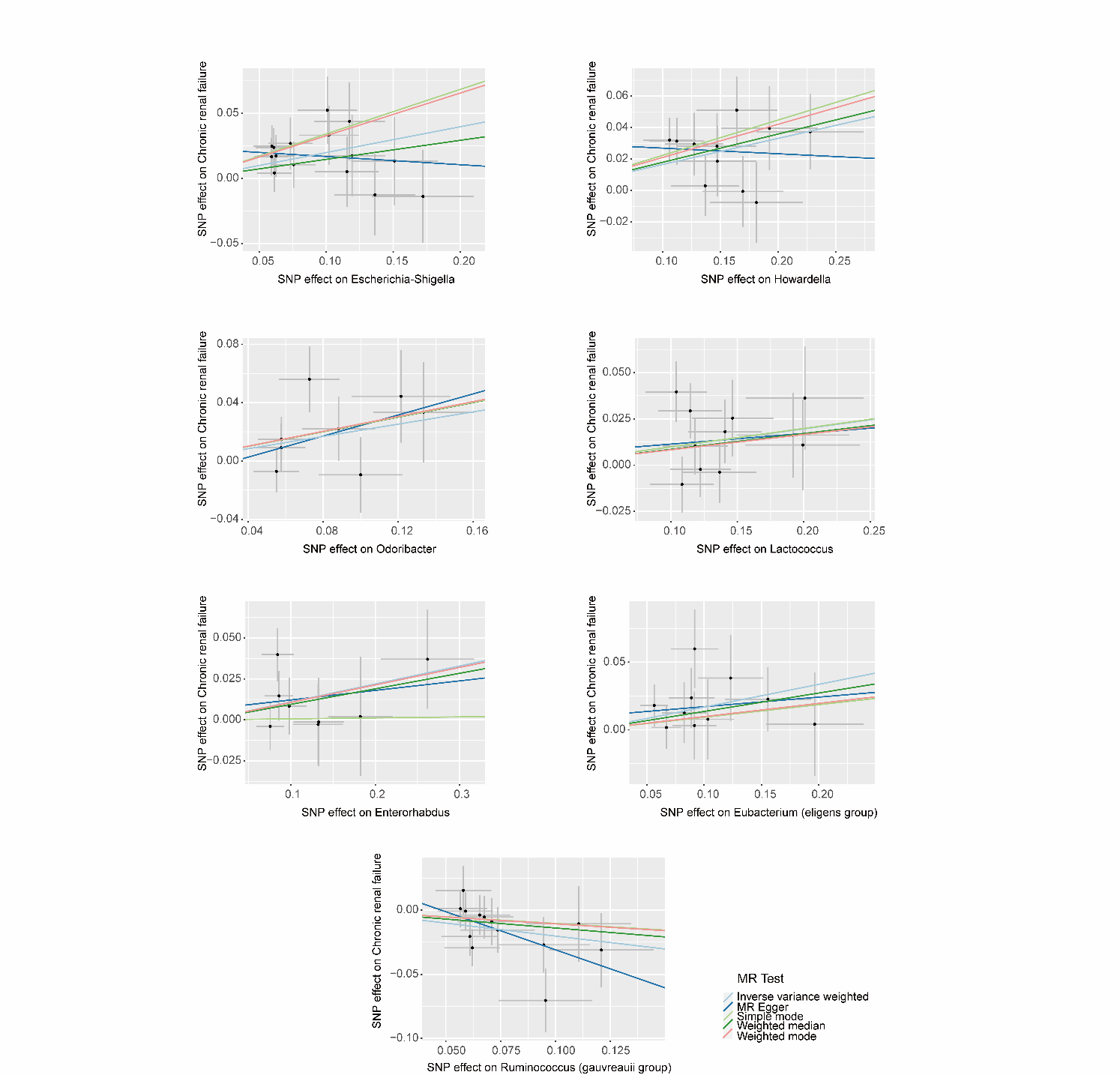


**Supplementary Figure 1.** Scatter plots for the causal effect of gut microbiota on CRF.

**
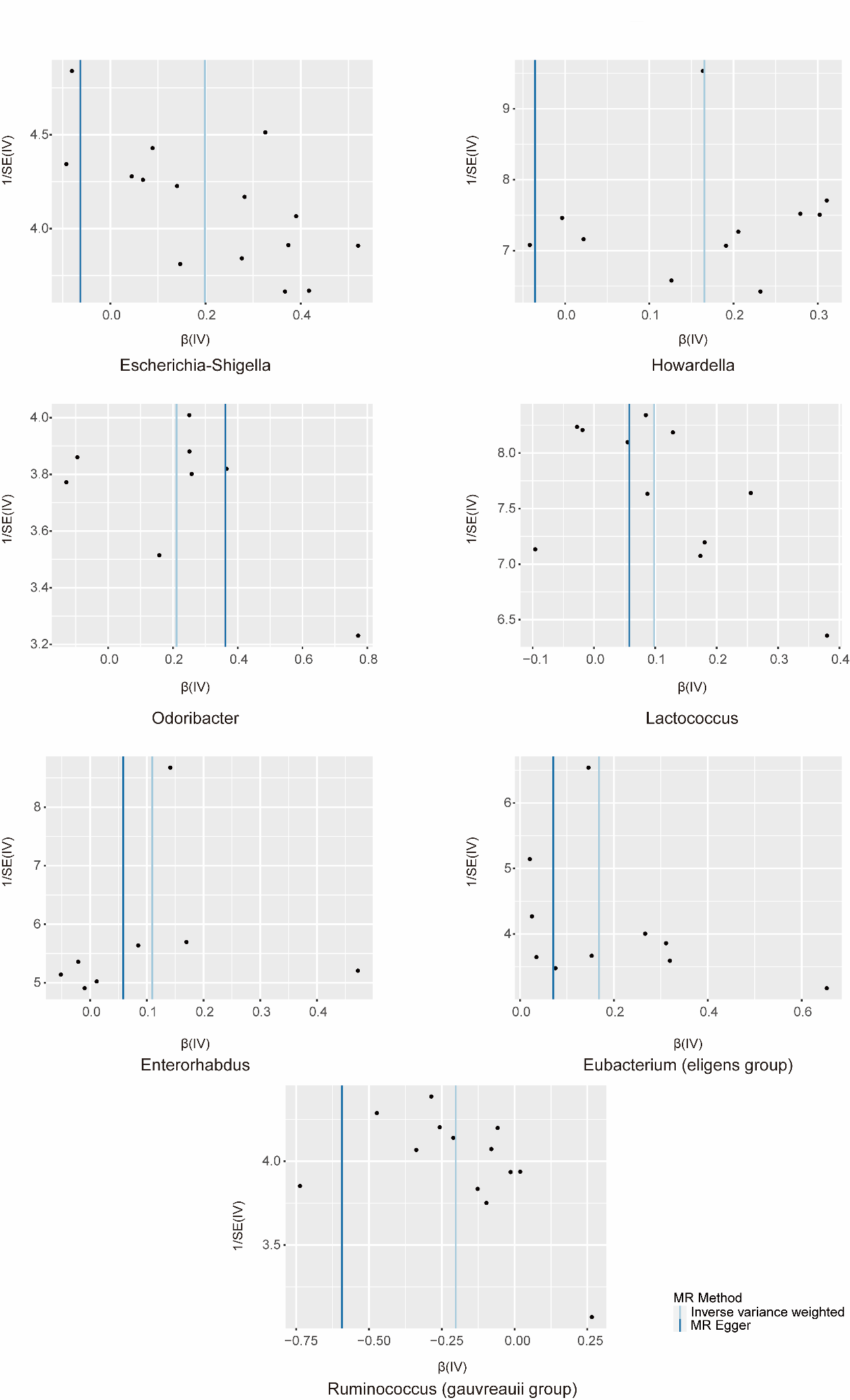
**

**Supplementary Figure 2.** Funnel plots for the causal effect of gut microbiota on CRF.


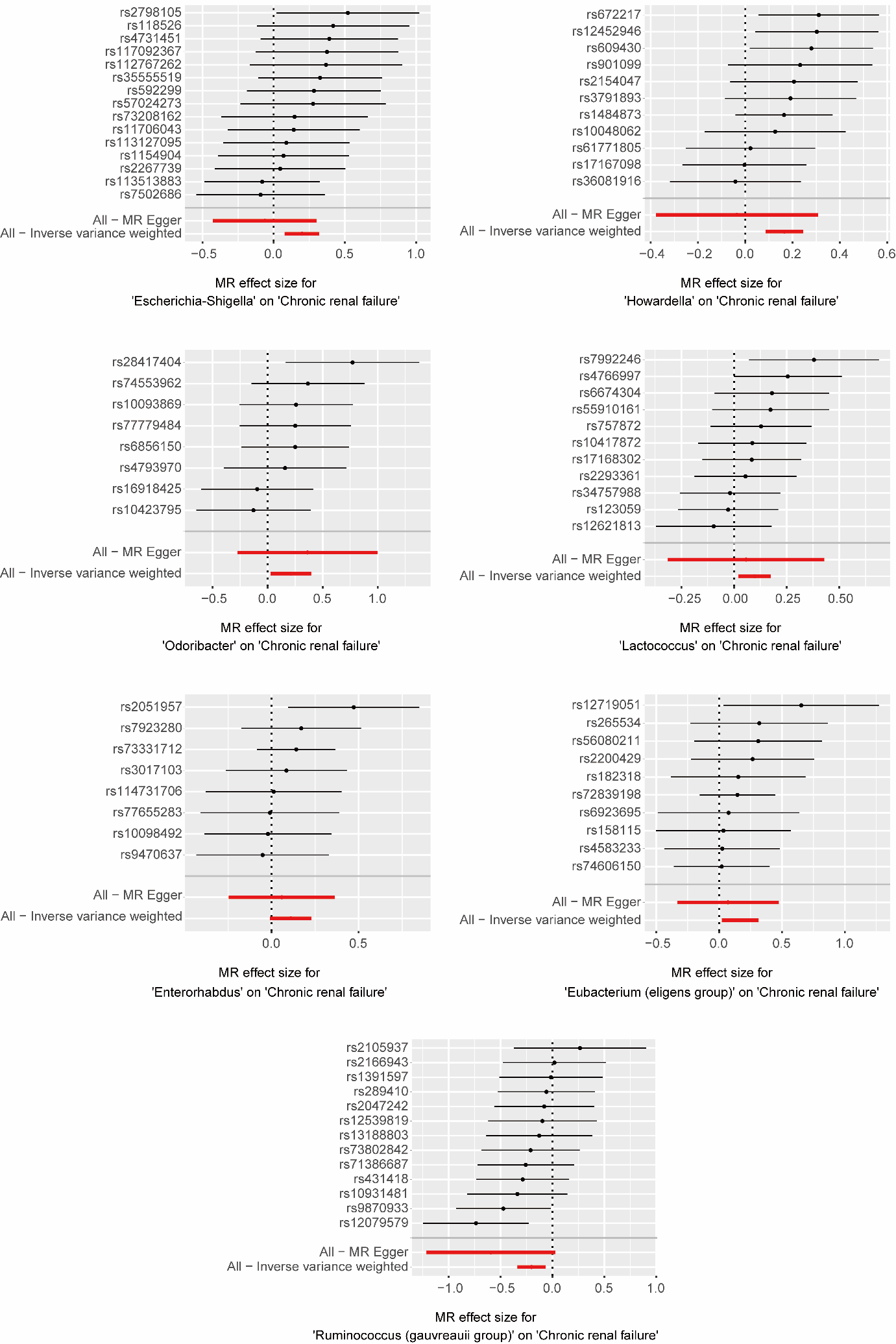


**Supplementary Figure 3.** Forest plots for the causal effect of gut microbiota on CRF.

## Supplementary Tables

**Supplementary Table 1** Instrumental variables used in MR analysis of the association between gut microbiota and CRF.

| **Bacterial taxa** | **SNP** | **Effect allele** | **Other allele** | **EAF** | **Exposure(gut microbiota)** | | | **Outcome(chronic renal failure)** | | |
| --- | --- | --- | --- | --- | --- | --- | --- | --- | --- | --- |
|  |  |  |  |  | **Beta** | **SE** | **P-value** | **Beta** | **SE** | **P-value** |
| Intestinimonas | rs1000888 | C | G | 0.396 | -0.059 | 0.013 | 9.71E-06 | 0.018 | 0.015 | 0.23 |
| Intestinimonas | rs10262702 | T | C | 0.129 | 0.092 | 0.019 | 2.06E-06 | 0.060 | 0.019 | 0.00 |
| Intestinimonas | rs11258178 | A | G | 0.415 | 0.066 | 0.013 | 6.98E-07 | 0.016 | 0.015 | 0.29 |
| Intestinimonas | rs12226153 | A | G | 0.062 | -0.151 | 0.031 | 5.12E-07 | -0.025 | 0.036 | 0.49 |
| Intestinimonas | rs12566247 | T | A | 0.348 | 0.064 | 0.014 | 2.19E-06 | -0.013 | 0.015 | 0.36 |
| Intestinimonas | rs17067892 | C | T | 0.077 | 0.107 | 0.025 | 6.38E-06 | 0.021 | 0.029 | 0.48 |
| Intestinimonas | rs1859797 | G | A | 0.504 | 0.060 | 0.013 | 4.12E-06 | -0.008 | 0.015 | 0.61 |
| Intestinimonas | rs2276760 | A | G | 0.220 | -0.069 | 0.015 | 7.84E-06 | 0.000 | 0.017 | 0.99 |
| Intestinimonas | rs2731794 | C | T | 0.060 | 0.121 | 0.026 | 1.92E-06 | 0.015 | 0.028 | 0.58 |
| Intestinimonas | rs2930225 | G | T | 0.258 | 0.073 | 0.015 | 1.35E-06 | 0.051 | 0.017 | 0.00 |
| Intestinimonas | rs4113676 | A | C | 0.062 | -0.219 | 0.049 | 7.42E-06 | 0.001 | 0.041 | 0.99 |
| Intestinimonas | rs4784055 | T | C | 0.056 | -0.175 | 0.039 | 8.72E-07 | -0.007 | 0.025 | 0.78 |
| Intestinimonas | rs62240188 | G | A | 0.069 | 0.130 | 0.027 | 2.20E-06 | -0.021 | 0.029 | 0.47 |
| Intestinimonas | rs62427239 | C | A | 0.046 | 0.163 | 0.037 | 9.41E-06 | 0.011 | 0.041 | 0.78 |
| Intestinimonas | rs6934519 | C | T | 0.275 | 0.069 | 0.015 | 8.57E-06 | 0.025 | 0.017 | 0.15 |
| Intestinimonas | rs716604 | A | G | 0.188 | 0.082 | 0.017 | 8.57E-07 | 0.001 | 0.019 | 0.94 |
| Intestinimonas | rs7170984 | T | C | 0.290 | -0.066 | 0.014 | 2.98E-06 | 0.022 | 0.016 | 0.17 |
| Intestinimonas | rs72982915 | C | T | 0.046 | 0.183 | 0.040 | 4.91E-06 | -0.038 | 0.034 | 0.26 |
| Intestinimonas | rs9823439 | T | C | 0.441 | -0.058 | 0.013 | 9.86E-06 | -0.030 | 0.015 | 0.05 |
| Intestinimonas | rs994794 | G | C | 0.040 | -0.142 | 0.032 | 7.31E-06 | -0.064 | 0.035 | 0.07 |
| Streptococcus | rs10028567 | C | T | 0.082 | -0.092 | 0.019 | 7.30E-06 | 0.024 | 0.022 | 0.26 |
| Streptococcus | rs10448310 | A | G | 0.430 | -0.052 | 0.011 | 3.31E-06 | -0.047 | 0.014 | 0.00 |
| Streptococcus | rs11110281 | T | C | 0.068 | -0.138 | 0.023 | 2.58E-09 | 0.036 | 0.033 | 0.27 |
| Streptococcus | rs11720390 | G | A | 0.075 | 0.107 | 0.023 | 3.59E-06 | 0.021 | 0.030 | 0.49 |
| Streptococcus | rs11764382 | A | G | 0.191 | -0.070 | 0.014 | 1.29E-06 | 0.003 | 0.019 | 0.89 |
| Streptococcus | rs17708276 | A | G | 0.128 | -0.079 | 0.017 | 3.04E-06 | -0.042 | 0.022 | 0.06 |
| Streptococcus | rs1918540 | G | A | 0.253 | 0.060 | 0.013 | 2.44E-06 | -0.016 | 0.019 | 0.38 |
| Streptococcus | rs2370083 | G | T | 0.089 | -0.082 | 0.019 | 9.75E-06 | 0.024 | 0.025 | 0.35 |
| Streptococcus | rs395407 | G | C | 0.142 | -0.079 | 0.017 | 4.37E-06 | 0.000 | 0.021 | 1.00 |
| Streptococcus | rs4968759 | A | G | 0.426 | -0.052 | 0.011 | 3.78E-06 | -0.001 | 0.017 | 0.97 |
| Streptococcus | rs57646748 | G | A | 0.068 | -0.091 | 0.020 | 5.48E-06 | 0.014 | 0.023 | 0.56 |
| Streptococcus | rs6563952 | G | C | 0.092 | 0.083 | 0.018 | 5.82E-06 | 0.006 | 0.022 | 0.78 |
| Streptococcus | rs6806351 | T | C | 0.209 | -0.063 | 0.014 | 4.94E-06 | 0.012 | 0.018 | 0.51 |
| Streptococcus | rs71481756 | T | G | 0.099 | 0.093 | 0.021 | 6.51E-06 | 0.001 | 0.028 | 0.98 |
| Streptococcus | rs72739637 | A | G | 0.106 | 0.096 | 0.019 | 1.03E-06 | -0.001 | 0.029 | 0.96 |
| Streptococcus | rs7916711 | A | G | 0.065 | 0.103 | 0.022 | 2.72E-06 | 0.041 | 0.022 | 0.06 |
| Streptococcus | rs9903102 | C | A | 0.164 | -0.071 | 0.016 | 4.18E-06 | 0.002 | 0.026 | 0.95 |
| Peptococcus | rs10031059 | T | C | 0.221 | -0.121 | 0.023 | 1.24E-07 | -0.013 | 0.016 | 0.42 |
| Peptococcus | rs11001941 | G | A | 0.068 | -0.196 | 0.039 | 1.33E-06 | 0.008 | 0.028 | 0.78 |
| Peptococcus | rs11030569 | A | T | 0.078 | -0.174 | 0.037 | 3.13E-06 | -0.013 | 0.029 | 0.66 |
| Peptococcus | rs12069354 | C | T | 0.067 | 0.168 | 0.038 | 9.28E-06 | -0.017 | 0.032 | 0.61 |
| Peptococcus | rs2054133 | G | A | 0.474 | 0.090 | 0.019 | 2.14E-06 | -0.012 | 0.015 | 0.41 |
| Peptococcus | rs34282744 | G | C | 0.062 | 0.192 | 0.040 | 1.84E-06 | -0.012 | 0.032 | 0.72 |
| Peptococcus | rs36121075 | A | G | 0.128 | -0.141 | 0.031 | 6.99E-06 | -0.001 | 0.024 | 0.96 |
| Peptococcus | rs413827 | G | A | 0.213 | 0.110 | 0.024 | 3.30E-06 | -0.005 | 0.019 | 0.79 |
| Peptococcus | rs5770862 | T | C | 0.067 | 0.162 | 0.036 | 3.22E-06 | -0.002 | 0.023 | 0.92 |
| Peptococcus | rs6918730 | G | A | 0.097 | 0.135 | 0.029 | 1.15E-06 | 0.012 | 0.024 | 0.63 |
| Peptococcus | rs7033353 | T | G | 0.420 | 0.090 | 0.019 | 2.22E-06 | 0.021 | 0.015 | 0.16 |
| Peptococcus | rs72850165 | T | C | 0.108 | -0.134 | 0.030 | 5.74E-06 | -0.014 | 0.026 | 0.60 |
| Peptococcus | rs74592222 | G | A | 0.117 | 0.138 | 0.030 | 8.55E-06 | -0.035 | 0.025 | 0.18 |
| Peptococcus | rs75754569 | C | G | 0.110 | 0.181 | 0.032 | 1.10E-08 | -0.027 | 0.027 | 0.32 |
| Peptococcus | rs7766680 | G | C | 0.249 | 0.098 | 0.021 | 3.51E-06 | 0.017 | 0.017 | 0.30 |
| Peptococcus | rs77681628 | C | T | 0.059 | 0.200 | 0.039 | 2.69E-07 | -0.031 | 0.032 | 0.33 |
| Senegalimassilia | rs10036909 | C | T | 0.070 | 0.186 | 0.040 | 8.05E-06 | 0.035 | 0.030 | 0.23 |
| Senegalimassilia | rs11787826 | C | A | 0.428 | 0.081 | 0.017 | 2.63E-06 | -0.003 | 0.014 | 0.86 |
| Senegalimassilia | rs1990708 | A | C | 0.126 | -0.110 | 0.025 | 8.91E-06 | 0.043 | 0.021 | 0.03 |
| Senegalimassilia | rs2017373 | C | T | 0.374 | 0.078 | 0.018 | 9.50E-06 | 0.010 | 0.015 | 0.50 |
| Senegalimassilia | rs7225245 | G | A | 0.458 | 0.079 | 0.017 | 4.18E-06 | 0.007 | 0.015 | 0.64 |
| Hungatella | rs10044993 | C | A | 0.117 | 0.140 | 0.032 | 8.07E-06 | 0.025 | 0.020 | 0.22 |
| Hungatella | rs13128780 | T | C | 0.145 | -0.150 | 0.031 | 1.75E-06 | 0.004 | 0.021 | 0.87 |
| Hungatella | rs13249325 | T | G | 0.367 | -0.100 | 0.023 | 9.69E-06 | 0.003 | 0.015 | 0.84 |
| Hungatella | rs17092615 | G | A | 0.124 | 0.152 | 0.034 | 7.38E-06 | -0.023 | 0.023 | 0.32 |
| Hungatella | rs72759041 | G | T | 0.235 | -0.126 | 0.028 | 3.86E-06 | 0.006 | 0.018 | 0.75 |
| Howardella | rs10048062 | C | T | 0.107 | -0.147 | 0.034 | 8.59E-06 | -0.019 | 0.022 | 0.41 |
| Howardella | rs12452946 | A | G | 0.501 | -0.106 | 0.023 | 3.80E-06 | -0.032 | 0.014 | 0.02 |
| Howardella | rs1484873 | A | G | 0.069 | -0.228 | 0.046 | 2.56E-06 | -0.037 | 0.024 | 0.12 |
| Howardella | rs17167098 | G | A | 0.113 | -0.169 | 0.035 | 1.12E-06 | 0.001 | 0.023 | 0.98 |
| Howardella | rs2154047 | C | A | 0.068 | -0.193 | 0.042 | 9.97E-06 | -0.040 | 0.027 | 0.14 |
| Howardella | rs36081916 | T | C | 0.111 | -0.181 | 0.040 | 4.70E-06 | 0.008 | 0.026 | 0.77 |
| Howardella | rs3791893 | A | G | 0.123 | 0.147 | 0.034 | 9.50E-06 | 0.028 | 0.021 | 0.18 |
| Howardella | rs609430 | T | G | 0.339 | -0.112 | 0.024 | 3.34E-06 | -0.031 | 0.015 | 0.04 |
| Howardella | rs61771805 | A | T | 0.187 | -0.137 | 0.030 | 4.03E-06 | -0.003 | 0.019 | 0.88 |
| Howardella | rs672217 | G | A | 0.141 | 0.164 | 0.035 | 3.52E-06 | 0.051 | 0.021 | 0.02 |
| Howardella | rs901099 | T | G | 0.312 | -0.127 | 0.025 | 6.53E-07 | -0.030 | 0.020 | 0.14 |
| Subdoligranulum | rs10065321 | T | C | 0.368 | -0.051 | 0.011 | 2.10E-06 | 0.024 | 0.014 | 0.09 |
| Subdoligranulum | rs10497836 | C | T | 0.280 | -0.052 | 0.012 | 8.38E-06 | -0.008 | 0.016 | 0.61 |
| Subdoligranulum | rs1667315 | G | A | 0.437 | 0.049 | 0.011 | 6.72E-06 | 0.004 | 0.014 | 0.77 |
| Subdoligranulum | rs16962433 | A | T | 0.108 | 0.086 | 0.019 | 7.65E-06 | -0.021 | 0.028 | 0.46 |
| Subdoligranulum | rs2114677 | C | T | 0.056 | -0.104 | 0.023 | 2.72E-06 | -0.026 | 0.030 | 0.40 |
| Subdoligranulum | rs2171249 | C | T | 0.074 | 0.107 | 0.023 | 4.51E-06 | 0.061 | 0.031 | 0.05 |
| Subdoligranulum | rs35940633 | G | A | 0.363 | -0.051 | 0.011 | 4.22E-06 | -0.019 | 0.015 | 0.19 |
| Subdoligranulum | rs3761728 | T | G | 0.243 | -0.054 | 0.012 | 3.87E-06 | 0.000 | 0.016 | 0.99 |
| Subdoligranulum | rs4347804 | A | G | 0.042 | 0.166 | 0.036 | 2.18E-06 | -0.031 | 0.031 | 0.33 |
| Subdoligranulum | rs6555306 | T | C | 0.122 | -0.074 | 0.016 | 2.81E-06 | 0.030 | 0.020 | 0.14 |
| Subdoligranulum | rs75158211 | T | C | 0.111 | -0.072 | 0.016 | 7.52E-06 | 0.016 | 0.021 | 0.46 |
| Subdoligranulum | rs76528319 | G | T | 0.040 | -0.143 | 0.031 | 7.41E-06 | 0.018 | 0.029 | 0.55 |
| Subdoligranulum | rs76664262 | T | A | 0.093 | 0.083 | 0.019 | 4.87E-06 | -0.015 | 0.025 | 0.54 |
| Coprococcus2 | rs10070053 | A | G | 0.408 | 0.059 | 0.014 | 7.65E-06 | -0.014 | 0.016 | 0.38 |
| Coprococcus2 | rs12634070 | T | C | 0.193 | 0.074 | 0.016 | 9.95E-06 | -0.002 | 0.017 | 0.91 |
| Coprococcus2 | rs2482516 | C | T | 0.226 | 0.075 | 0.016 | 4.72E-06 | -0.021 | 0.018 | 0.23 |
| Coprococcus2 | rs35890118 | A | G | 0.317 | -0.067 | 0.015 | 8.26E-06 | -0.009 | 0.016 | 0.58 |
| Coprococcus2 | rs59936925 | A | T | 0.100 | 0.117 | 0.023 | 9.38E-07 | 0.003 | 0.027 | 0.91 |
| Coprococcus2 | rs61823518 | A | C | 0.095 | -0.096 | 0.022 | 6.68E-06 | -0.020 | 0.023 | 0.39 |
| Coprococcus2 | rs6677933 | C | T | 0.242 | -0.080 | 0.016 | 1.19E-06 | 0.006 | 0.019 | 0.75 |
| Coprococcus2 | rs72680320 | T | C | 0.414 | -0.065 | 0.014 | 2.27E-06 | 0.013 | 0.016 | 0.41 |
| Coprococcus2 | rs9426473 | A | G | 0.279 | 0.073 | 0.016 | 6.31E-06 | -0.006 | 0.017 | 0.72 |
| Clostridiuminnocuumgroup | rs10074000 | T | C | 0.414 | -0.103 | 0.023 | 7.00E-06 | 0.017 | 0.016 | 0.28 |
| Clostridiuminnocuumgroup | rs10506058 | A | G | 0.397 | 0.100 | 0.022 | 8.92E-06 | 0.008 | 0.014 | 0.58 |
| Clostridiuminnocuumgroup | rs1942371 | G | A | 0.130 | -0.158 | 0.034 | 4.06E-06 | -0.013 | 0.024 | 0.57 |
| Clostridiuminnocuumgroup | rs1948423 | T | A | 0.308 | -0.109 | 0.023 | 3.49E-06 | 0.002 | 0.015 | 0.87 |
| Clostridiuminnocuumgroup | rs40656 | C | T | 0.189 | 0.143 | 0.031 | 8.62E-06 | 0.020 | 0.021 | 0.35 |
| Clostridiuminnocuumgroup | rs4869133 | G | A | 0.089 | -0.181 | 0.041 | 7.24E-06 | 0.022 | 0.026 | 0.39 |
| Clostridiuminnocuumgroup | rs61267978 | T | C | 0.124 | 0.147 | 0.032 | 5.59E-06 | -0.012 | 0.022 | 0.57 |
| Clostridiuminnocuumgroup | rs6577484 | G | A | 0.148 | 0.160 | 0.036 | 8.41E-06 | -0.019 | 0.025 | 0.44 |
| Clostridiuminnocuumgroup | rs6890185 | T | C | 0.311 | 0.113 | 0.023 | 1.12E-06 | 0.012 | 0.015 | 0.43 |
| Clostridiuminnocuumgroup | rs71564433 | T | A | 0.179 | -0.127 | 0.027 | 7.80E-06 | -0.052 | 0.018 | 0.00 |
| Clostridiuminnocuumgroup | rs77845139 | A | G | 0.295 | -0.115 | 0.026 | 8.41E-06 | 0.022 | 0.020 | 0.26 |
| Butyrivibrio | rs1007475 | G | T | 0.294 | 0.118 | 0.026 | 7.92E-06 | 0.013 | 0.016 | 0.42 |
| Butyrivibrio | rs11761679 | T | C | 0.156 | 0.155 | 0.032 | 2.20E-06 | 0.018 | 0.021 | 0.38 |
| Butyrivibrio | rs142855850 | A | G | 0.101 | 0.205 | 0.046 | 6.86E-06 | -0.007 | 0.026 | 0.78 |
| Butyrivibrio | rs16934069 | T | C | 0.199 | -0.134 | 0.030 | 8.86E-06 | -0.003 | 0.017 | 0.87 |
| Butyrivibrio | rs16941336 | C | T | 0.264 | 0.127 | 0.027 | 1.53E-06 | -0.002 | 0.018 | 0.92 |
| Butyrivibrio | rs17163238 | G | A | 0.200 | 0.141 | 0.031 | 5.51E-06 | -0.002 | 0.019 | 0.90 |
| Butyrivibrio | rs4537857 | T | C | 0.292 | -0.125 | 0.026 | 1.80E-06 | -0.018 | 0.016 | 0.25 |
| Butyrivibrio | rs486484 | A | G | 0.471 | -0.108 | 0.024 | 6.61E-06 | 0.009 | 0.014 | 0.52 |
| Butyrivibrio | rs4928024 | A | G | 0.098 | -0.175 | 0.039 | 8.19E-06 | -0.015 | 0.020 | 0.44 |
| Butyrivibrio | rs72723662 | C | T | 0.100 | 0.224 | 0.045 | 7.86E-07 | -0.033 | 0.027 | 0.23 |
| Butyrivibrio | rs7412979 | C | G | 0.088 | 0.187 | 0.039 | 1.71E-06 | 0.000 | 0.026 | 0.99 |
| Butyrivibrio | rs74622183 | A | G | 0.088 | -0.201 | 0.043 | 2.46E-06 | 0.018 | 0.026 | 0.50 |
| Butyrivibrio | rs77356209 | T | C | 0.048 | 0.217 | 0.048 | 6.66E-06 | 0.008 | 0.025 | 0.75 |
| Butyrivibrio | rs7752361 | A | G | 0.504 | -0.119 | 0.024 | 7.69E-07 | -0.005 | 0.014 | 0.73 |
| Butyrivibrio | rs7763512 | G | A | 0.383 | 0.120 | 0.025 | 3.11E-06 | 0.022 | 0.015 | 0.14 |
| Butyrivibrio | rs9349693 | A | G | 0.311 | 0.118 | 0.026 | 5.55E-06 | 0.034 | 0.015 | 0.03 |
| Butyricicoccus | rs10084203 | A | G | 0.242 | 0.055 | 0.012 | 8.59E-06 | -0.023 | 0.017 | 0.16 |
| Butyricicoccus | rs12034718 | A | G | 0.165 | 0.070 | 0.016 | 9.58E-06 | -0.025 | 0.021 | 0.22 |
| Butyricicoccus | rs12585793 | T | C | 0.053 | -0.262 | 0.056 | 5.79E-06 | -0.003 | 0.034 | 0.93 |
| Butyricicoccus | rs2017189 | G | T | 0.422 | -0.051 | 0.011 | 3.87E-06 | -0.011 | 0.015 | 0.48 |
| Butyricicoccus | rs4962426 | G | T | 0.198 | 0.061 | 0.014 | 7.38E-06 | -0.001 | 0.017 | 0.95 |
| Butyricicoccus | rs56221232 | T | C | 0.108 | 0.083 | 0.017 | 7.62E-07 | -0.014 | 0.023 | 0.53 |
| Butyricicoccus | rs62478070 | T | G | 0.044 | 0.224 | 0.049 | 5.94E-06 | -0.006 | 0.038 | 0.87 |
| Butyricicoccus | rs7322368 | T | C | 0.085 | 0.082 | 0.018 | 5.52E-06 | -0.021 | 0.024 | 0.38 |
| Butyricicoccus | rs75238760 | T | A | 0.194 | 0.062 | 0.014 | 6.80E-06 | -0.048 | 0.019 | 0.01 |
| CandidatusSoleaferrea | rs10090365 | A | G | 0.559 | -0.083 | 0.018 | 4.17E-06 | 0.018 | 0.014 | 0.20 |
| CandidatusSoleaferrea | rs10108780 | A | G | 0.267 | -0.093 | 0.020 | 3.64E-06 | -0.019 | 0.017 | 0.28 |
| CandidatusSoleaferrea | rs10809135 | T | C | 0.491 | 0.083 | 0.018 | 5.47E-06 | -0.010 | 0.014 | 0.48 |
| CandidatusSoleaferrea | rs11153159 | G | C | 0.129 | -0.128 | 0.029 | 4.42E-06 | -0.022 | 0.023 | 0.32 |
| CandidatusSoleaferrea | rs2193878 | T | A | 0.049 | 0.228 | 0.051 | 9.46E-06 | 0.035 | 0.028 | 0.22 |
| CandidatusSoleaferrea | rs36155147 | C | T | 0.362 | 0.105 | 0.024 | 5.41E-06 | 0.006 | 0.036 | 0.87 |
| CandidatusSoleaferrea | rs4294381 | T | C | 0.180 | 0.112 | 0.023 | 1.37E-06 | -0.014 | 0.019 | 0.45 |
| CandidatusSoleaferrea | rs4678258 | T | C | 0.276 | 0.099 | 0.022 | 5.53E-06 | -0.007 | 0.018 | 0.69 |
| CandidatusSoleaferrea | rs6489992 | A | G | 0.385 | -0.084 | 0.019 | 7.89E-06 | -0.008 | 0.015 | 0.59 |
| CandidatusSoleaferrea | rs6494306 | A | G | 0.197 | -0.097 | 0.021 | 5.80E-06 | -0.011 | 0.016 | 0.48 |
| CandidatusSoleaferrea | rs7400877 | T | C | 0.251 | -0.095 | 0.021 | 9.29E-06 | -0.009 | 0.016 | 0.57 |
| CandidatusSoleaferrea | rs830149 | C | G | 0.059 | 0.185 | 0.040 | 9.58E-06 | 0.009 | 0.031 | 0.78 |
| CandidatusSoleaferrea | rs9973954 | A | G | 0.306 | 0.089 | 0.020 | 5.95E-06 | -0.016 | 0.017 | 0.32 |
| LachnospiraceaeFCS020group | rs10093861 | G | A | 0.498 | -0.057 | 0.012 | 3.06E-06 | -0.017 | 0.015 | 0.26 |
| LachnospiraceaeFCS020group | rs113859143 | G | C | 0.085 | -0.109 | 0.024 | 2.55E-06 | 0.015 | 0.032 | 0.63 |
| LachnospiraceaeFCS020group | rs12078956 | C | G | 0.121 | 0.106 | 0.022 | 2.15E-06 | 0.008 | 0.027 | 0.76 |
| LachnospiraceaeFCS020group | rs1254846 | G | A | 0.092 | 0.106 | 0.023 | 5.60E-06 | -0.028 | 0.028 | 0.32 |
| LachnospiraceaeFCS020group | rs1363769 | T | C | 0.033 | -0.201 | 0.045 | 1.58E-06 | 0.030 | 0.026 | 0.26 |
| LachnospiraceaeFCS020group | rs2322265 | C | T | 0.256 | -0.067 | 0.014 | 5.21E-06 | -0.021 | 0.018 | 0.25 |
| LachnospiraceaeFCS020group | rs2862811 | T | C | 0.454 | 0.056 | 0.012 | 3.92E-06 | -0.008 | 0.014 | 0.56 |
| LachnospiraceaeFCS020group | rs35035870 | T | C | 0.036 | -0.191 | 0.041 | 2.62E-06 | 0.018 | 0.037 | 0.63 |
| LachnospiraceaeFCS020group | rs369444 | C | G | 0.064 | 0.125 | 0.026 | 3.15E-06 | 0.017 | 0.025 | 0.49 |
| LachnospiraceaeFCS020group | rs3999074 | G | T | 0.478 | -0.055 | 0.012 | 6.55E-06 | -0.018 | 0.014 | 0.21 |
| LachnospiraceaeFCS020group | rs4452603 | T | G | 0.286 | 0.060 | 0.014 | 8.98E-06 | 0.014 | 0.016 | 0.37 |
| LachnospiraceaeFCS020group | rs7249113 | G | A | 0.281 | 0.068 | 0.013 | 3.72E-07 | -0.004 | 0.015 | 0.79 |
| LachnospiraceaeFCS020group | rs72793667 | A | G | 0.084 | -0.117 | 0.025 | 1.63E-06 | 0.016 | 0.030 | 0.60 |
| LachnospiraceaeFCS020group | rs9308097 | A | G | 0.452 | 0.055 | 0.012 | 7.47E-06 | 0.000 | 0.014 | 0.99 |
| LachnospiraceaeFCS020group | rs9788306 | C | T | 0.284 | -0.063 | 0.013 | 1.39E-06 | 0.023 | 0.015 | 0.13 |
| Odoribacter | rs10093869 | A | G | 0.376 | -0.058 | 0.013 | 3.67E-06 | -0.015 | 0.015 | 0.33 |
| Odoribacter | rs10423795 | C | T | 0.407 | 0.055 | 0.012 | 6.58E-06 | -0.007 | 0.015 | 0.63 |
| Odoribacter | rs16918425 | A | T | 0.075 | 0.100 | 0.022 | 8.85E-06 | -0.010 | 0.026 | 0.72 |
| Odoribacter | rs28417404 | A | G | 0.169 | -0.073 | 0.016 | 3.68E-06 | -0.056 | 0.023 | 0.01 |
| Odoribacter | rs4793970 | A | G | 0.301 | -0.058 | 0.013 | 6.03E-06 | -0.009 | 0.016 | 0.58 |
| Odoribacter | rs6856150 | G | A | 0.118 | 0.088 | 0.019 | 6.06E-06 | 0.022 | 0.022 | 0.32 |
| Odoribacter | rs74553962 | T | G | 0.063 | 0.121 | 0.026 | 9.49E-06 | 0.044 | 0.032 | 0.16 |
| Odoribacter | rs77779484 | G | A | 0.059 | -0.133 | 0.027 | 6.56E-07 | -0.034 | 0.034 | 0.33 |
| Enterorhabdus | rs10098492 | T | C | 0.091 | 0.132 | 0.029 | 6.41E-06 | -0.003 | 0.025 | 0.91 |
| Enterorhabdus | rs114731706 | T | G | 0.084 | 0.182 | 0.038 | 2.17E-06 | 0.002 | 0.036 | 0.95 |
| Enterorhabdus | rs2051957 | C | T | 0.235 | 0.084 | 0.019 | 8.90E-06 | 0.040 | 0.016 | 0.01 |
| Enterorhabdus | rs3017103 | A | G | 0.190 | 0.098 | 0.021 | 2.94E-06 | 0.008 | 0.017 | 0.63 |
| Enterorhabdus | rs73331712 | T | C | 0.043 | 0.262 | 0.055 | 4.85E-06 | 0.037 | 0.030 | 0.22 |
| Enterorhabdus | rs77655283 | G | A | 0.069 | 0.133 | 0.030 | 5.88E-06 | -0.001 | 0.027 | 0.96 |
| Enterorhabdus | rs7923280 | A | T | 0.395 | 0.086 | 0.017 | 5.24E-07 | 0.015 | 0.015 | 0.34 |
| Coprococcus1 | rs1010560 | C | A | 0.296 | 0.058 | 0.012 | 1.96E-06 | 0.013 | 0.017 | 0.46 |
| Coprococcus1 | rs12794898 | G | T | 0.094 | 0.090 | 0.020 | 4.92E-06 | 0.005 | 0.028 | 0.85 |
| Coprococcus1 | rs12886051 | G | C | 0.343 | -0.052 | 0.012 | 8.01E-06 | 0.002 | 0.016 | 0.90 |
| Coprococcus1 | rs1519491 | T | C | 0.409 | 0.050 | 0.011 | 8.95E-06 | -0.025 | 0.015 | 0.10 |
| Coprococcus1 | rs1576241 | A | G | 0.439 | -0.051 | 0.011 | 3.33E-06 | 0.005 | 0.014 | 0.71 |
| Coprococcus1 | rs1762123 | C | T | 0.072 | -0.089 | 0.020 | 8.01E-06 | 0.007 | 0.025 | 0.77 |
| Coprococcus1 | rs2907920 | A | G | 0.290 | 0.056 | 0.013 | 7.65E-06 | -0.020 | 0.016 | 0.22 |
| Coprococcus1 | rs4277593 | G | A | 0.483 | -0.059 | 0.011 | 1.14E-07 | -0.021 | 0.014 | 0.14 |
| Coprococcus1 | rs56405618 | A | G | 0.111 | -0.090 | 0.019 | 1.57E-06 | 0.002 | 0.023 | 0.94 |
| Coprococcus1 | rs73031725 | T | C | 0.061 | 0.168 | 0.036 | 1.98E-06 | -0.028 | 0.041 | 0.49 |
| Coprococcus1 | rs73167075 | T | C | 0.240 | 0.057 | 0.013 | 8.57E-06 | -0.010 | 0.017 | 0.56 |
| Coprococcus1 | rs74101919 | T | C | 0.187 | -0.072 | 0.014 | 1.03E-06 | -0.011 | 0.018 | 0.56 |
| Coprococcus1 | rs946513 | C | T | 0.055 | 0.206 | 0.046 | 8.62E-06 | 0.038 | 0.035 | 0.27 |
| Eubacteriumruminantiumgroup | rs10131724 | A | C | 0.064 | -0.200 | 0.041 | 2.39E-06 | 0.022 | 0.032 | 0.49 |
| Eubacteriumruminantiumgroup | rs10923018 | G | A | 0.472 | 0.073 | 0.016 | 6.80E-06 | -0.023 | 0.015 | 0.12 |
| Eubacteriumruminantiumgroup | rs112375806 | T | A | 0.107 | 0.143 | 0.029 | 5.82E-06 | 0.035 | 0.021 | 0.10 |
| Eubacteriumruminantiumgroup | rs11637981 | G | T | 0.484 | -0.073 | 0.016 | 5.44E-06 | 0.013 | 0.014 | 0.37 |
| Eubacteriumruminantiumgroup | rs13025464 | T | C | 0.383 | -0.074 | 0.016 | 6.97E-06 | 0.022 | 0.015 | 0.12 |
| Eubacteriumruminantiumgroup | rs139749 | C | T | 0.342 | -0.085 | 0.017 | 8.59E-07 | -0.013 | 0.015 | 0.39 |
| Eubacteriumruminantiumgroup | rs16891896 | G | A | 0.059 | -0.175 | 0.039 | 2.38E-06 | -0.031 | 0.027 | 0.25 |
| Eubacteriumruminantiumgroup | rs17519472 | C | T | 0.154 | 0.108 | 0.023 | 4.70E-06 | -0.033 | 0.020 | 0.10 |
| Eubacteriumruminantiumgroup | rs209813 | G | A | 0.166 | -0.103 | 0.024 | 9.23E-06 | 0.014 | 0.023 | 0.54 |
| Eubacteriumruminantiumgroup | rs2116427 | A | G | 0.257 | 0.091 | 0.018 | 4.67E-07 | 0.017 | 0.016 | 0.29 |
| Eubacteriumruminantiumgroup | rs2229917 | A | G | 0.067 | 0.154 | 0.032 | 2.16E-06 | 0.022 | 0.034 | 0.51 |
| Eubacteriumruminantiumgroup | rs2418654 | C | T | 0.478 | -0.075 | 0.017 | 6.17E-06 | -0.007 | 0.015 | 0.63 |
| Eubacteriumruminantiumgroup | rs2817174 | C | T | 0.429 | -0.073 | 0.016 | 7.87E-06 | -0.013 | 0.014 | 0.39 |
| Eubacteriumruminantiumgroup | rs57340348 | T | C | 0.215 | -0.098 | 0.021 | 4.93E-06 | 0.024 | 0.019 | 0.22 |
| Eubacteriumruminantiumgroup | rs606117 | A | G | 0.332 | 0.083 | 0.018 | 4.82E-06 | 0.006 | 0.017 | 0.72 |
| Eubacteriumruminantiumgroup | rs6676699 | G | T | 0.222 | -0.089 | 0.020 | 6.38E-06 | -0.008 | 0.017 | 0.63 |
| Eubacteriumruminantiumgroup | rs7000472 | A | G | 0.362 | -0.076 | 0.017 | 4.07E-06 | 0.004 | 0.015 | 0.79 |
| Eubacteriumruminantiumgroup | rs72836424 | C | T | 0.095 | -0.140 | 0.030 | 2.62E-06 | -0.029 | 0.024 | 0.23 |
| Eubacteriumruminantiumgroup | rs73139629 | A | C | 0.132 | -0.115 | 0.025 | 5.36E-06 | 0.013 | 0.024 | 0.60 |
| Dialister | rs10138457 | T | C | 0.072 | -0.113 | 0.026 | 7.88E-06 | 0.039 | 0.030 | 0.20 |
| Dialister | rs10938938 | G | A | 0.194 | -0.077 | 0.017 | 7.37E-06 | 0.023 | 0.020 | 0.25 |
| Dialister | rs11071887 | T | C | 0.263 | 0.066 | 0.015 | 5.91E-06 | -0.010 | 0.016 | 0.51 |
| Dialister | rs11166701 | G | A | 0.516 | -0.066 | 0.013 | 5.51E-07 | 0.030 | 0.015 | 0.05 |
| Dialister | rs2314294 | T | C | 0.118 | 0.087 | 0.019 | 8.08E-06 | -0.023 | 0.022 | 0.30 |
| Dialister | rs2435610 | A | C | 0.260 | 0.065 | 0.014 | 5.93E-06 | 0.004 | 0.016 | 0.80 |
| Dialister | rs4747450 | C | A | 0.266 | 0.067 | 0.015 | 5.84E-06 | 0.012 | 0.017 | 0.48 |
| Dialister | rs4753063 | G | A | 0.441 | -0.060 | 0.013 | 4.86E-06 | 0.000 | 0.014 | 0.99 |
| Dialister | rs517089 | T | A | 0.169 | 0.076 | 0.017 | 5.14E-06 | -0.039 | 0.017 | 0.02 |
| Dialister | rs75416973 | A | G | 0.226 | 0.073 | 0.016 | 9.46E-06 | 0.011 | 0.020 | 0.58 |
| Dialister | rs764177 | C | A | 0.338 | -0.060 | 0.014 | 9.61E-06 | -0.018 | 0.015 | 0.24 |
| Dialister | rs76680460 | G | A | 0.048 | -0.161 | 0.036 | 8.19E-06 | -0.013 | 0.036 | 0.71 |
| Eubacteriumxylanophilumgroup | rs10140184 | A | C | 0.413 | 0.058 | 0.013 | 4.96E-06 | -0.007 | 0.015 | 0.63 |
| Eubacteriumxylanophilumgroup | rs10917203 | A | C | 0.335 | 0.061 | 0.013 | 3.15E-06 | -0.016 | 0.015 | 0.29 |
| Eubacteriumxylanophilumgroup | rs112176119 | C | T | 0.065 | -0.113 | 0.025 | 3.33E-06 | 0.000 | 0.029 | 0.99 |
| Eubacteriumxylanophilumgroup | rs12980122 | C | G | 0.085 | -0.108 | 0.024 | 5.02E-06 | -0.004 | 0.029 | 0.90 |
| Eubacteriumxylanophilumgroup | rs13239072 | G | A | 0.275 | 0.069 | 0.014 | 1.82E-06 | 0.020 | 0.017 | 0.23 |
| Eubacteriumxylanophilumgroup | rs17830032 | G | A | 0.054 | -0.161 | 0.031 | 2.39E-07 | 0.027 | 0.034 | 0.43 |
| Eubacteriumxylanophilumgroup | rs1999224 | G | T | 0.089 | -0.095 | 0.020 | 3.75E-06 | -0.004 | 0.024 | 0.88 |
| Eubacteriumxylanophilumgroup | rs2012708 | A | G | 0.364 | 0.057 | 0.013 | 6.53E-06 | -0.016 | 0.015 | 0.28 |
| Eubacteriumxylanophilumgroup | rs2213117 | T | G | 0.161 | 0.088 | 0.019 | 4.21E-06 | -0.011 | 0.021 | 0.58 |
| Eubacteriumxylanophilumgroup | rs75586835 | A | G | 0.067 | -0.114 | 0.026 | 9.39E-06 | -0.060 | 0.032 | 0.06 |
| Eubacteriumxylanophilumgroup | rs79582700 | C | G | 0.107 | -0.095 | 0.020 | 2.41E-06 | -0.043 | 0.022 | 0.05 |
| Eubacteriumfissicatenagroup | rs10147907 | T | G | 0.069 | 0.172 | 0.040 | 8.27E-06 | 0.013 | 0.023 | 0.57 |
| Eubacteriumfissicatenagroup | rs11818408 | G | A | 0.477 | 0.106 | 0.024 | 8.20E-06 | 0.015 | 0.014 | 0.30 |
| Eubacteriumfissicatenagroup | rs11876297 | T | C | 0.216 | 0.131 | 0.028 | 2.67E-06 | -0.030 | 0.019 | 0.11 |
| Eubacteriumfissicatenagroup | rs151257695 | A | G | 0.081 | 0.210 | 0.045 | 3.10E-06 | -0.017 | 0.028 | 0.54 |
| Eubacteriumfissicatenagroup | rs1768152 | T | C | 0.137 | 0.139 | 0.032 | 8.70E-06 | 0.014 | 0.020 | 0.49 |
| Eubacteriumfissicatenagroup | rs2733072 | G | A | 0.465 | 0.110 | 0.023 | 1.49E-06 | 0.019 | 0.014 | 0.18 |
| Eubacteriumfissicatenagroup | rs3771393 | C | T | 0.284 | 0.131 | 0.027 | 7.38E-07 | 0.000 | 0.017 | 0.98 |
| Eubacteriumfissicatenagroup | rs6934739 | A | G | 0.295 | 0.111 | 0.025 | 9.75E-06 | -0.013 | 0.015 | 0.40 |
| Eubacteriumfissicatenagroup | rs7104872 | G | A | 0.178 | 0.139 | 0.029 | 2.73E-06 | 0.013 | 0.020 | 0.53 |
| Anaerotruncus | rs10150232 | A | G | 0.254 | 0.057 | 0.012 | 6.68E-06 | 0.003 | 0.016 | 0.88 |
| Anaerotruncus | rs11018566 | A | G | 0.052 | -0.156 | 0.037 | 6.14E-06 | -0.009 | 0.029 | 0.76 |
| Anaerotruncus | rs115414803 | A | C | 0.059 | -0.144 | 0.032 | 6.83E-06 | 0.020 | 0.036 | 0.58 |
| Anaerotruncus | rs12056802 | C | G | 0.083 | 0.077 | 0.018 | 6.13E-06 | -0.010 | 0.021 | 0.62 |
| Anaerotruncus | rs1272208 | G | T | 0.280 | -0.061 | 0.013 | 4.28E-06 | 0.011 | 0.018 | 0.54 |
| Anaerotruncus | rs1431492 | C | T | 0.183 | -0.065 | 0.015 | 7.36E-06 | -0.035 | 0.018 | 0.05 |
| Anaerotruncus | rs17734739 | T | C | 0.190 | 0.066 | 0.015 | 7.43E-06 | -0.014 | 0.021 | 0.50 |
| Anaerotruncus | rs2704155 | T | A | 0.965 | -0.106 | 0.023 | 5.42E-06 | -0.191 | 0.786 | 0.81 |
| Anaerotruncus | rs34449434 | A | C | 0.456 | -0.050 | 0.011 | 9.85E-06 | 0.012 | 0.017 | 0.50 |
| Anaerotruncus | rs4669806 | G | T | 0.250 | 0.058 | 0.012 | 2.42E-06 | 0.012 | 0.016 | 0.46 |
| Anaerotruncus | rs6494922 | A | G | 0.073 | 0.090 | 0.020 | 6.62E-06 | -0.020 | 0.025 | 0.44 |
| Anaerotruncus | rs6563550 | T | C | 0.079 | 0.088 | 0.018 | 2.35E-07 | -0.017 | 0.021 | 0.44 |
| Anaerotruncus | rs7155595 | C | A | 0.309 | 0.054 | 0.012 | 7.55E-06 | 0.026 | 0.016 | 0.10 |
| Anaerotruncus | rs8005030 | C | T | 0.302 | 0.055 | 0.012 | 2.28E-06 | 0.001 | 0.017 | 0.98 |
| Anaerotruncus | rs9347879 | T | C | 0.460 | 0.051 | 0.011 | 4.22E-06 | -0.017 | 0.014 | 0.24 |
| ErysipelotrichaceaeUCG003 | rs10164067 | T | G | 0.075 | -0.103 | 0.021 | 1.13E-06 | -0.051 | 0.027 | 0.06 |
| ErysipelotrichaceaeUCG003 | rs11666127 | A | G | 0.171 | -0.072 | 0.016 | 7.90E-06 | -0.007 | 0.021 | 0.75 |
| ErysipelotrichaceaeUCG003 | rs11994308 | C | T | 0.070 | 0.115 | 0.024 | 1.33E-06 | 0.000 | 0.030 | 0.99 |
| ErysipelotrichaceaeUCG003 | rs12251396 | A | G | 0.194 | -0.071 | 0.016 | 9.52E-06 | -0.030 | 0.021 | 0.15 |
| ErysipelotrichaceaeUCG003 | rs17798136 | G | A | 0.062 | 0.159 | 0.035 | 3.24E-06 | -0.034 | 0.037 | 0.37 |
| ErysipelotrichaceaeUCG003 | rs28568391 | A | G | 0.449 | -0.058 | 0.012 | 6.42E-07 | -0.005 | 0.015 | 0.76 |
| ErysipelotrichaceaeUCG003 | rs4758231 | G | T | 0.350 | -0.055 | 0.012 | 6.55E-06 | 0.027 | 0.015 | 0.08 |
| ErysipelotrichaceaeUCG003 | rs59068084 | T | G | 0.422 | 0.056 | 0.012 | 3.12E-06 | 0.030 | 0.015 | 0.04 |
| ErysipelotrichaceaeUCG003 | rs59104037 | A | G | 0.082 | -0.095 | 0.020 | 4.48E-06 | 0.052 | 0.024 | 0.03 |
| ErysipelotrichaceaeUCG003 | rs62403464 | T | C | 0.164 | -0.073 | 0.016 | 3.44E-06 | 0.044 | 0.018 | 0.02 |
| ErysipelotrichaceaeUCG003 | rs6875357 | C | T | 0.047 | 0.166 | 0.035 | 6.70E-06 | -0.019 | 0.032 | 0.54 |
| ErysipelotrichaceaeUCG003 | rs73074432 | C | T | 0.166 | 0.072 | 0.016 | 9.99E-06 | -0.006 | 0.022 | 0.80 |
| ErysipelotrichaceaeUCG003 | rs74988980 | G | A | 0.044 | -0.133 | 0.035 | 8.64E-06 | 0.016 | 0.025 | 0.51 |
| ErysipelotrichaceaeUCG003 | rs75949021 | T | C | 0.046 | -0.170 | 0.037 | 3.58E-06 | -0.029 | 0.034 | 0.40 |
| ErysipelotrichaceaeUCG003 | rs76502207 | T | C | 0.091 | 0.145 | 0.029 | 6.41E-07 | -0.057 | 0.034 | 0.10 |
| ErysipelotrichaceaeUCG003 | rs79396538 | C | G | 0.103 | 0.085 | 0.019 | 8.63E-06 | 0.021 | 0.026 | 0.42 |
| ErysipelotrichaceaeUCG003 | rs8053479 | A | G | 0.141 | -0.084 | 0.019 | 5.83E-06 | -0.009 | 0.024 | 0.71 |
| Ruminococcus1 | rs10167839 | A | G | 0.344 | 0.052 | 0.012 | 8.09E-06 | 0.013 | 0.015 | 0.40 |
| Ruminococcus1 | rs10995816 | C | G | 0.112 | -0.076 | 0.017 | 8.38E-06 | -0.034 | 0.022 | 0.12 |
| Ruminococcus1 | rs11783695 | G | T | 0.151 | -0.073 | 0.016 | 4.73E-06 | -0.037 | 0.021 | 0.07 |
| Ruminococcus1 | rs17781867 | C | T | 0.092 | 0.100 | 0.021 | 1.96E-06 | 0.000 | 0.029 | 0.99 |
| Ruminococcus1 | rs3000856 | T | A | 0.975 | -0.071 | 0.016 | 9.28E-06 | -0.019 | 0.020 | 0.36 |
| Ruminococcus1 | rs3819978 | C | T | 0.059 | -0.115 | 0.026 | 8.74E-06 | 0.018 | 0.031 | 0.57 |
| Ruminococcus1 | rs4849717 | T | A | 0.053 | 0.133 | 0.030 | 8.83E-06 | -0.043 | 0.034 | 0.20 |
| Ruminococcus1 | rs6105066 | T | C | 0.250 | -0.061 | 0.013 | 5.06E-06 | 0.027 | 0.017 | 0.12 |
| Ruminococcus1 | rs6493760 | C | T | 0.397 | 0.054 | 0.012 | 3.38E-06 | -0.013 | 0.015 | 0.38 |
| Ruminococcus1 | rs7117576 | A | G | 0.089 | 0.083 | 0.017 | 6.48E-07 | 0.017 | 0.024 | 0.49 |
| Ruminococcus1 | rs7583465 | C | T | 0.377 | 0.053 | 0.011 | 2.56E-06 | 0.004 | 0.014 | 0.80 |
| Ruminococcus1 | rs78572139 | G | A | 0.071 | 0.125 | 0.028 | 5.23E-06 | -0.029 | 0.031 | 0.35 |
| Ruminococcus1 | rs78613526 | G | A | 0.043 | 0.167 | 0.037 | 5.11E-06 | 0.024 | 0.032 | 0.45 |
| Methanobrevibacter | rs10202904 | T | G | 0.467 | -0.113 | 0.024 | 3.09E-06 | -0.014 | 0.015 | 0.36 |
| Methanobrevibacter | rs11018665 | A | T | 0.341 | 0.113 | 0.025 | 7.03E-06 | 0.008 | 0.016 | 0.62 |
| Methanobrevibacter | rs1334944 | T | C | 0.285 | 0.115 | 0.026 | 7.61E-06 | 0.015 | 0.015 | 0.31 |
| Methanobrevibacter | rs4779844 | G | C | 0.324 | 0.110 | 0.025 | 9.28E-06 | -0.038 | 0.015 | 0.01 |
| Methanobrevibacter | rs4802933 | A | G | 0.178 | -0.136 | 0.031 | 9.74E-06 | -0.003 | 0.018 | 0.86 |
| Methanobrevibacter | rs6776814 | T | C | 0.090 | -0.189 | 0.042 | 8.05E-06 | 0.055 | 0.030 | 0.06 |
| Methanobrevibacter | rs76029318 | T | C | 0.065 | 0.223 | 0.045 | 1.08E-06 | -0.025 | 0.026 | 0.34 |
| Methanobrevibacter | rs894996 | C | A | 0.072 | 0.214 | 0.046 | 3.82E-06 | -0.005 | 0.028 | 0.87 |
| Eubacteriumcoprostanoligenesgroup | rs1020520 | T | G | 0.204 | -0.059 | 0.013 | 8.89E-06 | 0.004 | 0.017 | 0.80 |
| Eubacteriumcoprostanoligenesgroup | rs10444197 | A | G | 0.368 | -0.051 | 0.011 | 5.98E-06 | 0.030 | 0.016 | 0.05 |
| Eubacteriumcoprostanoligenesgroup | rs11052069 | T | C | 0.434 | 0.048 | 0.011 | 9.38E-06 | -0.044 | 0.014 | 0.00 |
| Eubacteriumcoprostanoligenesgroup | rs115325767 | G | C | 0.093 | 0.078 | 0.017 | 2.62E-06 | 0.024 | 0.030 | 0.42 |
| Eubacteriumcoprostanoligenesgroup | rs11720857 | C | T | 0.177 | 0.063 | 0.014 | 9.26E-06 | -0.021 | 0.018 | 0.25 |
| Eubacteriumcoprostanoligenesgroup | rs12906958 | C | T | 0.302 | -0.053 | 0.012 | 4.35E-06 | -0.014 | 0.015 | 0.36 |
| Eubacteriumcoprostanoligenesgroup | rs17159861 | C | T | 0.133 | 0.096 | 0.017 | 1.04E-08 | -0.009 | 0.022 | 0.69 |
| Eubacteriumcoprostanoligenesgroup | rs2644213 | G | A | 0.237 | 0.054 | 0.012 | 9.86E-06 | 0.025 | 0.016 | 0.11 |
| Eubacteriumcoprostanoligenesgroup | rs4076415 | T | G | 0.401 | 0.052 | 0.011 | 1.99E-06 | 0.023 | 0.015 | 0.13 |
| Eubacteriumcoprostanoligenesgroup | rs4717831 | A | T | 0.119 | 0.079 | 0.017 | 9.18E-06 | -0.018 | 0.027 | 0.49 |
| Eubacteriumcoprostanoligenesgroup | rs62024432 | C | T | 0.125 | -0.077 | 0.017 | 7.50E-06 | 0.004 | 0.023 | 0.86 |
| Eubacteriumcoprostanoligenesgroup | rs6762473 | C | A | 0.357 | 0.052 | 0.011 | 4.26E-06 | -0.022 | 0.017 | 0.19 |
| Eubacteriumcoprostanoligenesgroup | rs76898927 | G | A | 0.048 | 0.123 | 0.027 | 4.79E-06 | -0.022 | 0.037 | 0.55 |
| Eubacteriumcoprostanoligenesgroup | rs79895140 | T | C | 0.217 | -0.064 | 0.014 | 8.62E-06 | -0.007 | 0.021 | 0.75 |
| Eubacteriumcoprostanoligenesgroup | rs9648214 | T | C | 0.118 | -0.083 | 0.016 | 2.52E-07 | 0.004 | 0.021 | 0.85 |
| Eubacteriumrectalegroup | rs10248854 | C | A | 0.360 | -0.053 | 0.011 | 4.21E-06 | -0.019 | 0.015 | 0.23 |
| Eubacteriumrectalegroup | rs10797540 | A | G | 0.434 | 0.050 | 0.011 | 3.53E-06 | 0.004 | 0.014 | 0.76 |
| Eubacteriumrectalegroup | rs10892089 | C | G | 0.197 | -0.064 | 0.014 | 6.22E-06 | 0.035 | 0.019 | 0.06 |
| Eubacteriumrectalegroup | rs117151453 | G | C | 0.051 | -0.113 | 0.024 | 1.84E-06 | 0.057 | 0.031 | 0.07 |
| Eubacteriumrectalegroup | rs143694765 | T | C | 0.094 | 0.087 | 0.020 | 9.75E-06 | 0.011 | 0.027 | 0.68 |
| Eubacteriumrectalegroup | rs16960159 | C | G | 0.048 | -0.157 | 0.034 | 4.10E-06 | -0.017 | 0.033 | 0.60 |
| Eubacteriumrectalegroup | rs2884897 | A | G | 0.053 | -0.129 | 0.029 | 6.44E-06 | 0.024 | 0.033 | 0.47 |
| Eubacteriumrectalegroup | rs314726 | T | C | 0.457 | 0.053 | 0.011 | 1.38E-06 | 0.005 | 0.015 | 0.75 |
| Eubacteriumrectalegroup | rs35398954 | A | G | 0.121 | -0.090 | 0.017 | 5.40E-07 | -0.004 | 0.022 | 0.88 |
| Eubacteriumrectalegroup | rs3980709 | A | T | 0.161 | -0.062 | 0.014 | 6.86E-06 | -0.024 | 0.018 | 0.18 |
| Eubacteriumrectalegroup | rs59427698 | A | G | 0.203 | -0.058 | 0.013 | 5.37E-06 | 0.002 | 0.019 | 0.92 |
| Eubacteriumrectalegroup | rs62547233 | A | G | 0.307 | 0.054 | 0.012 | 9.90E-06 | -0.005 | 0.016 | 0.74 |
| Eubacteriumnodatumgroup | rs10263623 | C | T | 0.104 | 0.193 | 0.044 | 8.91E-06 | -0.013 | 0.028 | 0.65 |
| Eubacteriumnodatumgroup | rs10458299 | T | C | 0.081 | -0.188 | 0.042 | 8.37E-06 | 0.001 | 0.029 | 0.99 |
| Eubacteriumnodatumgroup | rs11006576 | A | G | 0.513 | -0.110 | 0.025 | 7.99E-06 | 0.002 | 0.014 | 0.88 |
| Eubacteriumnodatumgroup | rs113893692 | C | T | 0.098 | -0.185 | 0.040 | 5.76E-06 | -0.015 | 0.021 | 0.47 |
| Eubacteriumnodatumgroup | rs34297067 | A | G | 0.139 | -0.187 | 0.034 | 6.60E-08 | 0.034 | 0.022 | 0.12 |
| Eubacteriumnodatumgroup | rs61841040 | G | T | 0.166 | 0.161 | 0.034 | 3.56E-06 | -0.014 | 0.018 | 0.44 |
| Eubacteriumnodatumgroup | rs6818880 | A | G | 0.455 | -0.110 | 0.025 | 7.83E-06 | -0.018 | 0.015 | 0.22 |
| Eubacteriumnodatumgroup | rs77910827 | C | T | 0.103 | 0.202 | 0.041 | 9.05E-07 | -0.003 | 0.025 | 0.89 |
| Eubacteriumnodatumgroup | rs7827125 | C | T | 0.282 | 0.122 | 0.027 | 7.17E-06 | -0.018 | 0.016 | 0.24 |
| Eubacteriumnodatumgroup | rs7880204 | T | C | 0.248 | -0.125 | 0.028 | 6.84E-06 | -0.026 | 0.016 | 0.11 |
| Eubacteriumnodatumgroup | rs9425984 | T | C | 0.239 | -0.130 | 0.029 | 7.21E-06 | 0.001 | 0.019 | 0.97 |
| RuminococcaceaeUCG011 | rs10274562 | C | T | 0.352 | 0.111 | 0.024 | 6.50E-06 | 0.006 | 0.015 | 0.69 |
| RuminococcaceaeUCG011 | rs12636310 | G | A | 0.218 | 0.133 | 0.028 | 2.81E-06 | 0.028 | 0.017 | 0.10 |
| RuminococcaceaeUCG011 | rs12724320 | C | T | 0.275 | -0.121 | 0.025 | 1.52E-06 | 0.003 | 0.016 | 0.86 |
| RuminococcaceaeUCG011 | rs1416041 | A | C | 0.134 | -0.182 | 0.034 | 7.04E-08 | 0.016 | 0.020 | 0.41 |
| RuminococcaceaeUCG011 | rs2729556 | C | T | 0.499 | -0.109 | 0.023 | 3.19E-06 | -0.008 | 0.015 | 0.57 |
| RuminococcaceaeUCG011 | rs4490371 | T | C | 0.331 | -0.112 | 0.025 | 7.75E-06 | 0.010 | 0.016 | 0.50 |
| RuminococcaceaeUCG011 | rs79113084 | C | T | 0.155 | -0.152 | 0.032 | 2.06E-06 | -0.004 | 0.022 | 0.84 |
| RuminococcaceaeUCG011 | rs9729514 | A | G | 0.102 | 0.185 | 0.039 | 2.37E-06 | 0.001 | 0.025 | 0.98 |
| Romboutsia | rs10279978 | A | G | 0.348 | -0.062 | 0.013 | 1.17E-06 | 0.021 | 0.015 | 0.17 |
| Romboutsia | rs11221428 | T | C | 0.155 | -0.073 | 0.016 | 6.49E-06 | 0.011 | 0.020 | 0.58 |
| Romboutsia | rs114398731 | G | C | 0.036 | -0.131 | 0.029 | 7.96E-06 | 0.025 | 0.035 | 0.48 |
| Romboutsia | rs16843578 | C | T | 0.083 | -0.088 | 0.020 | 5.08E-06 | -0.010 | 0.022 | 0.66 |
| Romboutsia | rs28603357 | T | C | 0.064 | -0.215 | 0.047 | 8.52E-06 | 0.024 | 0.040 | 0.56 |
| Romboutsia | rs34302036 | A | G | 0.415 | 0.055 | 0.012 | 5.88E-06 | -0.003 | 0.014 | 0.83 |
| Romboutsia | rs61841503 | G | A | 0.137 | 0.093 | 0.017 | 4.00E-08 | 0.004 | 0.021 | 0.85 |
| Romboutsia | rs62504452 | A | G | 0.166 | -0.071 | 0.016 | 4.66E-06 | -0.030 | 0.019 | 0.12 |
| Romboutsia | rs7109293 | A | G | 0.092 | 0.092 | 0.021 | 6.98E-06 | -0.051 | 0.022 | 0.02 |
| Romboutsia | rs75200530 | T | G | 0.047 | -0.191 | 0.042 | 5.07E-06 | 0.098 | 0.034 | 0.00 |
| Romboutsia | rs75987356 | G | A | 0.069 | -0.130 | 0.028 | 6.71E-06 | -0.011 | 0.032 | 0.74 |
| Romboutsia | rs77702691 | A | G | 0.102 | -0.094 | 0.021 | 7.37E-06 | -0.032 | 0.026 | 0.23 |
| Romboutsia | rs9389266 | T | G | 0.169 | 0.072 | 0.016 | 9.38E-06 | -0.019 | 0.019 | 0.31 |
| Romboutsia | rs9567264 | C | T | 0.320 | 0.058 | 0.013 | 5.76E-06 | 0.003 | 0.015 | 0.83 |
| Lachnoclostridium | rs1031599 | G | T | 0.085 | -0.079 | 0.018 | 6.31E-06 | -0.015 | 0.022 | 0.50 |
| Lachnoclostridium | rs12566975 | T | C | 0.493 | -0.047 | 0.011 | 9.57E-06 | -0.003 | 0.014 | 0.81 |
| Lachnoclostridium | rs1528479 | G | A | 0.370 | -0.050 | 0.011 | 9.64E-06 | 0.003 | 0.015 | 0.83 |
| Lachnoclostridium | rs1997204 | T | C | 0.053 | -0.108 | 0.024 | 5.97E-06 | -0.060 | 0.035 | 0.08 |
| Lachnoclostridium | rs2385421 | A | G | 0.104 | 0.075 | 0.018 | 7.14E-06 | -0.012 | 0.024 | 0.62 |
| Lachnoclostridium | rs3821998 | C | A | 0.089 | -0.086 | 0.019 | 6.72E-06 | -0.022 | 0.024 | 0.35 |
| Lachnoclostridium | rs4738679 | G | A | 0.365 | -0.052 | 0.011 | 4.42E-06 | -0.005 | 0.015 | 0.77 |
| Lachnoclostridium | rs6112314 | A | C | 0.397 | -0.056 | 0.011 | 2.43E-07 | -0.021 | 0.015 | 0.14 |
| Lachnoclostridium | rs615997 | T | C | 0.499 | 0.051 | 0.011 | 2.03E-06 | -0.004 | 0.015 | 0.77 |
| Lachnoclostridium | rs62285313 | A | G | 0.086 | 0.086 | 0.018 | 1.58E-06 | 0.025 | 0.026 | 0.34 |
| Lachnoclostridium | rs72829893 | G | T | 0.066 | 0.117 | 0.027 | 5.58E-06 | -0.039 | 0.031 | 0.22 |
| Lachnoclostridium | rs78068103 | A | G | 0.092 | 0.089 | 0.019 | 3.67E-06 | 0.012 | 0.023 | 0.60 |
| Lachnoclostridium | rs789029 | C | T | 0.189 | -0.064 | 0.014 | 3.75E-06 | -0.018 | 0.018 | 0.31 |
| Olsenella | rs1035588 | A | G | 0.383 | -0.108 | 0.024 | 4.86E-06 | 0.007 | 0.015 | 0.65 |
| Olsenella | rs17148768 | G | A | 0.212 | 0.140 | 0.030 | 2.20E-06 | -0.037 | 0.020 | 0.06 |
| Olsenella | rs2759329 | G | A | 0.340 | -0.111 | 0.024 | 3.43E-06 | 0.017 | 0.015 | 0.26 |
| Olsenella | rs35225860 | A | G | 0.057 | -0.224 | 0.048 | 3.87E-06 | -0.032 | 0.034 | 0.34 |
| Olsenella | rs6046522 | C | T | 0.286 | 0.123 | 0.027 | 4.48E-06 | -0.012 | 0.021 | 0.55 |
| Olsenella | rs61090148 | A | G | 0.423 | -0.105 | 0.023 | 6.44E-06 | -0.002 | 0.014 | 0.89 |
| Olsenella | rs62112538 | C | T | 0.081 | -0.199 | 0.041 | 1.19E-06 | -0.068 | 0.028 | 0.02 |
| Olsenella | rs72691585 | C | A | 0.069 | -0.249 | 0.052 | 2.95E-06 | 0.022 | 0.031 | 0.48 |
| Olsenella | rs7540303 | C | T | 0.422 | 0.108 | 0.024 | 5.32E-06 | -0.019 | 0.014 | 0.18 |
| Olsenella | rs8066522 | G | A | 0.347 | -0.107 | 0.024 | 9.70E-06 | 0.022 | 0.015 | 0.14 |
| Olsenella | rs9460691 | C | A | 0.217 | 0.120 | 0.027 | 7.28E-06 | 0.026 | 0.017 | 0.13 |
| Slackia | rs10409783 | A | G | 0.283 | 0.095 | 0.021 | 7.70E-06 | -0.002 | 0.017 | 0.89 |
| Slackia | rs112764253 | T | A | 0.041 | 0.195 | 0.041 | 3.40E-06 | -0.062 | 0.036 | 0.09 |
| Slackia | rs12440440 | A | G | 0.309 | 0.090 | 0.019 | 2.63E-06 | -0.009 | 0.015 | 0.56 |
| Slackia | rs13339230 | C | G | 0.089 | 0.147 | 0.033 | 7.42E-06 | -0.011 | 0.028 | 0.71 |
| Slackia | rs16894137 | C | T | 0.143 | -0.123 | 0.026 | 2.71E-06 | 0.007 | 0.020 | 0.72 |
| Slackia | rs35156985 | T | C | 0.064 | -0.156 | 0.035 | 8.06E-06 | -0.008 | 0.026 | 0.75 |
| Slackia | rs4492265 | A | G | 0.310 | -0.091 | 0.019 | 2.41E-06 | 0.011 | 0.015 | 0.45 |
| Slackia | rs58767323 | G | C | 0.190 | -0.103 | 0.023 | 4.60E-06 | -0.006 | 0.017 | 0.72 |
| Slackia | rs8901 | C | T | 0.371 | 0.093 | 0.019 | 6.07E-07 | -0.006 | 0.015 | 0.66 |
| LachnospiraceaeUCG010 | rs10414815 | T | C | 0.119 | 0.105 | 0.023 | 4.24E-06 | 0.019 | 0.027 | 0.47 |
| LachnospiraceaeUCG010 | rs11192447 | A | G | 0.075 | 0.127 | 0.024 | 4.69E-07 | -0.023 | 0.027 | 0.39 |
| LachnospiraceaeUCG010 | rs12346653 | C | T | 0.274 | 0.066 | 0.014 | 2.70E-06 | 0.023 | 0.016 | 0.16 |
| LachnospiraceaeUCG010 | rs17730011 | G | A | 0.219 | -0.070 | 0.016 | 7.85E-06 | 0.011 | 0.018 | 0.54 |
| LachnospiraceaeUCG010 | rs2153460 | A | T | 0.184 | -0.068 | 0.016 | 9.17E-06 | 0.038 | 0.019 | 0.05 |
| LachnospiraceaeUCG010 | rs2833528 | C | T | 0.361 | -0.056 | 0.013 | 9.92E-06 | 0.006 | 0.015 | 0.67 |
| LachnospiraceaeUCG010 | rs336138 | G | T | 0.163 | 0.078 | 0.017 | 7.48E-06 | 0.010 | 0.021 | 0.62 |
| LachnospiraceaeUCG010 | rs4576377 | A | C | 0.384 | -0.057 | 0.013 | 7.63E-06 | -0.018 | 0.014 | 0.22 |
| LachnospiraceaeUCG010 | rs72761829 | A | T | 0.072 | 0.112 | 0.024 | 2.58E-06 | -0.021 | 0.029 | 0.48 |
| LachnospiraceaeUCG010 | rs72894957 | G | A | 0.048 | 0.222 | 0.049 | 5.68E-06 | -0.022 | 0.037 | 0.56 |
| LachnospiraceaeUCG010 | rs74315802 | G | T | 0.121 | 0.087 | 0.018 | 3.19E-06 | -0.009 | 0.023 | 0.71 |
| LachnospiraceaeUCG010 | rs9981767 | A | C | 0.316 | 0.066 | 0.013 | 9.96E-07 | 0.014 | 0.016 | 0.38 |
| Lactococcus | rs10417872 | T | G | 0.258 | 0.118 | 0.025 | 1.29E-06 | 0.010 | 0.016 | 0.51 |
| Lactococcus | rs123059 | T | C | 0.222 | -0.137 | 0.027 | 1.27E-06 | 0.004 | 0.017 | 0.82 |
| Lactococcus | rs12621813 | G | A | 0.282 | 0.108 | 0.024 | 6.61E-06 | -0.010 | 0.015 | 0.49 |
| Lactococcus | rs17168302 | G | A | 0.066 | 0.192 | 0.042 | 6.29E-06 | 0.016 | 0.023 | 0.48 |
| Lactococcus | rs2293361 | C | T | 0.065 | -0.199 | 0.043 | 1.40E-06 | -0.011 | 0.025 | 0.66 |
| Lactococcus | rs34757988 | G | C | 0.326 | 0.122 | 0.023 | 8.95E-08 | -0.002 | 0.015 | 0.88 |
| Lactococcus | rs4766997 | C | T | 0.296 | 0.115 | 0.024 | 2.06E-06 | 0.029 | 0.015 | 0.05 |
| Lactococcus | rs55910161 | C | T | 0.135 | 0.146 | 0.031 | 2.36E-06 | 0.025 | 0.021 | 0.22 |
| Lactococcus | rs6674304 | C | T | 0.069 | 0.201 | 0.044 | 6.18E-06 | 0.036 | 0.028 | 0.19 |
| Lactococcus | rs757872 | G | C | 0.206 | 0.141 | 0.028 | 4.37E-07 | 0.018 | 0.017 | 0.29 |
| Lactococcus | rs7992246 | T | C | 0.347 | 0.104 | 0.023 | 4.45E-06 | 0.040 | 0.016 | 0.02 |
| Fusicatenibacter | rs10439674 | A | G | 0.202 | -0.057 | 0.013 | 7.68E-06 | -0.018 | 0.017 | 0.27 |
| Fusicatenibacter | rs167879 | C | T | 0.167 | -0.066 | 0.015 | 5.87E-06 | -0.029 | 0.021 | 0.17 |
| Fusicatenibacter | rs1864685 | A | C | 0.465 | -0.049 | 0.011 | 4.96E-06 | 0.011 | 0.015 | 0.47 |
| Fusicatenibacter | rs2025938 | G | A | 0.077 | -0.097 | 0.021 | 2.99E-06 | -0.012 | 0.029 | 0.68 |
| Fusicatenibacter | rs206581 | A | G | 0.209 | -0.057 | 0.013 | 8.96E-06 | -0.010 | 0.017 | 0.56 |
| Fusicatenibacter | rs2132128 | G | A | 0.165 | -0.077 | 0.016 | 1.08E-06 | 0.003 | 0.023 | 0.89 |
| Fusicatenibacter | rs3303 | T | C | 0.077 | -0.095 | 0.020 | 3.94E-06 | -0.003 | 0.025 | 0.92 |
| Fusicatenibacter | rs4378146 | A | C | 0.242 | -0.062 | 0.013 | 7.20E-07 | -0.008 | 0.017 | 0.62 |
| Fusicatenibacter | rs60254196 | A | G | 0.541 | -0.049 | 0.011 | 5.47E-06 | -0.001 | 0.015 | 0.95 |
| Fusicatenibacter | rs62187631 | T | C | 0.140 | -0.071 | 0.016 | 4.55E-06 | -0.002 | 0.021 | 0.92 |
| Fusicatenibacter | rs62353480 | A | G | 0.171 | -0.070 | 0.015 | 1.57E-06 | 0.015 | 0.021 | 0.48 |
| Fusicatenibacter | rs6515626 | G | A | 0.062 | 0.142 | 0.031 | 7.29E-06 | -0.024 | 0.032 | 0.46 |
| Fusicatenibacter | rs704418 | T | C | 0.186 | 0.074 | 0.015 | 7.77E-07 | -0.002 | 0.023 | 0.95 |
| Fusicatenibacter | rs73103914 | A | G | 0.178 | -0.060 | 0.013 | 8.30E-06 | 0.025 | 0.018 | 0.15 |
| Fusicatenibacter | rs792108 | T | C | 0.354 | -0.051 | 0.011 | 8.50E-06 | 0.009 | 0.015 | 0.53 |
| Fusicatenibacter | rs8028026 | A | G | 0.092 | -0.079 | 0.018 | 8.06E-06 | -0.032 | 0.026 | 0.21 |
| Fusicatenibacter | rs8063430 | T | C | 0.060 | -0.104 | 0.022 | 4.93E-06 | -0.021 | 0.027 | 0.45 |
| Fusicatenibacter | rs9905659 | G | A | 0.188 | -0.062 | 0.014 | 7.31E-06 | -0.007 | 0.018 | 0.68 |
| ChristensenellaceaeR.7group | rs10461257 | A | G | 0.285 | -0.055 | 0.012 | 6.51E-06 | -0.004 | 0.016 | 0.80 |
| ChristensenellaceaeR.7group | rs17081797 | A | G | 0.069 | -0.090 | 0.020 | 3.34E-06 | -0.036 | 0.024 | 0.14 |
| ChristensenellaceaeR.7group | rs60954665 | T | G | 0.510 | 0.050 | 0.011 | 7.13E-06 | 0.007 | 0.018 | 0.69 |
| ChristensenellaceaeR.7group | rs62132810 | A | G | 0.108 | -0.083 | 0.018 | 5.67E-06 | 0.002 | 0.025 | 0.95 |
| ChristensenellaceaeR.7group | rs62190261 | A | C | 0.091 | 0.096 | 0.021 | 8.74E-06 | 0.041 | 0.030 | 0.17 |
| ChristensenellaceaeR.7group | rs62467127 | C | T | 0.045 | 0.114 | 0.025 | 3.25E-06 | -0.027 | 0.028 | 0.32 |
| ChristensenellaceaeR.7group | rs73952017 | C | T | 0.088 | -0.086 | 0.019 | 8.46E-06 | -0.002 | 0.025 | 0.95 |
| ChristensenellaceaeR.7group | rs78521377 | C | T | 0.053 | 0.125 | 0.027 | 5.61E-06 | -0.001 | 0.032 | 0.97 |
| ChristensenellaceaeR.7group | rs79150079 | C | A | 0.072 | 0.122 | 0.027 | 9.42E-06 | -0.056 | 0.039 | 0.15 |
| ChristensenellaceaeR.7group | rs892686 | A | G | 0.451 | 0.051 | 0.011 | 3.97E-06 | 0.006 | 0.015 | 0.67 |
| Adlercreutzia | rs1046175 | C | G | 0.126 | 0.113 | 0.026 | 6.36E-06 | 0.014 | 0.025 | 0.57 |
| Adlercreutzia | rs11604400 | C | T | 0.115 | -0.103 | 0.023 | 9.74E-06 | -0.004 | 0.025 | 0.87 |
| Adlercreutzia | rs12522517 | A | T | 0.127 | -0.105 | 0.023 | 4.41E-06 | -0.024 | 0.021 | 0.26 |
| Adlercreutzia | rs13231526 | C | A | 0.064 | 0.143 | 0.031 | 4.81E-06 | -0.043 | 0.029 | 0.13 |
| Adlercreutzia | rs2147798 | C | G | 0.196 | 0.092 | 0.019 | 1.40E-06 | 0.009 | 0.017 | 0.59 |
| Adlercreutzia | rs2717140 | C | T | 0.111 | -0.119 | 0.025 | 2.05E-06 | -0.017 | 0.022 | 0.44 |
| Adlercreutzia | rs55719207 | G | A | 0.418 | -0.070 | 0.016 | 9.61E-06 | 0.002 | 0.014 | 0.92 |
| Adlercreutzia | rs6664405 | T | C | 0.144 | -0.095 | 0.021 | 5.23E-06 | -0.005 | 0.022 | 0.81 |
| Adlercreutzia | rs7680684 | C | T | 0.325 | -0.083 | 0.017 | 9.77E-07 | -0.005 | 0.016 | 0.74 |
| Adlercreutzia | rs80078995 | A | T | 0.134 | -0.113 | 0.023 | 1.57E-06 | 0.002 | 0.020 | 0.92 |
| Adlercreutzia | rs9490822 | C | T | 0.447 | -0.073 | 0.016 | 2.54E-06 | 0.012 | 0.014 | 0.41 |
| Adlercreutzia | rs9915817 | T | C | 0.329 | 0.075 | 0.017 | 8.22E-06 | 0.028 | 0.015 | 0.07 |
| Oxalobacter | rs10464997 | G | A | 0.153 | 0.138 | 0.029 | 3.30E-06 | -0.012 | 0.018 | 0.50 |
| Oxalobacter | rs11108500 | A | G | 0.077 | -0.199 | 0.043 | 3.74E-06 | -0.005 | 0.031 | 0.86 |
| Oxalobacter | rs111966731 | T | C | 0.072 | 0.213 | 0.047 | 7.30E-06 | 0.005 | 0.030 | 0.87 |
| Oxalobacter | rs12002250 | A | C | 0.060 | 0.217 | 0.047 | 1.42E-06 | 0.033 | 0.035 | 0.36 |
| Oxalobacter | rs1569853 | T | C | 0.138 | -0.138 | 0.030 | 3.65E-06 | 0.032 | 0.022 | 0.15 |
| Oxalobacter | rs36057338 | G | T | 0.076 | 0.208 | 0.042 | 8.80E-07 | 0.002 | 0.030 | 0.95 |
| Oxalobacter | rs3862635 | C | T | 0.079 | -0.172 | 0.039 | 9.19E-06 | -0.026 | 0.031 | 0.40 |
| Oxalobacter | rs4428215 | G | A | 0.260 | 0.130 | 0.024 | 7.51E-08 | 0.011 | 0.016 | 0.52 |
| Oxalobacter | rs6000536 | C | T | 0.211 | -0.131 | 0.025 | 2.06E-07 | -0.005 | 0.020 | 0.81 |
| Oxalobacter | rs6071435 | T | A | 0.364 | -0.106 | 0.021 | 1.07E-06 | 0.009 | 0.016 | 0.57 |
| Oxalobacter | rs6993398 | G | A | 0.153 | 0.127 | 0.028 | 7.13E-06 | 0.056 | 0.019 | 0.00 |
| Oxalobacter | rs736744 | C | T | 0.416 | 0.118 | 0.021 | 2.57E-08 | 0.004 | 0.015 | 0.79 |
| RuminococcaceaeUCG003 | rs10490280 | C | T | 0.218 | -0.067 | 0.014 | 4.16E-06 | 0.000 | 0.018 | 0.99 |
| RuminococcaceaeUCG003 | rs11243416 | T | C | 0.101 | -0.093 | 0.019 | 1.67E-06 | -0.032 | 0.024 | 0.20 |
| RuminococcaceaeUCG003 | rs11613919 | G | T | 0.213 | 0.073 | 0.016 | 1.63E-06 | -0.003 | 0.020 | 0.89 |
| RuminococcaceaeUCG003 | rs139730 | G | C | 0.286 | -0.058 | 0.013 | 9.72E-06 | -0.010 | 0.016 | 0.51 |
| RuminococcaceaeUCG003 | rs16959793 | A | C | 0.280 | -0.063 | 0.013 | 2.22E-06 | 0.019 | 0.015 | 0.22 |
| RuminococcaceaeUCG003 | rs2523124 | T | C | 0.398 | -0.055 | 0.012 | 5.78E-06 | -0.015 | 0.015 | 0.30 |
| RuminococcaceaeUCG003 | rs3013089 | G | A | 0.385 | -0.055 | 0.012 | 4.38E-06 | 0.015 | 0.014 | 0.29 |
| RuminococcaceaeUCG003 | rs4452755 | A | C | 0.305 | -0.063 | 0.013 | 3.29E-06 | -0.007 | 0.017 | 0.71 |
| RuminococcaceaeUCG003 | rs4532474 | G | A | 0.149 | 0.077 | 0.017 | 4.82E-06 | 0.006 | 0.020 | 0.78 |
| RuminococcaceaeUCG003 | rs4629039 | T | A | 0.340 | 0.055 | 0.012 | 6.54E-06 | -0.014 | 0.015 | 0.33 |
| RuminococcaceaeUCG003 | rs646327 | G | A | 0.468 | 0.059 | 0.012 | 7.83E-07 | 0.018 | 0.016 | 0.25 |
| RuminococcaceaeUCG003 | rs6759615 | A | G | 0.070 | 0.103 | 0.020 | 7.86E-07 | -0.013 | 0.023 | 0.58 |
| RuminococcaceaeUCG003 | rs73341549 | T | C | 0.051 | -0.170 | 0.032 | 1.51E-07 | -0.024 | 0.029 | 0.40 |
| RuminococcaceaeUCG003 | rs78720113 | A | G | 0.070 | -0.115 | 0.025 | 7.59E-06 | -0.039 | 0.026 | 0.14 |
| RuminococcaceaeUCG014 | rs10495392 | C | T | 0.119 | -0.082 | 0.019 | 9.96E-06 | -0.044 | 0.024 | 0.07 |
| RuminococcaceaeUCG014 | rs10791168 | A | G | 0.184 | -0.066 | 0.015 | 9.76E-06 | 0.012 | 0.019 | 0.54 |
| RuminococcaceaeUCG014 | rs10941294 | C | T | 0.069 | -0.122 | 0.026 | 2.40E-06 | -0.015 | 0.026 | 0.55 |
| RuminococcaceaeUCG014 | rs115777838 | T | C | 0.054 | -0.188 | 0.039 | 4.62E-07 | -0.019 | 0.033 | 0.57 |
| RuminococcaceaeUCG014 | rs12638134 | T | G | 0.390 | 0.058 | 0.012 | 1.21E-06 | 0.007 | 0.014 | 0.64 |
| RuminococcaceaeUCG014 | rs17296933 | C | G | 0.127 | -0.083 | 0.019 | 7.34E-06 | 0.006 | 0.024 | 0.80 |
| RuminococcaceaeUCG014 | rs34402072 | C | T | 0.192 | -0.069 | 0.016 | 9.80E-06 | -0.011 | 0.019 | 0.57 |
| RuminococcaceaeUCG014 | rs439810 | G | C | 0.297 | -0.058 | 0.013 | 7.04E-06 | -0.008 | 0.016 | 0.61 |
| RuminococcaceaeUCG014 | rs56105232 | G | A | 0.073 | 0.139 | 0.030 | 2.91E-06 | 0.013 | 0.033 | 0.70 |
| RuminococcaceaeUCG014 | rs61898819 | A | T | 0.211 | 0.061 | 0.014 | 9.92E-06 | 0.006 | 0.018 | 0.75 |
| RuminococcaceaeUCG014 | rs62478832 | T | A | 0.288 | -0.058 | 0.013 | 6.04E-06 | 0.018 | 0.015 | 0.25 |
| RuminococcaceaeUCG014 | rs72809222 | T | C | 0.252 | 0.067 | 0.014 | 2.41E-06 | -0.009 | 0.017 | 0.61 |
| RuminococcaceaeUCG014 | rs73186226 | G | A | 0.088 | -0.099 | 0.022 | 6.72E-06 | -0.048 | 0.029 | 0.10 |
| RuminococcaceaeUCG014 | rs74060145 | C | G | 0.065 | -0.116 | 0.025 | 8.71E-06 | 0.017 | 0.032 | 0.59 |
| RuminococcaceaeUCG014 | rs77627087 | C | G | 0.186 | 0.068 | 0.015 | 7.43E-06 | 0.002 | 0.020 | 0.93 |
| RuminococcaceaeUCG014 | rs79640386 | T | A | 0.065 | -0.111 | 0.025 | 8.74E-06 | -0.051 | 0.024 | 0.03 |
| RuminococcaceaeUCG014 | rs853612 | A | G | 0.403 | -0.053 | 0.012 | 9.75E-06 | 0.007 | 0.015 | 0.62 |
| RuminococcaceaeUCG014 | rs995642 | C | T | 0.326 | 0.060 | 0.013 | 1.90E-06 | 0.028 | 0.015 | 0.07 |
| Eubacteriumhalliigroup | rs10501370 | C | T | 0.053 | -0.116 | 0.025 | 5.42E-06 | -0.010 | 0.036 | 0.79 |
| Eubacteriumhalliigroup | rs10798999 | C | T | 0.216 | 0.060 | 0.013 | 2.61E-06 | 0.002 | 0.017 | 0.89 |
| Eubacteriumhalliigroup | rs10808115 | A | C | 0.478 | -0.050 | 0.011 | 4.42E-06 | -0.014 | 0.014 | 0.33 |
| Eubacteriumhalliigroup | rs117748144 | T | C | 0.043 | -0.127 | 0.029 | 7.86E-06 | -0.017 | 0.033 | 0.61 |
| Eubacteriumhalliigroup | rs13116360 | T | C | 0.063 | 0.154 | 0.030 | 2.94E-07 | -0.023 | 0.034 | 0.50 |
| Eubacteriumhalliigroup | rs138531890 | A | G | 0.054 | 0.153 | 0.035 | 5.43E-06 | 0.009 | 0.036 | 0.79 |
| Eubacteriumhalliigroup | rs17074066 | T | C | 0.092 | -0.081 | 0.019 | 9.35E-06 | 0.005 | 0.024 | 0.84 |
| Eubacteriumhalliigroup | rs17474256 | G | A | 0.123 | 0.081 | 0.018 | 9.45E-06 | 0.014 | 0.024 | 0.55 |
| Eubacteriumhalliigroup | rs281379 | A | G | 0.460 | -0.050 | 0.011 | 9.33E-06 | 0.018 | 0.016 | 0.25 |
| Eubacteriumhalliigroup | rs28584818 | A | G | 0.069 | 0.126 | 0.027 | 4.43E-06 | 0.014 | 0.031 | 0.66 |
| Eubacteriumhalliigroup | rs60254196 | A | G | 0.541 | -0.052 | 0.011 | 2.70E-06 | -0.001 | 0.015 | 0.95 |
| Eubacteriumhalliigroup | rs630939 | C | T | 0.400 | -0.051 | 0.011 | 9.16E-06 | -0.005 | 0.015 | 0.76 |
| Eubacteriumhalliigroup | rs6550770 | T | C | 0.052 | -0.198 | 0.044 | 4.82E-06 | 0.028 | 0.036 | 0.44 |
| Eubacteriumhalliigroup | rs74018587 | C | T | 0.037 | 0.209 | 0.044 | 3.70E-06 | 0.034 | 0.030 | 0.25 |
| Eubacteriumhalliigroup | rs78056098 | G | T | 0.361 | -0.051 | 0.011 | 8.29E-06 | -0.016 | 0.015 | 0.26 |
| Eubacteriumhalliigroup | rs949971 | T | G | 0.384 | -0.054 | 0.012 | 3.29E-06 | 0.031 | 0.016 | 0.06 |
| Anaerostipes | rs10502061 | A | G | 0.094 | 0.084 | 0.019 | 7.94E-06 | -0.008 | 0.023 | 0.74 |
| Anaerostipes | rs13376554 | A | T | 0.031 | 0.197 | 0.046 | 7.72E-06 | -0.013 | 0.040 | 0.75 |
| Anaerostipes | rs2014785 | T | C | 0.389 | 0.052 | 0.011 | 4.68E-06 | 0.032 | 0.016 | 0.04 |
| Anaerostipes | rs2396460 | T | C | 0.439 | -0.051 | 0.011 | 2.91E-06 | 0.001 | 0.014 | 0.97 |
| Anaerostipes | rs2804244 | A | G | 0.455 | -0.053 | 0.011 | 2.04E-06 | -0.018 | 0.016 | 0.26 |
| Anaerostipes | rs3900776 | G | A | 0.071 | -0.110 | 0.024 | 2.75E-06 | 0.048 | 0.033 | 0.15 |
| Anaerostipes | rs60983350 | G | A | 0.435 | -0.054 | 0.012 | 4.42E-06 | -0.005 | 0.035 | 0.89 |
| Anaerostipes | rs62157625 | T | C | 0.113 | 0.089 | 0.019 | 1.45E-06 | -0.014 | 0.025 | 0.58 |
| Anaerostipes | rs62215703 | G | A | 0.220 | 0.064 | 0.014 | 1.98E-06 | 0.002 | 0.019 | 0.92 |
| Anaerostipes | rs6474958 | A | G | 0.403 | -0.050 | 0.011 | 6.74E-06 | 0.003 | 0.015 | 0.86 |
| Anaerostipes | rs6726833 | C | A | 0.094 | -0.088 | 0.019 | 3.32E-06 | 0.001 | 0.024 | 0.97 |
| Anaerostipes | rs6854026 | T | C | 0.512 | -0.051 | 0.011 | 3.20E-06 | -0.010 | 0.014 | 0.50 |
| Anaerostipes | rs7193624 | C | T | 0.149 | 0.075 | 0.015 | 5.35E-07 | -0.017 | 0.021 | 0.41 |
| Anaerostipes | rs7823228 | G | C | 0.216 | -0.062 | 0.014 | 5.84E-06 | 0.023 | 0.017 | 0.18 |
| Anaerostipes | rs78735375 | A | C | 0.053 | -0.137 | 0.031 | 5.33E-06 | -0.006 | 0.036 | 0.86 |
| Prevotella9 | rs10512344 | C | G | 0.033 | 0.247 | 0.054 | 3.19E-06 | -0.013 | 0.034 | 0.70 |
| Prevotella9 | rs111509883 | T | C | 0.085 | 0.171 | 0.035 | 1.24E-06 | -0.003 | 0.030 | 0.92 |
| Prevotella9 | rs11199734 | A | T | 0.218 | 0.077 | 0.017 | 7.00E-06 | 0.005 | 0.017 | 0.77 |
| Prevotella9 | rs11685699 | C | T | 0.083 | -0.141 | 0.030 | 2.03E-06 | -0.025 | 0.030 | 0.41 |
| Prevotella9 | rs117271932 | A | G | 0.047 | 0.208 | 0.044 | 2.82E-06 | 0.001 | 0.035 | 0.98 |
| Prevotella9 | rs12648235 | T | C | 0.157 | 0.079 | 0.018 | 7.39E-06 | -0.015 | 0.018 | 0.40 |
| Prevotella9 | rs1304512 | G | A | 0.219 | 0.076 | 0.017 | 5.29E-06 | 0.014 | 0.017 | 0.39 |
| Prevotella9 | rs16966465 | G | C | 0.235 | 0.074 | 0.017 | 9.33E-06 | 0.001 | 0.019 | 0.98 |
| Prevotella9 | rs2104588 | T | C | 0.119 | 0.106 | 0.024 | 8.13E-06 | -0.030 | 0.025 | 0.23 |
| Prevotella9 | rs2495052 | A | G | 0.179 | 0.084 | 0.019 | 8.97E-06 | 0.001 | 0.020 | 0.97 |
| Prevotella9 | rs2683313 | A | G | 0.308 | -0.072 | 0.015 | 1.69E-06 | -0.028 | 0.015 | 0.07 |
| Prevotella9 | rs4968431 | G | T | 0.364 | 0.064 | 0.014 | 8.58E-06 | 0.030 | 0.015 | 0.05 |
| Prevotella9 | rs7237249 | C | T | 0.135 | -0.082 | 0.018 | 8.93E-06 | -0.018 | 0.018 | 0.34 |
| Prevotella9 | rs72815774 | T | C | 0.071 | -0.176 | 0.039 | 8.78E-06 | 0.031 | 0.030 | 0.31 |
| Prevotella9 | rs746764 | T | C | 0.173 | -0.092 | 0.019 | 2.04E-06 | 0.017 | 0.020 | 0.39 |
| Prevotella9 | rs7976209 | T | C | 0.167 | -0.087 | 0.020 | 7.28E-06 | -0.034 | 0.022 | 0.11 |
| Prevotella9 | rs9428102 | A | G | 0.237 | -0.078 | 0.018 | 4.62E-06 | -0.005 | 0.019 | 0.80 |
| Prevotella9 | rs9613013 | G | A | 0.145 | 0.092 | 0.020 | 6.10E-06 | -0.021 | 0.019 | 0.27 |
| LachnospiraceaeUCG008 | rs10741777 | T | C | 0.261 | -0.097 | 0.019 | 7.69E-07 | -0.022 | 0.016 | 0.17 |
| LachnospiraceaeUCG008 | rs10793103 | C | T | 0.304 | 0.097 | 0.018 | 9.35E-08 | -0.004 | 0.015 | 0.79 |
| LachnospiraceaeUCG008 | rs10801803 | G | A | 0.162 | -0.117 | 0.024 | 1.40E-06 | -0.012 | 0.020 | 0.54 |
| LachnospiraceaeUCG008 | rs13024781 | T | C | 0.503 | -0.080 | 0.017 | 2.29E-06 | -0.023 | 0.014 | 0.11 |
| LachnospiraceaeUCG008 | rs57091572 | A | G | 0.140 | -0.110 | 0.024 | 2.86E-06 | 0.002 | 0.020 | 0.93 |
| LachnospiraceaeUCG008 | rs57254474 | G | A | 0.241 | 0.089 | 0.020 | 6.92E-06 | 0.012 | 0.018 | 0.52 |
| LachnospiraceaeUCG008 | rs61944774 | A | G | 0.058 | 0.180 | 0.039 | 6.34E-06 | 0.001 | 0.034 | 0.97 |
| LachnospiraceaeUCG008 | rs62277846 | C | T | 0.236 | 0.102 | 0.021 | 1.59E-06 | 0.011 | 0.018 | 0.56 |
| LachnospiraceaeUCG008 | rs67078837 | T | C | 0.458 | -0.085 | 0.017 | 7.68E-07 | 0.017 | 0.015 | 0.24 |
| LachnospiraceaeUCG008 | rs75356640 | G | A | 0.089 | 0.137 | 0.030 | 9.83E-06 | -0.017 | 0.028 | 0.53 |
| LachnospiraceaeUCG008 | rs955844 | A | C | 0.200 | 0.112 | 0.023 | 1.81E-06 | -0.009 | 0.019 | 0.64 |
| LachnospiraceaeUCG008 | rs9873555 | G | C | 0.157 | -0.121 | 0.023 | 2.41E-07 | 0.004 | 0.022 | 0.86 |
| Haemophilus | rs10781340 | G | A | 0.166 | 0.095 | 0.020 | 4.32E-06 | -0.020 | 0.022 | 0.35 |
| Haemophilus | rs111582866 | G | A | 0.101 | -0.124 | 0.026 | 1.27E-06 | 0.071 | 0.026 | 0.01 |
| Haemophilus | rs12191680 | C | G | 0.165 | 0.107 | 0.020 | 1.47E-07 | -0.014 | 0.020 | 0.49 |
| Haemophilus | rs12876183 | T | A | 0.275 | 0.075 | 0.017 | 9.62E-06 | 0.022 | 0.016 | 0.17 |
| Haemophilus | rs35509 | G | A | 0.079 | 0.128 | 0.027 | 2.01E-06 | -0.018 | 0.026 | 0.50 |
| Haemophilus | rs4822728 | T | C | 0.437 | 0.071 | 0.015 | 3.48E-06 | 0.022 | 0.014 | 0.13 |
| Haemophilus | rs56310940 | G | C | 0.083 | -0.108 | 0.025 | 7.23E-06 | -0.029 | 0.021 | 0.16 |
| Haemophilus | rs76022354 | C | T | 0.033 | 0.245 | 0.051 | 1.83E-06 | -0.045 | 0.034 | 0.19 |
| Haemophilus | rs78909003 | T | C | 0.050 | -0.246 | 0.050 | 1.67E-06 | 0.012 | 0.035 | 0.73 |
| Haemophilus | rs9328464 | T | C | 0.492 | 0.072 | 0.015 | 1.42E-06 | 0.030 | 0.014 | 0.04 |
| Haemophilus | rs9382510 | C | T | 0.246 | -0.094 | 0.017 | 7.12E-08 | -0.008 | 0.016 | 0.62 |
| Haemophilus | rs9574096 | A | T | 0.362 | -0.074 | 0.016 | 2.18E-06 | -0.016 | 0.016 | 0.34 |
| Haemophilus | rs9895850 | T | C | 0.050 | -0.193 | 0.042 | 2.14E-06 | -0.011 | 0.030 | 0.71 |
| Actinomyces | rs10787984 | G | C | 0.176 | 0.094 | 0.021 | 9.62E-06 | 0.021 | 0.019 | 0.27 |
| Actinomyces | rs2715439 | C | T | 0.454 | 0.075 | 0.016 | 6.27E-06 | 0.000 | 0.015 | 0.98 |
| Actinomyces | rs34583783 | G | T | 0.102 | 0.127 | 0.027 | 4.49E-06 | 0.033 | 0.025 | 0.19 |
| Actinomyces | rs35011108 | A | G | 0.064 | 0.233 | 0.051 | 6.34E-06 | 0.014 | 0.035 | 0.70 |
| Actinomyces | rs4073240 | G | A | 0.383 | 0.075 | 0.017 | 7.94E-06 | 0.020 | 0.015 | 0.16 |
| Actinomyces | rs4146653 | G | A | 0.197 | 0.099 | 0.021 | 4.50E-06 | 0.019 | 0.019 | 0.31 |
| Actinomyces | rs71315246 | A | G | 0.143 | -0.097 | 0.022 | 9.83E-06 | 0.037 | 0.020 | 0.07 |
| Actinomyces | rs7915461 | T | C | 0.058 | 0.188 | 0.040 | 5.92E-06 | 0.010 | 0.027 | 0.72 |
| Anaerofilum | rs10794359 | T | C | 0.487 | -0.095 | 0.020 | 2.23E-06 | 0.010 | 0.015 | 0.51 |
| Anaerofilum | rs1563175 | A | C | 0.444 | 0.092 | 0.020 | 5.54E-06 | -0.015 | 0.016 | 0.34 |
| Anaerofilum | rs17012738 | T | G | 0.408 | 0.090 | 0.020 | 7.24E-06 | -0.007 | 0.014 | 0.64 |
| Anaerofilum | rs17096874 | C | T | 0.181 | -0.126 | 0.027 | 2.86E-06 | -0.005 | 0.018 | 0.78 |
| Anaerofilum | rs17105491 | G | C | 0.060 | -0.193 | 0.041 | 1.57E-06 | 0.025 | 0.033 | 0.45 |
| Anaerofilum | rs356049 | G | A | 0.186 | 0.133 | 0.029 | 6.56E-06 | 0.023 | 0.022 | 0.31 |
| Anaerofilum | rs4244069 | G | A | 0.113 | -0.147 | 0.033 | 9.81E-06 | 0.012 | 0.024 | 0.60 |
| Anaerofilum | rs4506496 | G | A | 0.337 | 0.103 | 0.021 | 1.49E-06 | -0.006 | 0.017 | 0.75 |
| Anaerofilum | rs712981 | A | C | 0.419 | 0.101 | 0.020 | 6.83E-07 | -0.002 | 0.014 | 0.90 |
| Anaerofilum | rs79598899 | C | T | 0.083 | 0.183 | 0.036 | 3.75E-07 | -0.016 | 0.030 | 0.60 |
| Anaerofilum | rs816292 | T | C | 0.243 | -0.113 | 0.022 | 2.64E-07 | 0.007 | 0.016 | 0.65 |
| Anaerofilum | rs9299345 | T | C | 0.117 | -0.136 | 0.030 | 8.04E-06 | -0.004 | 0.020 | 0.85 |
| Intestinibacter | rs10805326 | G | A | 0.278 | 0.078 | 0.014 | 3.55E-08 | 0.015 | 0.017 | 0.38 |
| Intestinibacter | rs11109097 | C | T | 0.312 | 0.062 | 0.014 | 5.49E-06 | 0.023 | 0.015 | 0.12 |
| Intestinibacter | rs118030283 | G | A | 0.038 | -0.152 | 0.032 | 2.67E-06 | 0.007 | 0.035 | 0.85 |
| Intestinibacter | rs16938435 | T | C | 0.061 | -0.112 | 0.024 | 1.80E-06 | -0.010 | 0.023 | 0.65 |
| Intestinibacter | rs2098844 | C | T | 0.425 | -0.058 | 0.013 | 6.79E-06 | -0.022 | 0.015 | 0.13 |
| Intestinibacter | rs2702387 | A | G | 0.352 | 0.061 | 0.013 | 4.26E-06 | -0.027 | 0.015 | 0.06 |
| Intestinibacter | rs4327025 | G | A | 0.195 | -0.081 | 0.015 | 1.64E-07 | -0.002 | 0.017 | 0.91 |
| Intestinibacter | rs447950 | A | G | 0.299 | 0.063 | 0.014 | 5.64E-06 | -0.006 | 0.015 | 0.67 |
| Intestinibacter | rs478972 | T | C | 0.060 | -0.143 | 0.030 | 1.82E-06 | 0.010 | 0.034 | 0.77 |
| Intestinibacter | rs6062862 | A | G | 0.094 | 0.092 | 0.020 | 6.68E-06 | 0.025 | 0.025 | 0.32 |
| Intestinibacter | rs62430350 | T | C | 0.058 | 0.151 | 0.035 | 6.84E-06 | 0.029 | 0.037 | 0.43 |
| Intestinibacter | rs68093214 | C | T | 0.258 | 0.066 | 0.015 | 9.26E-06 | -0.009 | 0.018 | 0.61 |
| Intestinibacter | rs6875660 | C | T | 0.123 | 0.089 | 0.019 | 3.06E-06 | -0.013 | 0.023 | 0.58 |
| Intestinibacter | rs893394 | G | A | 0.420 | 0.058 | 0.013 | 7.85E-06 | 0.018 | 0.016 | 0.26 |
| Intestinibacter | rs9348442 | C | T | 0.088 | 0.099 | 0.022 | 6.26E-06 | -0.018 | 0.021 | 0.40 |
| Coprococcus3 | rs10810043 | A | G | 0.390 | 0.052 | 0.012 | 9.27E-06 | 0.022 | 0.015 | 0.14 |
| Coprococcus3 | rs11077359 | T | C | 0.200 | -0.065 | 0.015 | 9.64E-06 | 0.019 | 0.019 | 0.33 |
| Coprococcus3 | rs11080344 | C | T | 0.474 | 0.052 | 0.011 | 4.79E-06 | 0.012 | 0.014 | 0.40 |
| Coprococcus3 | rs13247359 | G | A | 0.480 | 0.051 | 0.011 | 7.33E-06 | 0.007 | 0.016 | 0.68 |
| Coprococcus3 | rs13394391 | C | T | 0.160 | -0.071 | 0.015 | 2.20E-06 | -0.002 | 0.020 | 0.91 |
| Coprococcus3 | rs178271 | T | C | 0.053 | 0.145 | 0.029 | 7.81E-07 | 0.026 | 0.034 | 0.44 |
| Coprococcus3 | rs4575475 | G | A | 0.237 | 0.062 | 0.014 | 7.04E-06 | 0.025 | 0.019 | 0.19 |
| Coprococcus3 | rs7521171 | G | A | 0.338 | -0.060 | 0.013 | 4.32E-06 | -0.005 | 0.016 | 0.77 |
| Coprococcus3 | rs8100692 | T | C | 0.427 | 0.058 | 0.011 | 4.16E-07 | 0.008 | 0.014 | 0.59 |
| LachnospiraceaeUCG001 | rs10815577 | C | G | 0.387 | -0.068 | 0.014 | 1.72E-06 | 0.011 | 0.015 | 0.46 |
| LachnospiraceaeUCG001 | rs12131224 | C | T | 0.116 | 0.117 | 0.026 | 7.40E-06 | 0.043 | 0.027 | 0.11 |
| LachnospiraceaeUCG001 | rs2050911 | G | A | 0.304 | 0.075 | 0.015 | 1.11E-06 | -0.003 | 0.016 | 0.85 |
| LachnospiraceaeUCG001 | rs2371284 | T | C | 0.223 | -0.076 | 0.017 | 7.56E-06 | 0.005 | 0.018 | 0.76 |
| LachnospiraceaeUCG001 | rs437876 | T | C | 0.373 | 0.078 | 0.014 | 7.17E-08 | -0.003 | 0.016 | 0.85 |
| LachnospiraceaeUCG001 | rs4981345 | T | C | 0.351 | -0.068 | 0.015 | 6.09E-06 | 0.037 | 0.015 | 0.01 |
| LachnospiraceaeUCG001 | rs573933 | T | C | 0.102 | -0.108 | 0.023 | 3.11E-06 | 0.022 | 0.023 | 0.34 |
| LachnospiraceaeUCG001 | rs62496417 | T | G | 0.271 | -0.075 | 0.017 | 5.88E-06 | 0.006 | 0.017 | 0.72 |
| LachnospiraceaeUCG001 | rs7213933 | T | A | 0.195 | -0.082 | 0.018 | 9.02E-06 | 0.005 | 0.018 | 0.79 |
| LachnospiraceaeUCG001 | rs7341608 | T | C | 0.190 | -0.078 | 0.018 | 9.48E-06 | -0.013 | 0.020 | 0.53 |
| LachnospiraceaeUCG001 | rs74034332 | G | A | 0.046 | 0.168 | 0.038 | 3.33E-06 | 0.003 | 0.036 | 0.94 |
| LachnospiraceaeUCG001 | rs78848836 | A | G | 0.084 | -0.119 | 0.026 | 3.38E-06 | 0.006 | 0.024 | 0.82 |
| LachnospiraceaeUCG001 | rs79476906 | T | A | 0.144 | -0.087 | 0.020 | 8.27E-06 | 0.007 | 0.021 | 0.74 |
| LachnospiraceaeUCG001 | rs8104225 | A | G | 0.149 | 0.089 | 0.020 | 8.04E-06 | -0.037 | 0.023 | 0.11 |
| LachnospiraceaeUCG001 | rs9403580 | C | T | 0.109 | 0.108 | 0.023 | 3.47E-06 | 0.015 | 0.020 | 0.44 |
| LachnospiraceaeUCG001 | rs985416 | C | T | 0.206 | 0.097 | 0.018 | 1.46E-07 | -0.003 | 0.020 | 0.90 |
| Ruminiclostridium5 | rs10827477 | A | G | 0.315 | -0.055 | 0.012 | 2.19E-06 | -0.002 | 0.015 | 0.92 |
| Ruminiclostridium5 | rs113753996 | T | C | 0.137 | 0.082 | 0.017 | 3.99E-06 | 0.005 | 0.023 | 0.84 |
| Ruminiclostridium5 | rs1223978 | T | C | 0.480 | 0.048 | 0.011 | 8.16E-06 | 0.001 | 0.014 | 0.92 |
| Ruminiclostridium5 | rs1492620 | T | C | 0.081 | -0.083 | 0.018 | 3.53E-06 | -0.021 | 0.021 | 0.31 |
| Ruminiclostridium5 | rs243585 | C | G | 0.301 | -0.059 | 0.012 | 1.33E-06 | -0.020 | 0.018 | 0.26 |
| Ruminiclostridium5 | rs2482038 | C | A | 0.438 | 0.052 | 0.011 | 1.70E-06 | 0.012 | 0.014 | 0.40 |
| Ruminiclostridium5 | rs2791343 | T | C | 0.340 | 0.052 | 0.011 | 5.54E-06 | 0.009 | 0.015 | 0.57 |
| Ruminiclostridium5 | rs2801960 | C | G | 0.318 | 0.052 | 0.012 | 6.21E-06 | -0.018 | 0.016 | 0.26 |
| Ruminiclostridium5 | rs2833828 | G | A | 0.453 | 0.049 | 0.011 | 6.82E-06 | 0.011 | 0.014 | 0.43 |
| Ruminiclostridium5 | rs4955951 | A | G | 0.092 | -0.071 | 0.017 | 9.96E-06 | -0.028 | 0.025 | 0.26 |
| Ruminiclostridium5 | rs6121460 | G | A | 0.081 | 0.093 | 0.020 | 2.64E-06 | 0.005 | 0.025 | 0.84 |
| Ruminiclostridium5 | rs73002572 | G | C | 0.055 | 0.182 | 0.041 | 8.82E-06 | -0.027 | 0.026 | 0.29 |
| Ruminiclostridium5 | rs79968837 | A | G | 0.069 | -0.095 | 0.019 | 1.15E-06 | 0.015 | 0.023 | 0.51 |
| Ruminiclostridium5 | rs8053158 | A | G | 0.120 | -0.074 | 0.016 | 5.90E-06 | 0.002 | 0.020 | 0.92 |
| Ruminiclostridium6 | rs10829821 | T | C | 0.086 | -0.098 | 0.022 | 3.47E-06 | -0.026 | 0.025 | 0.30 |
| Ruminiclostridium6 | rs116969552 | A | G | 0.036 | -0.167 | 0.038 | 9.16E-06 | -0.024 | 0.039 | 0.54 |
| Ruminiclostridium6 | rs11992182 | A | C | 0.288 | 0.063 | 0.014 | 4.65E-06 | 0.023 | 0.016 | 0.16 |
| Ruminiclostridium6 | rs1871858 | C | G | 0.080 | -0.105 | 0.024 | 9.12E-06 | -0.021 | 0.024 | 0.38 |
| Ruminiclostridium6 | rs2548459 | C | T | 0.470 | 0.055 | 0.012 | 6.40E-06 | 0.022 | 0.016 | 0.16 |
| Ruminiclostridium6 | rs35362464 | C | A | 0.151 | 0.072 | 0.017 | 8.99E-06 | 0.008 | 0.020 | 0.67 |
| Ruminiclostridium6 | rs61060922 | T | G | 0.045 | 0.159 | 0.032 | 1.09E-06 | -0.062 | 0.033 | 0.06 |
| Ruminiclostridium6 | rs663262 | T | C | 0.052 | -0.135 | 0.031 | 3.39E-06 | 0.009 | 0.026 | 0.74 |
| Ruminiclostridium6 | rs67479537 | T | C | 0.080 | 0.119 | 0.026 | 9.30E-06 | 0.010 | 0.031 | 0.76 |
| Ruminiclostridium6 | rs71414120 | T | G | 0.077 | 0.201 | 0.041 | 1.08E-06 | -0.008 | 0.034 | 0.82 |
| Ruminiclostridium6 | rs72991535 | T | G | 0.060 | 0.136 | 0.030 | 4.95E-06 | -0.006 | 0.035 | 0.87 |
| Ruminiclostridium6 | rs73176030 | T | C | 0.318 | 0.059 | 0.013 | 7.29E-06 | 0.002 | 0.017 | 0.88 |
| Ruminiclostridium6 | rs77193512 | A | G | 0.167 | 0.074 | 0.015 | 1.30E-06 | 0.013 | 0.017 | 0.45 |
| Ruminiclostridium6 | rs792058 | G | A | 0.427 | 0.055 | 0.013 | 8.58E-06 | -0.005 | 0.015 | 0.75 |
| Ruminiclostridium6 | rs79968172 | G | A | 0.056 | 0.116 | 0.024 | 1.66E-06 | 0.022 | 0.028 | 0.43 |
| Ruminiclostridium6 | rs9555756 | A | C | 0.124 | -0.080 | 0.018 | 7.10E-06 | 0.037 | 0.021 | 0.08 |
| Bifidobacterium | rs10841473 | G | C | 0.270 | -0.062 | 0.013 | 1.65E-06 | -0.026 | 0.016 | 0.12 |
| Bifidobacterium | rs12022129 | G | A | 0.221 | 0.062 | 0.014 | 8.00E-06 | -0.008 | 0.016 | 0.64 |
| Bifidobacterium | rs182549 | C | T | 0.491 | 0.120 | 0.013 | 1.28E-20 | -0.026 | 0.018 | 0.15 |
| Bifidobacterium | rs2491158 | G | A | 0.182 | 0.071 | 0.016 | 8.05E-06 | 0.011 | 0.020 | 0.59 |
| Bifidobacterium | rs2686790 | T | C | 0.115 | 0.071 | 0.016 | 7.50E-06 | -0.026 | 0.018 | 0.16 |
| Bifidobacterium | rs540489 | T | G | 0.234 | -0.064 | 0.014 | 5.19E-06 | 0.003 | 0.017 | 0.88 |
| Bifidobacterium | rs55888705 | A | G | 0.387 | 0.055 | 0.012 | 6.67E-06 | 0.005 | 0.015 | 0.73 |
| Bifidobacterium | rs56108664 | T | C | 0.185 | 0.073 | 0.016 | 2.44E-06 | -0.013 | 0.020 | 0.51 |
| Bifidobacterium | rs5746486 | T | C | 0.354 | -0.054 | 0.012 | 9.00E-06 | 0.004 | 0.015 | 0.76 |
| Bifidobacterium | rs62181700 | G | A | 0.281 | -0.062 | 0.013 | 2.17E-06 | -0.001 | 0.016 | 0.93 |
| Bifidobacterium | rs7322849 | T | C | 0.096 | 0.112 | 0.020 | 1.08E-08 | 0.025 | 0.028 | 0.36 |
| Bifidobacterium | rs73797465 | T | G | 0.087 | -0.095 | 0.021 | 4.38E-06 | 0.060 | 0.030 | 0.05 |
| Bifidobacterium | rs75344046 | C | T | 0.050 | 0.232 | 0.051 | 4.86E-06 | -0.003 | 0.039 | 0.94 |
| Bifidobacterium | rs76671854 | C | G | 0.125 | -0.085 | 0.018 | 3.96E-06 | 0.013 | 0.024 | 0.60 |
| Bifidobacterium | rs857444 | C | T | 0.361 | 0.056 | 0.012 | 3.57E-06 | -0.018 | 0.015 | 0.24 |
| Paraprevotella | rs10842464 | T | C | 0.419 | -0.076 | 0.017 | 6.60E-06 | 0.011 | 0.016 | 0.49 |
| Paraprevotella | rs140997932 | T | C | 0.065 | -0.162 | 0.035 | 2.11E-06 | 0.005 | 0.031 | 0.88 |
| Paraprevotella | rs145020347 | A | G | 0.139 | -0.125 | 0.026 | 4.03E-06 | -0.011 | 0.024 | 0.65 |
| Paraprevotella | rs17109926 | A | G | 0.188 | -0.099 | 0.022 | 6.75E-06 | 0.006 | 0.017 | 0.72 |
| Paraprevotella | rs17785622 | A | G | 0.060 | 0.248 | 0.052 | 1.93E-06 | 0.021 | 0.034 | 0.55 |
| Paraprevotella | rs2081023 | A | G | 0.102 | -0.123 | 0.024 | 2.64E-07 | 0.003 | 0.019 | 0.87 |
| Paraprevotella | rs3008582 | T | C | 0.132 | 0.106 | 0.023 | 4.36E-06 | 0.031 | 0.018 | 0.08 |
| Paraprevotella | rs3801748 | G | A | 0.346 | 0.078 | 0.017 | 5.20E-06 | 0.005 | 0.015 | 0.73 |
| Paraprevotella | rs4756632 | G | T | 0.093 | -0.139 | 0.029 | 3.82E-06 | 0.007 | 0.023 | 0.75 |
| Paraprevotella | rs4767113 | C | T | 0.287 | 0.088 | 0.018 | 2.14E-06 | -0.004 | 0.016 | 0.78 |
| Paraprevotella | rs7240324 | T | G | 0.191 | -0.102 | 0.023 | 5.96E-06 | -0.005 | 0.020 | 0.82 |
| Paraprevotella | rs9602779 | A | C | 0.173 | -0.107 | 0.022 | 6.93E-07 | 0.014 | 0.019 | 0.47 |
| Paraprevotella | rs9900242 | A | G | 0.361 | -0.085 | 0.018 | 1.14E-06 | 0.000 | 0.014 | 0.98 |
| RuminococcaceaeUCG005 | rs10873449 | T | C | 0.169 | 0.065 | 0.014 | 4.11E-06 | -0.001 | 0.018 | 0.97 |
| RuminococcaceaeUCG005 | rs10937802 | G | A | 0.097 | 0.076 | 0.017 | 8.17E-06 | 0.009 | 0.020 | 0.66 |
| RuminococcaceaeUCG005 | rs10950694 | T | C | 0.424 | 0.058 | 0.011 | 4.30E-07 | 0.033 | 0.015 | 0.02 |
| RuminococcaceaeUCG005 | rs114279581 | A | G | 0.048 | -0.147 | 0.032 | 3.22E-06 | 0.002 | 0.029 | 0.95 |
| RuminococcaceaeUCG005 | rs12288512 | A | G | 0.180 | 0.067 | 0.014 | 3.10E-06 | 0.032 | 0.019 | 0.10 |
| RuminococcaceaeUCG005 | rs12458218 | T | C | 0.177 | 0.068 | 0.014 | 2.41E-06 | 0.036 | 0.018 | 0.04 |
| RuminococcaceaeUCG005 | rs2893871 | G | A | 0.152 | -0.074 | 0.016 | 3.54E-06 | 0.000 | 0.019 | 0.98 |
| RuminococcaceaeUCG005 | rs34781347 | G | A | 0.060 | 0.189 | 0.039 | 6.05E-07 | -0.033 | 0.034 | 0.33 |
| RuminococcaceaeUCG005 | rs35166120 | C | G | 0.185 | -0.069 | 0.015 | 3.75E-06 | 0.014 | 0.018 | 0.44 |
| RuminococcaceaeUCG005 | rs394449 | A | T | 0.179 | 0.069 | 0.015 | 2.60E-06 | -0.008 | 0.018 | 0.65 |
| RuminococcaceaeUCG005 | rs55793120 | T | C | 0.051 | 0.122 | 0.028 | 7.37E-06 | -0.057 | 0.034 | 0.10 |
| RuminococcaceaeUCG005 | rs60081663 | C | G | 0.053 | 0.158 | 0.032 | 9.28E-07 | -0.040 | 0.037 | 0.29 |
| RuminococcaceaeUCG005 | rs72776570 | C | A | 0.083 | 0.087 | 0.020 | 5.36E-06 | 0.066 | 0.027 | 0.01 |
| RuminococcaceaeUCG005 | rs7449320 | C | A | 0.221 | 0.060 | 0.013 | 4.81E-06 | -0.006 | 0.016 | 0.72 |
| RuminococcaceaeUCG005 | rs7555878 | A | G | 0.280 | 0.059 | 0.013 | 2.81E-06 | 0.029 | 0.016 | 0.07 |
| RuminococcaceaeUCG005 | rs7586445 | G | A | 0.126 | 0.078 | 0.018 | 8.81E-06 | 0.045 | 0.022 | 0.04 |
| RuminococcaceaeUCG005 | rs898577 | T | C | 0.055 | -0.123 | 0.029 | 7.46E-06 | -0.015 | 0.027 | 0.57 |
| Holdemania | rs10885477 | T | C | 0.062 | -0.135 | 0.030 | 8.60E-06 | 0.018 | 0.024 | 0.47 |
| Holdemania | rs11080063 | G | A | 0.466 | -0.067 | 0.015 | 6.67E-06 | -0.010 | 0.015 | 0.51 |
| Holdemania | rs111745969 | A | G | 0.080 | 0.121 | 0.027 | 3.71E-06 | -0.028 | 0.026 | 0.27 |
| Holdemania | rs113593397 | A | G | 0.102 | -0.129 | 0.028 | 9.36E-06 | -0.020 | 0.028 | 0.47 |
| Holdemania | rs116500994 | G | T | 0.052 | -0.138 | 0.029 | 2.34E-06 | 0.030 | 0.031 | 0.32 |
| Holdemania | rs12701617 | A | G | 0.458 | -0.066 | 0.015 | 9.52E-06 | 0.004 | 0.014 | 0.80 |
| Holdemania | rs150096134 | T | A | 0.063 | 0.162 | 0.033 | 2.38E-06 | -0.009 | 0.023 | 0.71 |
| Holdemania | rs1867876 | T | C | 0.294 | 0.084 | 0.016 | 2.74E-07 | 0.009 | 0.015 | 0.55 |
| Holdemania | rs41438744 | C | G | 0.073 | -0.125 | 0.027 | 2.44E-06 | -0.008 | 0.029 | 0.78 |
| Holdemania | rs4146507 | C | T | 0.222 | 0.079 | 0.018 | 7.23E-06 | 0.006 | 0.017 | 0.70 |
| Holdemania | rs55888180 | C | G | 0.088 | 0.129 | 0.028 | 5.89E-06 | -0.011 | 0.029 | 0.69 |
| Holdemania | rs6133067 | T | C | 0.311 | 0.091 | 0.018 | 5.17E-07 | -0.017 | 0.023 | 0.45 |
| Holdemania | rs73139538 | G | A | 0.053 | -0.149 | 0.033 | 7.77E-06 | -0.013 | 0.035 | 0.71 |
| Holdemania | rs77293403 | A | G | 0.046 | 0.165 | 0.034 | 1.77E-06 | 0.016 | 0.034 | 0.64 |
| Holdemania | rs80149660 | C | T | 0.059 | -0.233 | 0.052 | 6.04E-06 | 0.009 | 0.033 | 0.79 |
| Holdemania | rs9500080 | C | T | 0.235 | 0.093 | 0.018 | 4.09E-07 | 0.012 | 0.017 | 0.47 |
| Holdemania | rs9529719 | T | C | 0.325 | 0.074 | 0.016 | 5.97E-06 | -0.006 | 0.015 | 0.71 |
| Holdemania | rs967319 | T | C | 0.247 | 0.079 | 0.018 | 8.38E-06 | -0.010 | 0.017 | 0.58 |
| Collinsella | rs10890671 | T | C | 0.527 | -0.054 | 0.012 | 6.52E-06 | -0.022 | 0.014 | 0.13 |
| Collinsella | rs11597285 | G | T | 0.425 | -0.054 | 0.012 | 9.38E-06 | 0.013 | 0.015 | 0.40 |
| Collinsella | rs12921100 | A | T | 0.317 | 0.056 | 0.013 | 8.23E-06 | 0.006 | 0.016 | 0.71 |
| Collinsella | rs1496626 | T | C | 0.154 | -0.072 | 0.016 | 6.78E-06 | 0.004 | 0.020 | 0.85 |
| Collinsella | rs149807560 | C | A | 0.090 | -0.104 | 0.024 | 7.10E-06 | -0.023 | 0.026 | 0.38 |
| Collinsella | rs2103510 | G | A | 0.131 | 0.079 | 0.017 | 2.42E-06 | 0.005 | 0.020 | 0.80 |
| Collinsella | rs59414781 | C | G | 0.180 | 0.067 | 0.015 | 9.15E-06 | 0.036 | 0.018 | 0.04 |
| Collinsella | rs62448871 | C | A | 0.461 | -0.054 | 0.012 | 6.78E-06 | -0.009 | 0.015 | 0.53 |
| Collinsella | rs73052258 | G | A | 0.096 | 0.093 | 0.020 | 1.72E-06 | -0.036 | 0.025 | 0.15 |
| Collinsella | rs75672793 | A | G | 0.071 | -0.109 | 0.024 | 6.14E-06 | 0.019 | 0.031 | 0.54 |
| Collinsella | rs9541268 | C | A | 0.096 | 0.096 | 0.020 | 8.79E-07 | -0.050 | 0.024 | 0.04 |
| Tyzzerella3 | rs10898797 | C | T | 0.157 | 0.122 | 0.027 | 8.85E-06 | -0.009 | 0.020 | 0.67 |
| Tyzzerella3 | rs112102233 | A | G | 0.067 | -0.216 | 0.048 | 6.18E-06 | -0.006 | 0.041 | 0.88 |
| Tyzzerella3 | rs1232220 | G | T | 0.126 | -0.144 | 0.032 | 7.91E-06 | 0.018 | 0.024 | 0.47 |
| Tyzzerella3 | rs17706273 | T | C | 0.152 | -0.140 | 0.027 | 5.88E-07 | 0.007 | 0.022 | 0.74 |
| Tyzzerella3 | rs17809157 | A | T | 0.099 | -0.164 | 0.034 | 1.54E-06 | -0.018 | 0.027 | 0.49 |
| Tyzzerella3 | rs191093 | G | A | 0.080 | 0.159 | 0.035 | 6.76E-06 | -0.021 | 0.028 | 0.46 |
| Tyzzerella3 | rs4904512 | T | C | 0.199 | -0.117 | 0.025 | 3.09E-06 | -0.001 | 0.018 | 0.96 |
| Tyzzerella3 | rs55799124 | A | G | 0.207 | -0.114 | 0.024 | 1.34E-06 | 0.009 | 0.018 | 0.63 |
| Tyzzerella3 | rs67476743 | T | G | 0.285 | 0.132 | 0.022 | 3.74E-09 | 0.001 | 0.015 | 0.93 |
| Tyzzerella3 | rs6920448 | C | T | 0.115 | -0.141 | 0.031 | 4.15E-06 | 0.018 | 0.022 | 0.41 |
| Tyzzerella3 | rs7019909 | T | C | 0.101 | 0.144 | 0.030 | 1.76E-06 | 0.024 | 0.020 | 0.23 |
| Tyzzerella3 | rs7333521 | T | C | 0.043 | -0.207 | 0.045 | 4.88E-06 | 0.043 | 0.036 | 0.24 |
| Tyzzerella3 | rs75091807 | G | T | 0.091 | -0.185 | 0.038 | 1.71E-06 | 0.085 | 0.029 | 0.00 |
| Tyzzerella3 | rs7561370 | T | C | 0.122 | 0.131 | 0.029 | 1.52E-06 | -0.009 | 0.019 | 0.64 |
| Parasutterella | rs10899911 | A | G | 0.272 | -0.072 | 0.015 | 1.15E-06 | -0.005 | 0.016 | 0.74 |
| Parasutterella | rs11715853 | G | A | 0.314 | -0.066 | 0.015 | 6.23E-06 | 0.011 | 0.016 | 0.49 |
| Parasutterella | rs1403396 | A | T | 0.215 | -0.076 | 0.016 | 2.76E-06 | -0.019 | 0.017 | 0.28 |
| Parasutterella | rs2090816 | A | C | 0.157 | 0.084 | 0.018 | 2.90E-06 | 0.003 | 0.020 | 0.88 |
| Parasutterella | rs2387977 | T | C | 0.458 | -0.068 | 0.013 | 5.38E-07 | -0.035 | 0.018 | 0.06 |
| Parasutterella | rs35055552 | T | C | 0.078 | 0.110 | 0.024 | 3.35E-06 | 0.006 | 0.026 | 0.80 |
| Parasutterella | rs35414597 | T | A | 0.339 | -0.068 | 0.014 | 1.51E-06 | -0.009 | 0.015 | 0.55 |
| Parasutterella | rs55877868 | A | C | 0.096 | -0.104 | 0.023 | 2.87E-06 | 0.021 | 0.026 | 0.43 |
| Parasutterella | rs62273907 | A | G | 0.050 | 0.229 | 0.050 | 5.88E-06 | 0.041 | 0.035 | 0.24 |
| Parasutterella | rs6809952 | G | A | 0.273 | -0.068 | 0.015 | 8.13E-06 | 0.029 | 0.016 | 0.06 |
| Parasutterella | rs6828768 | C | T | 0.500 | 0.064 | 0.013 | 1.78E-06 | 0.021 | 0.014 | 0.13 |
| Parasutterella | rs7303158 | C | T | 0.442 | 0.065 | 0.013 | 1.33E-06 | 0.004 | 0.014 | 0.81 |
| Parasutterella | rs7311004 | T | C | 0.376 | -0.062 | 0.014 | 5.92E-06 | 0.021 | 0.015 | 0.16 |
| Parasutterella | rs7572229 | G | A | 0.490 | 0.066 | 0.013 | 6.32E-07 | 0.011 | 0.014 | 0.46 |
| Parasutterella | rs78383039 | T | C | 0.067 | -0.146 | 0.030 | 1.57E-06 | -0.033 | 0.025 | 0.19 |
| Parasutterella | rs8039785 | T | G | 0.452 | 0.062 | 0.013 | 3.62E-06 | 0.007 | 0.014 | 0.64 |
| Parasutterella | rs823424 | G | A | 0.210 | -0.071 | 0.016 | 4.95E-06 | -0.004 | 0.017 | 0.79 |
| Ruminococcustorquesgroup | rs10904297 | A | G | 0.030 | -0.168 | 0.039 | 2.69E-06 | 0.002 | 0.026 | 0.93 |
| Ruminococcustorquesgroup | rs10967781 | C | A | 0.318 | 0.051 | 0.011 | 8.37E-06 | -0.022 | 0.016 | 0.16 |
| Ruminococcustorquesgroup | rs12434631 | A | G | 0.140 | 0.075 | 0.015 | 2.77E-06 | 0.068 | 0.019 | 0.00 |
| Ruminococcustorquesgroup | rs13154778 | T | A | 0.186 | 0.056 | 0.013 | 7.16E-06 | 0.006 | 0.017 | 0.71 |
| Ruminococcustorquesgroup | rs1475330 | T | C | 0.301 | 0.052 | 0.012 | 8.13E-06 | 0.004 | 0.016 | 0.80 |
| Ruminococcustorquesgroup | rs1972694 | T | A | 0.184 | -0.061 | 0.014 | 8.93E-06 | 0.021 | 0.018 | 0.26 |
| Ruminococcustorquesgroup | rs35866622 | T | C | 0.440 | -0.061 | 0.011 | 2.21E-08 | 0.019 | 0.016 | 0.23 |
| Ruminococcustorquesgroup | rs4073731 | T | C | 0.176 | 0.065 | 0.014 | 4.05E-06 | 0.008 | 0.018 | 0.64 |
| Ruminococcustorquesgroup | rs73130967 | A | T | 0.112 | 0.077 | 0.017 | 3.71E-06 | 0.035 | 0.025 | 0.16 |
| Ruminococcustorquesgroup | rs77034621 | T | G | 0.053 | -0.152 | 0.034 | 6.07E-06 | -0.035 | 0.038 | 0.36 |
| Ruminococcustorquesgroup | rs773123 | T | A | 0.112 | 0.082 | 0.017 | 1.59E-06 | -0.028 | 0.024 | 0.25 |
| Ruminococcustorquesgroup | rs8080469 | G | A | 0.519 | 0.049 | 0.011 | 3.50E-06 | 0.021 | 0.018 | 0.26 |
| Ruminococcustorquesgroup | rs8141465 | A | G | 0.543 | 0.048 | 0.011 | 9.65E-06 | 0.004 | 0.017 | 0.81 |
| RuminococcaceaeUCG002 | rs10916131 | C | T | 0.138 | -0.069 | 0.015 | 2.87E-06 | -0.007 | 0.019 | 0.70 |
| RuminococcaceaeUCG002 | rs10927423 | C | A | 0.183 | -0.071 | 0.015 | 8.50E-07 | -0.020 | 0.019 | 0.30 |
| RuminococcaceaeUCG002 | rs10964441 | G | A | 0.053 | -0.149 | 0.034 | 7.45E-06 | 0.034 | 0.022 | 0.13 |
| RuminococcaceaeUCG002 | rs113147300 | A | G | 0.132 | -0.076 | 0.016 | 7.69E-06 | 0.003 | 0.023 | 0.91 |
| RuminococcaceaeUCG002 | rs11607472 | A | G | 0.107 | -0.078 | 0.018 | 7.19E-06 | -0.033 | 0.026 | 0.21 |
| RuminococcaceaeUCG002 | rs116974815 | C | A | 0.058 | -0.190 | 0.040 | 2.03E-06 | -0.058 | 0.033 | 0.08 |
| RuminococcaceaeUCG002 | rs11750293 | G | T | 0.281 | -0.058 | 0.012 | 1.76E-06 | 0.006 | 0.015 | 0.70 |
| RuminococcaceaeUCG002 | rs12463378 | A | G | 0.418 | -0.052 | 0.011 | 2.96E-06 | 0.001 | 0.015 | 0.93 |
| RuminococcaceaeUCG002 | rs15256 | C | T | 0.087 | 0.073 | 0.017 | 9.46E-06 | -0.007 | 0.021 | 0.76 |
| RuminococcaceaeUCG002 | rs362417 | G | C | 0.266 | -0.055 | 0.012 | 7.80E-06 | 0.019 | 0.016 | 0.25 |
| RuminococcaceaeUCG002 | rs55793120 | T | C | 0.051 | 0.137 | 0.027 | 4.81E-07 | -0.057 | 0.034 | 0.10 |
| RuminococcaceaeUCG002 | rs56030423 | G | A | 0.089 | -0.098 | 0.022 | 6.30E-06 | 0.058 | 0.033 | 0.08 |
| RuminococcaceaeUCG002 | rs57079348 | T | G | 0.121 | -0.077 | 0.017 | 7.22E-06 | -0.052 | 0.027 | 0.05 |
| RuminococcaceaeUCG002 | rs6542556 | A | G | 0.345 | 0.051 | 0.011 | 7.86E-06 | 0.009 | 0.015 | 0.55 |
| RuminococcaceaeUCG002 | rs6793778 | C | T | 0.262 | -0.056 | 0.013 | 9.81E-06 | -0.005 | 0.016 | 0.77 |
| RuminococcaceaeUCG002 | rs7120052 | A | C | 0.176 | 0.062 | 0.014 | 1.97E-06 | 0.005 | 0.017 | 0.78 |
| RuminococcaceaeUCG002 | rs7155595 | C | A | 0.309 | 0.057 | 0.012 | 1.15E-06 | 0.026 | 0.016 | 0.10 |
| RuminococcaceaeUCG002 | rs7249614 | A | G | 0.385 | -0.049 | 0.011 | 9.07E-06 | 0.015 | 0.033 | 0.66 |
| RuminococcaceaeUCG002 | rs72874194 | G | C | 0.099 | -0.077 | 0.017 | 3.47E-06 | -0.004 | 0.020 | 0.83 |
| RuminococcaceaeUCG002 | rs7342369 | C | A | 0.323 | -0.053 | 0.012 | 5.66E-06 | -0.022 | 0.017 | 0.18 |
| RuminococcaceaeUCG002 | rs76847269 | A | G | 0.057 | 0.164 | 0.036 | 5.17E-06 | -0.027 | 0.037 | 0.47 |
| RuminococcaceaeUCG002 | rs77564310 | A | C | 0.179 | -0.071 | 0.014 | 3.29E-07 | 0.034 | 0.018 | 0.06 |
| RuminococcaceaeUCG002 | rs79016051 | C | T | 0.091 | -0.089 | 0.019 | 2.34E-06 | 0.015 | 0.026 | 0.55 |
| RuminococcaceaeUCG002 | rs882348 | A | G | 0.106 | -0.080 | 0.018 | 5.45E-06 | -0.013 | 0.021 | 0.55 |
| Faecalibacterium | rs10927394 | G | T | 0.027 | -0.232 | 0.051 | 7.02E-06 | -0.030 | 0.040 | 0.45 |
| Faecalibacterium | rs114946999 | C | T | 0.098 | -0.086 | 0.019 | 5.70E-06 | -0.007 | 0.026 | 0.80 |
| Faecalibacterium | rs11776390 | T | C | 0.090 | -0.078 | 0.017 | 6.40E-06 | 0.051 | 0.024 | 0.03 |
| Faecalibacterium | rs12320842 | C | G | 0.132 | 0.095 | 0.016 | 7.57E-09 | 0.024 | 0.025 | 0.33 |
| Faecalibacterium | rs1271565 | C | T | 0.272 | -0.058 | 0.012 | 1.30E-06 | -0.005 | 0.016 | 0.77 |
| Faecalibacterium | rs12753492 | A | C | 0.149 | 0.064 | 0.015 | 8.80E-06 | -0.019 | 0.021 | 0.38 |
| Faecalibacterium | rs2835874 | T | C | 0.070 | -0.087 | 0.020 | 7.54E-06 | -0.015 | 0.028 | 0.60 |
| Faecalibacterium | rs28376661 | C | G | 0.374 | 0.050 | 0.011 | 3.66E-06 | 0.003 | 0.015 | 0.84 |
| Faecalibacterium | rs61875484 | C | G | 0.091 | 0.082 | 0.018 | 9.18E-06 | 0.038 | 0.027 | 0.16 |
| Faecalibacterium | rs6910935 | A | G | 0.054 | 0.135 | 0.028 | 1.38E-06 | 0.051 | 0.033 | 0.12 |
| Faecalibacterium | rs75499067 | C | T | 0.064 | 0.228 | 0.047 | 1.76E-06 | -0.001 | 0.034 | 0.98 |
| Faecalibacterium | rs79656633 | T | C | 0.105 | 0.146 | 0.032 | 8.14E-06 | -0.008 | 0.034 | 0.80 |
| Faecalibacterium | rs9536330 | T | C | 0.409 | -0.048 | 0.011 | 5.33E-06 | 0.020 | 0.015 | 0.18 |
| Ruminococcusgauvreauiigroup | rs10931481 | G | A | 0.311 | 0.061 | 0.013 | 3.38E-06 | -0.021 | 0.015 | 0.17 |
| Ruminococcusgauvreauiigroup | rs12079579 | A | G | 0.073 | 0.096 | 0.021 | 5.04E-06 | -0.070 | 0.025 | 0.00 |
| Ruminococcusgauvreauiigroup | rs12539819 | C | T | 0.095 | 0.111 | 0.024 | 4.49E-06 | -0.011 | 0.030 | 0.72 |
| Ruminococcusgauvreauiigroup | rs13188803 | T | A | 0.188 | 0.071 | 0.016 | 7.28E-06 | -0.009 | 0.019 | 0.63 |
| Ruminococcusgauvreauiigroup | rs1391597 | C | T | 0.381 | 0.059 | 0.012 | 1.86E-06 | -0.001 | 0.015 | 0.96 |
| Ruminococcusgauvreauiigroup | rs2047242 | A | G | 0.297 | -0.068 | 0.013 | 2.46E-07 | 0.005 | 0.017 | 0.74 |
| Ruminococcusgauvreauiigroup | rs2105937 | A | G | 0.334 | 0.058 | 0.013 | 5.10E-06 | 0.015 | 0.019 | 0.41 |
| Ruminococcusgauvreauiigroup | rs2166943 | A | C | 0.438 | 0.057 | 0.012 | 5.28E-06 | 0.001 | 0.014 | 0.94 |
| Ruminococcusgauvreauiigroup | rs289410 | G | A | 0.268 | -0.065 | 0.014 | 2.27E-06 | 0.004 | 0.016 | 0.81 |
| Ruminococcusgauvreauiigroup | rs431418 | A | G | 0.119 | -0.095 | 0.021 | 5.54E-06 | 0.027 | 0.022 | 0.21 |
| Ruminococcusgauvreauiigroup | rs71386687 | T | G | 0.060 | 0.121 | 0.024 | 2.91E-07 | -0.031 | 0.029 | 0.28 |
| Ruminococcusgauvreauiigroup | rs73802842 | C | A | 0.143 | 0.074 | 0.017 | 7.48E-06 | -0.016 | 0.018 | 0.39 |
| Ruminococcusgauvreauiigroup | rs9870933 | A | G | 0.409 | 0.062 | 0.013 | 8.49E-07 | -0.029 | 0.015 | 0.04 |
| LachnospiraceaeNK4A136group | rs10952110 | G | T | 0.448 | 0.049 | 0.011 | 9.08E-06 | -0.014 | 0.015 | 0.33 |
| LachnospiraceaeNK4A136group | rs11263806 | A | G | 0.278 | -0.052 | 0.012 | 5.07E-06 | -0.025 | 0.016 | 0.12 |
| LachnospiraceaeNK4A136group | rs12362320 | G | C | 0.336 | 0.057 | 0.012 | 8.04E-07 | 0.006 | 0.015 | 0.70 |
| LachnospiraceaeNK4A136group | rs12611395 | A | G | 0.090 | -0.090 | 0.020 | 5.83E-06 | -0.029 | 0.027 | 0.27 |
| LachnospiraceaeNK4A136group | rs160061 | A | G | 0.468 | 0.051 | 0.011 | 2.12E-06 | -0.015 | 0.014 | 0.29 |
| LachnospiraceaeNK4A136group | rs28540839 | A | C | 0.487 | 0.051 | 0.011 | 9.34E-06 | -0.021 | 0.015 | 0.16 |
| LachnospiraceaeNK4A136group | rs2880566 | T | C | 0.188 | 0.060 | 0.013 | 5.61E-06 | -0.032 | 0.019 | 0.09 |
| LachnospiraceaeNK4A136group | rs4955932 | T | C | 0.396 | -0.049 | 0.011 | 7.05E-06 | 0.001 | 0.015 | 0.96 |
| LachnospiraceaeNK4A136group | rs59805249 | T | C | 0.091 | 0.094 | 0.021 | 9.45E-06 | -0.019 | 0.024 | 0.42 |
| LachnospiraceaeNK4A136group | rs68104925 | T | C | 0.371 | -0.055 | 0.012 | 2.37E-06 | 0.019 | 0.015 | 0.21 |
| LachnospiraceaeNK4A136group | rs7073658 | T | G | 0.405 | -0.050 | 0.011 | 5.27E-06 | -0.010 | 0.014 | 0.49 |
| LachnospiraceaeNK4A136group | rs73044693 | A | G | 0.067 | -0.108 | 0.023 | 3.57E-06 | 0.015 | 0.030 | 0.62 |
| LachnospiraceaeNK4A136group | rs7616165 | G | T | 0.037 | -0.231 | 0.048 | 2.77E-06 | -0.031 | 0.033 | 0.35 |
| LachnospiraceaeNK4A136group | rs76193507 | A | G | 0.057 | -0.230 | 0.050 | 2.93E-06 | -0.010 | 0.035 | 0.77 |
| LachnospiraceaeNK4A136group | rs7832116 | A | G | 0.146 | -0.071 | 0.015 | 3.57E-06 | 0.036 | 0.019 | 0.06 |
| LachnospiraceaeNK4A136group | rs954878 | A | G | 0.450 | -0.052 | 0.011 | 1.78E-06 | 0.000 | 0.014 | 0.98 |
| RuminococcaceaeUCG004 | rs10976229 | T | G | 0.141 | 0.096 | 0.021 | 7.04E-06 | -0.014 | 0.023 | 0.56 |
| RuminococcaceaeUCG004 | rs11961899 | G | A | 0.271 | -0.071 | 0.016 | 9.18E-06 | 0.005 | 0.016 | 0.77 |
| RuminococcaceaeUCG004 | rs12125734 | G | T | 0.087 | 0.134 | 0.026 | 2.09E-07 | 0.034 | 0.026 | 0.18 |
| RuminococcaceaeUCG004 | rs2248146 | T | C | 0.322 | 0.069 | 0.015 | 8.20E-06 | -0.024 | 0.015 | 0.11 |
| RuminococcaceaeUCG004 | rs3800154 | A | C | 0.221 | -0.080 | 0.018 | 6.12E-06 | 0.002 | 0.017 | 0.92 |
| RuminococcaceaeUCG004 | rs511258 | G | A | 0.263 | -0.076 | 0.016 | 4.52E-06 | -0.008 | 0.016 | 0.61 |
| RuminococcaceaeUCG004 | rs550351 | A | C | 0.440 | 0.079 | 0.018 | 9.43E-06 | 0.010 | 0.023 | 0.67 |
| RuminococcaceaeUCG004 | rs6769553 | A | G | 0.284 | 0.085 | 0.016 | 7.91E-08 | -0.010 | 0.015 | 0.53 |
| RuminococcaceaeUCG004 | rs7123615 | C | G | 0.169 | -0.079 | 0.018 | 7.09E-06 | 0.029 | 0.019 | 0.13 |
| RuminococcaceaeUCG004 | rs7569771 | A | G | 0.217 | -0.076 | 0.017 | 8.12E-06 | -0.028 | 0.016 | 0.09 |
| RuminococcaceaeUCG004 | rs872501 | G | A | 0.127 | 0.116 | 0.026 | 5.81E-06 | 0.000 | 0.039 | 0.99 |
| RuminococcaceaeUCG004 | rs9818949 | G | T | 0.219 | 0.086 | 0.019 | 5.39E-06 | 0.010 | 0.020 | 0.64 |
| Eisenbergiella | rs11027642 | C | T | 0.124 | 0.129 | 0.028 | 4.92E-06 | -0.028 | 0.019 | 0.13 |
| Eisenbergiella | rs11079158 | T | C | 0.220 | 0.101 | 0.023 | 7.35E-06 | 0.026 | 0.018 | 0.15 |
| Eisenbergiella | rs11938607 | T | C | 0.263 | 0.098 | 0.022 | 8.22E-06 | 0.019 | 0.016 | 0.24 |
| Eisenbergiella | rs12257723 | A | C | 0.339 | -0.095 | 0.021 | 8.85E-06 | -0.013 | 0.015 | 0.39 |
| Eisenbergiella | rs12278566 | T | A | 0.175 | -0.121 | 0.025 | 1.65E-06 | -0.007 | 0.018 | 0.70 |
| Eisenbergiella | rs12710729 | C | A | 0.371 | 0.089 | 0.020 | 9.84E-06 | 0.020 | 0.017 | 0.24 |
| Eisenbergiella | rs13258851 | A | G | 0.086 | 0.137 | 0.030 | 7.75E-06 | 0.010 | 0.020 | 0.62 |
| Eisenbergiella | rs1508033 | A | C | 0.350 | 0.092 | 0.020 | 3.23E-06 | -0.001 | 0.015 | 0.96 |
| Eisenbergiella | rs1553971 | T | G | 0.161 | 0.121 | 0.026 | 5.27E-06 | 0.000 | 0.019 | 0.99 |
| Eisenbergiella | rs2683098 | C | T | 0.224 | 0.107 | 0.023 | 2.24E-06 | 0.020 | 0.017 | 0.22 |
| Eisenbergiella | rs3812426 | G | A | 0.208 | 0.106 | 0.022 | 2.72E-06 | -0.005 | 0.017 | 0.80 |
| Eisenbergiella | rs4462860 | G | A | 0.344 | 0.094 | 0.020 | 4.16E-06 | 0.022 | 0.015 | 0.14 |
| Bilophila | rs11069458 | T | C | 0.190 | -0.068 | 0.016 | 7.72E-06 | 0.006 | 0.018 | 0.73 |
| Bilophila | rs116261629 | G | C | 0.072 | 0.128 | 0.026 | 8.62E-07 | 0.066 | 0.031 | 0.03 |
| Bilophila | rs1241171 | G | A | 0.216 | -0.069 | 0.015 | 4.24E-06 | -0.018 | 0.017 | 0.28 |
| Bilophila | rs1571225 | C | T | 0.149 | 0.083 | 0.017 | 1.12E-06 | 0.032 | 0.019 | 0.08 |
| Bilophila | rs1917709 | A | T | 0.054 | 0.119 | 0.027 | 7.37E-06 | 0.028 | 0.036 | 0.44 |
| Bilophila | rs1969927 | G | A | 0.380 | 0.056 | 0.013 | 9.07E-06 | 0.003 | 0.015 | 0.83 |
| Bilophila | rs2713349 | A | T | 0.255 | 0.062 | 0.014 | 8.63E-06 | -0.004 | 0.016 | 0.80 |
| Bilophila | rs2728491 | G | T | 0.310 | -0.063 | 0.014 | 6.33E-06 | 0.027 | 0.016 | 0.09 |
| Bilophila | rs3827020 | C | T | 0.190 | 0.077 | 0.016 | 1.79E-06 | 0.006 | 0.018 | 0.73 |
| Bilophila | rs4798126 | G | A | 0.150 | 0.073 | 0.017 | 7.15E-06 | -0.027 | 0.018 | 0.14 |
| Bilophila | rs542415 | T | C | 0.340 | -0.061 | 0.013 | 4.71E-06 | -0.019 | 0.016 | 0.21 |
| Bilophila | rs60178956 | G | A | 0.235 | -0.062 | 0.014 | 8.06E-06 | -0.020 | 0.016 | 0.20 |
| Bilophila | rs6793291 | C | A | 0.092 | 0.113 | 0.024 | 3.11E-06 | -0.014 | 0.026 | 0.58 |
| Bilophila | rs72676854 | T | C | 0.066 | 0.123 | 0.027 | 5.62E-06 | 0.005 | 0.033 | 0.87 |
| Bilophila | rs7802841 | C | A | 0.351 | 0.067 | 0.014 | 1.77E-06 | 0.001 | 0.017 | 0.94 |
| Bilophila | rs9899990 | A | G | 0.066 | -0.103 | 0.023 | 9.07E-06 | 0.013 | 0.029 | 0.66 |
| Alistipes | rs1107244 | G | A | 0.079 | 0.076 | 0.017 | 3.59E-06 | -0.017 | 0.021 | 0.42 |
| Alistipes | rs11769002 | G | A | 0.401 | -0.053 | 0.011 | 1.45E-06 | 0.018 | 0.016 | 0.26 |
| Alistipes | rs11958296 | A | G | 0.071 | -0.098 | 0.022 | 9.30E-06 | 0.021 | 0.032 | 0.51 |
| Alistipes | rs12990744 | C | T | 0.123 | -0.078 | 0.017 | 8.21E-06 | -0.018 | 0.024 | 0.45 |
| Alistipes | rs1689282 | A | C | 0.340 | -0.052 | 0.011 | 5.28E-06 | 0.015 | 0.015 | 0.32 |
| Alistipes | rs2290844 | C | T | 0.113 | 0.081 | 0.019 | 9.10E-06 | 0.040 | 0.024 | 0.09 |
| Alistipes | rs2450745 | A | C | 0.083 | -0.081 | 0.018 | 7.12E-06 | 0.028 | 0.025 | 0.26 |
| Alistipes | rs2875322 | T | C | 0.207 | -0.058 | 0.013 | 8.78E-06 | 0.034 | 0.017 | 0.04 |
| Alistipes | rs34417064 | A | G | 0.512 | -0.048 | 0.011 | 7.01E-06 | 0.006 | 0.014 | 0.68 |
| Alistipes | rs4810359 | A | G | 0.147 | -0.065 | 0.015 | 7.50E-06 | 0.027 | 0.019 | 0.15 |
| Alistipes | rs62576416 | T | C | 0.398 | 0.049 | 0.011 | 7.50E-06 | -0.010 | 0.017 | 0.57 |
| Alistipes | rs67281112 | G | C | 0.217 | 0.063 | 0.014 | 3.92E-06 | -0.007 | 0.019 | 0.71 |
| Alistipes | rs67705352 | T | G | 0.374 | -0.053 | 0.011 | 1.65E-06 | -0.025 | 0.019 | 0.19 |
| Alistipes | rs7129639 | C | A | 0.402 | -0.052 | 0.011 | 1.78E-06 | -0.031 | 0.015 | 0.03 |
| Alistipes | rs8130320 | A | G | 0.482 | -0.049 | 0.011 | 4.84E-06 | -0.003 | 0.014 | 0.81 |
| FamilyXIIIAD3011group | rs11126423 | C | T | 0.113 | 0.090 | 0.020 | 5.91E-06 | -0.028 | 0.021 | 0.18 |
| FamilyXIIIAD3011group | rs11736617 | G | A | 0.120 | -0.076 | 0.017 | 9.02E-06 | 0.001 | 0.020 | 0.95 |
| FamilyXIIIAD3011group | rs12812672 | T | C | 0.074 | -0.096 | 0.021 | 2.56E-06 | -0.023 | 0.027 | 0.40 |
| FamilyXIIIAD3011group | rs12911842 | A | T | 0.124 | -0.081 | 0.018 | 6.91E-06 | -0.037 | 0.021 | 0.08 |
| FamilyXIIIAD3011group | rs149302 | T | C | 0.213 | -0.065 | 0.014 | 7.48E-06 | -0.015 | 0.017 | 0.35 |
| FamilyXIIIAD3011group | rs16840310 | A | G | 0.372 | -0.061 | 0.012 | 6.75E-07 | -0.004 | 0.015 | 0.76 |
| FamilyXIIIAD3011group | rs16940167 | C | T | 0.169 | 0.073 | 0.016 | 3.91E-06 | -0.014 | 0.018 | 0.43 |
| FamilyXIIIAD3011group | rs17156849 | G | A | 0.063 | -0.113 | 0.025 | 4.19E-06 | -0.025 | 0.026 | 0.33 |
| FamilyXIIIAD3011group | rs62029761 | A | G | 0.068 | 0.129 | 0.028 | 3.89E-06 | -0.002 | 0.033 | 0.94 |
| FamilyXIIIAD3011group | rs62200412 | C | T | 0.171 | -0.080 | 0.016 | 5.80E-07 | 0.015 | 0.018 | 0.39 |
| FamilyXIIIAD3011group | rs72730932 | C | A | 0.164 | -0.090 | 0.018 | 6.89E-07 | -0.015 | 0.023 | 0.52 |
| FamilyXIIIAD3011group | rs739451 | C | T | 0.176 | 0.065 | 0.015 | 7.88E-06 | 0.005 | 0.019 | 0.80 |
| FamilyXIIIAD3011group | rs9276029 | A | G | 0.168 | -0.081 | 0.019 | 8.93E-06 | 0.062 | 0.023 | 0.01 |
| FamilyXIIIAD3011group | rs9837139 | A | G | 0.082 | 0.108 | 0.024 | 8.71E-06 | -0.018 | 0.027 | 0.50 |
| FamilyXIIIAD3011group | rs9852893 | C | G | 0.257 | 0.066 | 0.013 | 3.88E-07 | -0.012 | 0.015 | 0.43 |
| LachnospiraceaeUCG004 | rs11128180 | A | G | 0.224 | 0.065 | 0.014 | 4.52E-06 | -0.007 | 0.016 | 0.65 |
| LachnospiraceaeUCG004 | rs12072562 | T | C | 0.052 | 0.133 | 0.030 | 7.07E-06 | 0.018 | 0.035 | 0.60 |
| LachnospiraceaeUCG004 | rs12673420 | G | A | 0.441 | 0.055 | 0.012 | 2.98E-06 | -0.016 | 0.014 | 0.25 |
| LachnospiraceaeUCG004 | rs12747809 | G | A | 0.340 | -0.062 | 0.013 | 8.65E-07 | 0.033 | 0.015 | 0.03 |
| LachnospiraceaeUCG004 | rs12894272 | A | G | 0.300 | 0.058 | 0.013 | 4.34E-06 | 0.000 | 0.015 | 0.99 |
| LachnospiraceaeUCG004 | rs233486 | A | G | 0.124 | -0.080 | 0.018 | 6.28E-06 | 0.046 | 0.022 | 0.03 |
| LachnospiraceaeUCG004 | rs2444793 | C | T | 0.470 | -0.054 | 0.012 | 4.77E-06 | -0.001 | 0.014 | 0.97 |
| LachnospiraceaeUCG004 | rs2706242 | G | C | 0.093 | -0.090 | 0.020 | 9.84E-06 | -0.014 | 0.023 | 0.56 |
| LachnospiraceaeUCG004 | rs2726805 | A | G | 0.409 | 0.055 | 0.012 | 6.30E-06 | -0.026 | 0.015 | 0.09 |
| LachnospiraceaeUCG004 | rs2882478 | G | A | 0.455 | -0.058 | 0.012 | 1.21E-06 | -0.025 | 0.014 | 0.08 |
| LachnospiraceaeUCG004 | rs35182105 | A | G | 0.073 | -0.110 | 0.024 | 4.87E-06 | -0.013 | 0.031 | 0.69 |
| LachnospiraceaeUCG004 | rs6656451 | C | T | 0.439 | -0.054 | 0.012 | 5.57E-06 | -0.035 | 0.015 | 0.02 |
| LachnospiraceaeUCG004 | rs7629954 | A | G | 0.069 | 0.108 | 0.024 | 5.77E-06 | -0.031 | 0.032 | 0.33 |
| Veillonella | rs11141494 | G | A | 0.195 | -0.078 | 0.017 | 9.75E-06 | -0.036 | 0.019 | 0.06 |
| Veillonella | rs11614532 | G | C | 0.305 | 0.074 | 0.017 | 7.13E-06 | 0.002 | 0.019 | 0.92 |
| Veillonella | rs12679709 | C | G | 0.321 | -0.079 | 0.016 | 1.78E-06 | 0.006 | 0.016 | 0.72 |
| Veillonella | rs1882878 | A | G | 0.306 | -0.077 | 0.016 | 2.98E-06 | -0.021 | 0.016 | 0.18 |
| Veillonella | rs2013594 | T | C | 0.358 | -0.072 | 0.016 | 3.42E-06 | -0.009 | 0.015 | 0.55 |
| Veillonella | rs55807413 | A | G | 0.097 | 0.107 | 0.024 | 5.51E-06 | -0.048 | 0.024 | 0.05 |
| Veillonella | rs62376424 | C | T | 0.334 | -0.076 | 0.016 | 3.65E-06 | 0.014 | 0.017 | 0.39 |
| Veillonella | rs6656807 | A | G | 0.425 | 0.070 | 0.015 | 5.50E-06 | -0.003 | 0.016 | 0.87 |
| Veillonella | rs7359080 | C | A | 0.127 | -0.135 | 0.030 | 7.40E-06 | 0.012 | 0.030 | 0.69 |
| Veillonella | rs742016 | A | G | 0.424 | -0.069 | 0.015 | 4.66E-06 | -0.017 | 0.015 | 0.24 |
| Veillonella | rs7645873 | A | T | 0.283 | 0.076 | 0.016 | 3.12E-06 | -0.018 | 0.016 | 0.26 |
| Blautia | rs11149971 | C | T | 0.054 | 0.118 | 0.023 | 1.04E-06 | -0.013 | 0.035 | 0.71 |
| Blautia | rs113271346 | C | T | 0.118 | 0.078 | 0.017 | 6.85E-06 | 0.053 | 0.030 | 0.08 |
| Blautia | rs115043014 | G | A | 0.044 | -0.207 | 0.044 | 5.19E-06 | -0.095 | 0.120 | 0.43 |
| Blautia | rs117001700 | T | C | 0.044 | 0.196 | 0.044 | 8.84E-06 | 0.034 | 0.039 | 0.38 |
| Blautia | rs12453000 | C | T | 0.220 | 0.063 | 0.013 | 1.26E-06 | -0.002 | 0.018 | 0.93 |
| Blautia | rs16892041 | T | C | 0.171 | -0.062 | 0.014 | 8.82E-06 | -0.003 | 0.019 | 0.89 |
| Blautia | rs2788271 | T | G | 0.165 | -0.058 | 0.013 | 7.16E-06 | -0.006 | 0.017 | 0.75 |
| Blautia | rs3005511 | A | G | 0.336 | 0.050 | 0.011 | 6.19E-06 | 0.000 | 0.015 | 0.98 |
| Blautia | rs4926264 | T | C | 0.079 | 0.083 | 0.018 | 5.10E-06 | -0.032 | 0.022 | 0.16 |
| Blautia | rs67794373 | C | T | 0.214 | 0.060 | 0.012 | 1.00E-06 | -0.003 | 0.016 | 0.85 |
| Blautia | rs682885 | A | G | 0.417 | -0.049 | 0.011 | 4.49E-06 | 0.013 | 0.015 | 0.36 |
| Blautia | rs72973581 | A | G | 0.055 | 0.125 | 0.027 | 1.74E-06 | -0.011 | 0.033 | 0.75 |
| Blautia | rs7860714 | A | G | 0.334 | -0.050 | 0.011 | 4.09E-06 | -0.026 | 0.015 | 0.08 |
| Dorea | rs11150408 | T | G | 0.445 | 0.049 | 0.011 | 7.06E-06 | 0.008 | 0.015 | 0.58 |
| Dorea | rs12216169 | T | A | 0.084 | 0.088 | 0.019 | 5.33E-06 | 0.031 | 0.027 | 0.25 |
| Dorea | rs12537781 | T | C | 0.230 | -0.056 | 0.013 | 9.15E-06 | -0.016 | 0.017 | 0.33 |
| Dorea | rs13279148 | G | A | 0.166 | 0.072 | 0.015 | 2.25E-06 | 0.007 | 0.022 | 0.75 |
| Dorea | rs1899291 | C | T | 0.128 | 0.070 | 0.015 | 4.57E-06 | -0.031 | 0.020 | 0.12 |
| Dorea | rs3005511 | A | G | 0.336 | 0.052 | 0.011 | 5.29E-06 | 0.000 | 0.015 | 0.98 |
| Dorea | rs345219 | T | G | 0.376 | -0.050 | 0.011 | 8.80E-06 | 0.001 | 0.016 | 0.97 |
| Dorea | rs3752849 | G | A | 0.033 | 0.164 | 0.037 | 7.68E-06 | 0.030 | 0.033 | 0.36 |
| Dorea | rs4793307 | C | T | 0.246 | 0.057 | 0.012 | 4.01E-06 | 0.009 | 0.017 | 0.62 |
| Dorea | rs62503162 | A | G | 0.088 | -0.097 | 0.019 | 7.47E-07 | 0.029 | 0.030 | 0.33 |
| Dorea | rs62583469 | T | A | 0.199 | -0.063 | 0.014 | 5.78E-06 | 0.026 | 0.020 | 0.20 |
| Dorea | rs73729431 | C | T | 0.061 | -0.137 | 0.030 | 3.17E-06 | -0.026 | 0.037 | 0.49 |
| Barnesiella | rs11155559 | T | C | 0.095 | 0.096 | 0.021 | 8.92E-06 | 0.021 | 0.025 | 0.41 |
| Barnesiella | rs113258194 | A | G | 0.102 | 0.099 | 0.021 | 7.31E-06 | -0.033 | 0.034 | 0.34 |
| Barnesiella | rs12909713 | C | T | 0.469 | -0.055 | 0.012 | 4.95E-06 | 0.010 | 0.015 | 0.48 |
| Barnesiella | rs13242616 | T | C | 0.370 | -0.058 | 0.012 | 2.29E-06 | -0.028 | 0.015 | 0.07 |
| Barnesiella | rs199035 | G | A | 0.509 | 0.056 | 0.012 | 3.00E-06 | 0.002 | 0.014 | 0.86 |
| Barnesiella | rs2057922 | G | C | 0.106 | 0.092 | 0.019 | 3.83E-06 | -0.024 | 0.023 | 0.28 |
| Barnesiella | rs2276875 | A | G | 0.255 | -0.070 | 0.014 | 4.65E-07 | -0.010 | 0.017 | 0.55 |
| Barnesiella | rs2428166 | G | A | 0.049 | -0.166 | 0.034 | 8.51E-07 | 0.021 | 0.038 | 0.59 |
| Barnesiella | rs28418786 | C | G | 0.156 | -0.079 | 0.017 | 6.48E-06 | -0.014 | 0.024 | 0.56 |
| Barnesiella | rs35177866 | A | G | 0.124 | 0.092 | 0.019 | 2.95E-06 | 0.013 | 0.026 | 0.61 |
| Barnesiella | rs60316894 | C | T | 0.060 | -0.121 | 0.025 | 1.19E-06 | 0.024 | 0.031 | 0.43 |
| Barnesiella | rs62251337 | A | G | 0.158 | -0.069 | 0.015 | 4.24E-06 | -0.006 | 0.018 | 0.76 |
| Barnesiella | rs72684847 | T | C | 0.066 | -0.114 | 0.025 | 6.76E-06 | 0.042 | 0.032 | 0.19 |
| Barnesiella | rs76181748 | C | T | 0.144 | -0.078 | 0.017 | 6.78E-06 | 0.010 | 0.020 | 0.60 |
| Barnesiella | rs77455852 | T | G | 0.121 | -0.089 | 0.020 | 3.16E-06 | -0.009 | 0.024 | 0.71 |
| Barnesiella | rs79795328 | A | G | 0.125 | -0.082 | 0.018 | 4.23E-06 | -0.005 | 0.023 | 0.83 |
| Desulfovibrio | rs12031543 | T | C | 0.076 | -0.127 | 0.028 | 6.55E-06 | 0.039 | 0.023 | 0.10 |
| Desulfovibrio | rs13066142 | G | A | 0.093 | 0.119 | 0.025 | 3.79E-06 | 0.004 | 0.025 | 0.87 |
| Desulfovibrio | rs16863365 | A | G | 0.117 | 0.109 | 0.023 | 1.79E-06 | -0.005 | 0.025 | 0.85 |
| Desulfovibrio | rs2032031 | A | G | 0.473 | -0.065 | 0.015 | 9.14E-06 | 0.018 | 0.015 | 0.23 |
| Desulfovibrio | rs2590913 | G | A | 0.073 | 0.154 | 0.034 | 6.65E-06 | 0.033 | 0.030 | 0.27 |
| Desulfovibrio | rs2853179 | C | T | 0.249 | 0.081 | 0.017 | 2.42E-06 | 0.013 | 0.016 | 0.44 |
| Desulfovibrio | rs4797774 | G | A | 0.069 | 0.213 | 0.047 | 5.64E-06 | 0.018 | 0.036 | 0.63 |
| Desulfovibrio | rs6580353 | T | C | 0.260 | 0.077 | 0.017 | 4.94E-06 | 0.014 | 0.017 | 0.42 |
| Desulfovibrio | rs72647089 | T | G | 0.106 | -0.107 | 0.024 | 8.30E-06 | -0.037 | 0.025 | 0.15 |
| Desulfovibrio | rs7729080 | C | A | 0.289 | -0.070 | 0.016 | 9.96E-06 | -0.001 | 0.015 | 0.96 |
| Akkermansia | rs11184341 | G | C | 0.278 | 0.066 | 0.014 | 4.06E-06 | 0.005 | 0.015 | 0.74 |
| Akkermansia | rs111862613 | T | C | 0.166 | 0.091 | 0.020 | 3.39E-06 | -0.002 | 0.021 | 0.92 |
| Akkermansia | rs117107102 | A | G | 0.045 | 0.204 | 0.043 | 3.01E-06 | -0.029 | 0.039 | 0.46 |
| Akkermansia | rs11729256 | T | C | 0.239 | 0.075 | 0.015 | 6.58E-07 | -0.017 | 0.016 | 0.31 |
| Akkermansia | rs12908520 | G | A | 0.456 | 0.062 | 0.013 | 2.26E-06 | -0.003 | 0.014 | 0.86 |
| Akkermansia | rs2602429 | C | T | 0.222 | 0.075 | 0.016 | 2.72E-06 | -0.014 | 0.016 | 0.38 |
| Akkermansia | rs4242783 | G | A | 0.287 | 0.069 | 0.015 | 3.00E-06 | 0.015 | 0.016 | 0.34 |
| Akkermansia | rs4936098 | A | G | 0.340 | 0.065 | 0.014 | 1.10E-06 | -0.011 | 0.016 | 0.51 |
| Akkermansia | rs61779207 | G | A | 0.178 | -0.076 | 0.017 | 6.32E-06 | 0.037 | 0.019 | 0.05 |
| Akkermansia | rs74542928 | T | C | 0.079 | 0.113 | 0.024 | 1.48E-06 | -0.016 | 0.028 | 0.56 |
| Akkermansia | rs9349825 | A | G | 0.279 | -0.070 | 0.015 | 2.60E-06 | 0.001 | 0.016 | 0.94 |
| Akkermansia | rs941682 | G | A | 0.311 | -0.063 | 0.014 | 9.17E-06 | 0.021 | 0.016 | 0.19 |
| Eggerthella | rs112205261 | T | C | 0.058 | -0.189 | 0.040 | 3.35E-06 | 0.005 | 0.031 | 0.87 |
| Eggerthella | rs13070736 | A | C | 0.157 | -0.121 | 0.027 | 7.62E-06 | -0.012 | 0.018 | 0.50 |
| Eggerthella | rs1784446 | G | A | 0.442 | 0.091 | 0.020 | 5.23E-06 | -0.005 | 0.014 | 0.71 |
| Eggerthella | rs2223081 | G | A | 0.292 | 0.103 | 0.022 | 3.89E-06 | -0.012 | 0.016 | 0.44 |
| Eggerthella | rs2240838 | A | G | 0.502 | 0.098 | 0.020 | 7.36E-07 | -0.008 | 0.014 | 0.60 |
| Eggerthella | rs2877457 | G | A | 0.868 | -0.093 | 0.021 | 9.03E-06 | -0.001 | 0.020 | 0.95 |
| Eggerthella | rs3851328 | T | G | 0.266 | -0.108 | 0.024 | 4.18E-06 | -0.005 | 0.017 | 0.78 |
| Eggerthella | rs4985746 | G | A | 0.179 | 0.111 | 0.025 | 5.71E-06 | -0.026 | 0.020 | 0.19 |
| Eggerthella | rs6430926 | C | T | 0.514 | 0.088 | 0.020 | 8.37E-06 | 0.017 | 0.014 | 0.22 |
| Eggerthella | rs67490567 | T | C | 0.198 | 0.108 | 0.025 | 8.94E-06 | -0.002 | 0.019 | 0.90 |
| Eggerthella | rs76663501 | C | T | 0.082 | 0.175 | 0.038 | 4.83E-06 | 0.058 | 0.029 | 0.05 |
| Butyricimonas | rs11228830 | A | G | 0.071 | 0.135 | 0.030 | 6.55E-06 | 0.020 | 0.031 | 0.51 |
| Butyricimonas | rs113054641 | G | A | 0.085 | -0.145 | 0.027 | 1.74E-07 | -0.025 | 0.029 | 0.38 |
| Butyricimonas | rs12304031 | G | A | 0.129 | -0.086 | 0.020 | 6.70E-06 | 0.026 | 0.024 | 0.27 |
| Butyricimonas | rs12458763 | A | C | 0.072 | 0.122 | 0.027 | 6.37E-06 | 0.025 | 0.029 | 0.39 |
| Butyricimonas | rs1862649 | G | A | 0.092 | 0.113 | 0.025 | 4.76E-06 | 0.000 | 0.029 | 0.99 |
| Butyricimonas | rs2114713 | G | T | 0.451 | 0.063 | 0.014 | 6.88E-06 | -0.002 | 0.015 | 0.89 |
| Butyricimonas | rs2642760 | G | C | 0.377 | 0.071 | 0.015 | 8.58E-07 | -0.012 | 0.015 | 0.44 |
| Butyricimonas | rs270727 | G | C | 0.322 | -0.069 | 0.015 | 5.38E-06 | -0.012 | 0.016 | 0.44 |
| Butyricimonas | rs326049 | C | G | 0.205 | 0.076 | 0.017 | 8.19E-06 | -0.005 | 0.017 | 0.79 |
| Butyricimonas | rs62130338 | G | A | 0.262 | -0.073 | 0.016 | 3.90E-06 | -0.008 | 0.017 | 0.64 |
| Butyricimonas | rs62390301 | T | C | 0.183 | -0.087 | 0.017 | 7.42E-07 | -0.012 | 0.020 | 0.55 |
| Butyricimonas | rs7083431 | A | C | 0.355 | 0.070 | 0.014 | 8.85E-07 | 0.006 | 0.016 | 0.72 |
| Butyricimonas | rs71428626 | G | T | 0.073 | -0.133 | 0.029 | 4.80E-06 | 0.003 | 0.034 | 0.94 |
| Butyricimonas | rs72814525 | A | G | 0.323 | 0.066 | 0.015 | 8.25E-06 | -0.017 | 0.016 | 0.29 |
| Butyricimonas | rs78453362 | A | G | 0.058 | -0.149 | 0.033 | 4.06E-06 | 0.037 | 0.037 | 0.33 |
| Butyricimonas | rs9657374 | C | T | 0.302 | 0.068 | 0.015 | 4.50E-06 | 0.041 | 0.015 | 0.01 |
| FamilyXIIIUCG001 | rs112362903 | A | G | 0.043 | -0.149 | 0.033 | 7.88E-06 | -0.020 | 0.038 | 0.60 |
| FamilyXIIIUCG001 | rs116979587 | T | A | 0.071 | -0.122 | 0.026 | 3.05E-06 | 0.035 | 0.032 | 0.27 |
| FamilyXIIIUCG001 | rs12049454 | T | C | 0.301 | -0.065 | 0.013 | 1.17E-06 | -0.023 | 0.015 | 0.12 |
| FamilyXIIIUCG001 | rs1426266 | T | C | 0.296 | -0.067 | 0.014 | 1.25E-06 | -0.025 | 0.016 | 0.11 |
| FamilyXIIIUCG001 | rs2276529 | C | G | 0.186 | -0.076 | 0.017 | 5.36E-06 | 0.009 | 0.018 | 0.60 |
| FamilyXIIIUCG001 | rs3842897 | G | A | 0.065 | -0.113 | 0.024 | 5.20E-06 | -0.045 | 0.023 | 0.05 |
| FamilyXIIIUCG001 | rs62414802 | C | T | 0.298 | -0.061 | 0.013 | 4.29E-06 | -0.036 | 0.017 | 0.03 |
| FamilyXIIIUCG001 | rs7119679 | G | A | 0.150 | -0.081 | 0.017 | 3.52E-06 | 0.041 | 0.018 | 0.03 |
| FamilyXIIIUCG001 | rs76463770 | A | G | 0.052 | 0.193 | 0.042 | 3.77E-06 | 0.018 | 0.036 | 0.61 |
| FamilyXIIIUCG001 | rs8076666 | A | G | 0.136 | 0.089 | 0.020 | 8.02E-06 | 0.028 | 0.025 | 0.27 |
| RuminococcaceaeNK4A214group | rs11241747 | C | T | 0.391 | 0.053 | 0.012 | 6.59E-06 | -0.004 | 0.017 | 0.80 |
| RuminococcaceaeNK4A214group | rs114244418 | C | G | 0.041 | -0.175 | 0.037 | 3.59E-06 | -0.081 | 0.037 | 0.03 |
| RuminococcaceaeNK4A214group | rs11586410 | G | A | 0.134 | -0.086 | 0.017 | 3.66E-07 | 0.020 | 0.020 | 0.31 |
| RuminococcaceaeNK4A214group | rs12642039 | T | C | 0.366 | -0.055 | 0.012 | 3.43E-06 | -0.008 | 0.015 | 0.57 |
| RuminococcaceaeNK4A214group | rs12731 | A | G | 0.451 | -0.053 | 0.012 | 4.87E-06 | 0.006 | 0.014 | 0.67 |
| RuminococcaceaeNK4A214group | rs13087692 | T | G | 0.305 | 0.057 | 0.013 | 8.69E-06 | -0.001 | 0.017 | 0.93 |
| RuminococcaceaeNK4A214group | rs136761 | G | A | 0.429 | -0.059 | 0.012 | 8.15E-07 | -0.026 | 0.015 | 0.07 |
| RuminococcaceaeNK4A214group | rs147475196 | A | G | 0.078 | -0.134 | 0.030 | 4.72E-06 | -0.008 | 0.032 | 0.80 |
| RuminococcaceaeNK4A214group | rs34576931 | G | C | 0.092 | -0.087 | 0.019 | 4.72E-06 | -0.008 | 0.026 | 0.75 |
| RuminococcaceaeNK4A214group | rs35559912 | T | C | 0.066 | -0.093 | 0.020 | 4.89E-06 | 0.018 | 0.028 | 0.52 |
| RuminococcaceaeNK4A214group | rs4814689 | C | T | 0.067 | -0.108 | 0.023 | 4.55E-06 | -0.001 | 0.025 | 0.97 |
| RuminococcaceaeNK4A214group | rs5994253 | A | G | 0.146 | -0.081 | 0.016 | 2.35E-07 | 0.002 | 0.018 | 0.94 |
| RuminococcaceaeNK4A214group | rs62027366 | T | C | 0.230 | 0.062 | 0.014 | 6.58E-06 | 0.017 | 0.017 | 0.32 |
| RuminococcaceaeNK4A214group | rs6681678 | C | T | 0.050 | -0.100 | 0.024 | 9.05E-06 | -0.024 | 0.031 | 0.44 |
| RuminococcaceaeNK4A214group | rs73158814 | C | G | 0.074 | -0.109 | 0.023 | 2.20E-06 | 0.019 | 0.033 | 0.56 |
| RuminococcaceaeNK4A214group | rs7573569 | T | C | 0.072 | 0.108 | 0.023 | 3.23E-06 | -0.030 | 0.031 | 0.33 |
| Eubacteriumbrachygroup | rs112617308 | T | C | 0.080 | -0.171 | 0.036 | 2.38E-06 | 0.026 | 0.024 | 0.29 |
| Eubacteriumbrachygroup | rs12151423 | A | G | 0.479 | 0.101 | 0.023 | 9.27E-06 | 0.003 | 0.014 | 0.82 |
| Eubacteriumbrachygroup | rs13139592 | T | C | 0.122 | -0.146 | 0.033 | 7.97E-06 | 0.024 | 0.020 | 0.23 |
| Eubacteriumbrachygroup | rs1384962 | A | G | 0.211 | 0.121 | 0.027 | 6.99E-06 | -0.020 | 0.017 | 0.25 |
| Eubacteriumbrachygroup | rs2913110 | C | T | 0.378 | 0.105 | 0.023 | 4.56E-06 | 0.005 | 0.016 | 0.73 |
| Eubacteriumbrachygroup | rs4862235 | G | A | 0.444 | 0.105 | 0.023 | 3.73E-06 | 0.018 | 0.014 | 0.20 |
| Eubacteriumbrachygroup | rs62348779 | T | C | 0.082 | -0.201 | 0.043 | 3.78E-06 | -0.060 | 0.030 | 0.04 |
| Eubacteriumbrachygroup | rs6591893 | G | A | 0.348 | 0.108 | 0.024 | 7.34E-06 | 0.021 | 0.015 | 0.17 |
| Eubacteriumbrachygroup | rs720439 | A | G | 0.252 | -0.112 | 0.025 | 7.03E-06 | 0.017 | 0.017 | 0.31 |
| Eubacteriumbrachygroup | rs73199919 | T | C | 0.057 | -0.237 | 0.053 | 8.16E-06 | -0.026 | 0.033 | 0.43 |
| Eubacteriumbrachygroup | rs9613196 | T | A | 0.077 | -0.239 | 0.053 | 4.99E-06 | -0.009 | 0.034 | 0.78 |
| Clostridiumsensustricto1 | rs11264403 | G | A | 0.040 | -0.139 | 0.033 | 7.76E-06 | -0.012 | 0.025 | 0.65 |
| Clostridiumsensustricto1 | rs115807074 | A | G | 0.050 | -0.227 | 0.049 | 4.32E-06 | 0.015 | 0.042 | 0.73 |
| Clostridiumsensustricto1 | rs11586026 | A | T | 0.064 | 0.111 | 0.025 | 8.85E-06 | 0.013 | 0.031 | 0.67 |
| Clostridiumsensustricto1 | rs116847295 | C | T | 0.076 | 0.110 | 0.025 | 4.58E-06 | 0.011 | 0.030 | 0.71 |
| Clostridiumsensustricto1 | rs12341505 | G | A | 0.111 | 0.081 | 0.018 | 4.82E-06 | 0.003 | 0.022 | 0.89 |
| Clostridiumsensustricto1 | rs12490337 | C | G | 0.246 | -0.062 | 0.014 | 7.49E-06 | -0.020 | 0.016 | 0.22 |
| Clostridiumsensustricto1 | rs2795528 | G | A | 0.049 | -0.184 | 0.039 | 2.72E-06 | 0.014 | 0.034 | 0.69 |
| Clostridiumsensustricto1 | rs2817172 | C | T | 0.413 | 0.058 | 0.012 | 2.77E-06 | -0.011 | 0.015 | 0.46 |
| Clostridiumsensustricto1 | rs550843 | T | C | 0.149 | -0.078 | 0.017 | 2.05E-06 | -0.016 | 0.019 | 0.38 |
| Escherichia.Shigella | rs112767262 | T | C | 0.196 | 0.073 | 0.016 | 8.21E-06 | 0.027 | 0.020 | 0.18 |
| Escherichia.Shigella | rs113127095 | A | G | 0.052 | 0.151 | 0.032 | 3.33E-06 | 0.013 | 0.034 | 0.69 |
| Escherichia.Shigella | rs113513883 | A | G | 0.058 | 0.172 | 0.038 | 5.28E-06 | -0.014 | 0.036 | 0.70 |
| Escherichia.Shigella | rs1154904 | A | G | 0.464 | -0.061 | 0.013 | 3.04E-06 | -0.004 | 0.014 | 0.77 |
| Escherichia.Shigella | rs11706043 | T | A | 0.157 | 0.076 | 0.016 | 5.87E-06 | 0.011 | 0.018 | 0.55 |
| Escherichia.Shigella | rs117092367 | A | T | 0.089 | 0.117 | 0.026 | 9.65E-06 | 0.044 | 0.030 | 0.14 |
| Escherichia.Shigella | rs118526 | C | A | 0.393 | -0.059 | 0.014 | 8.00E-06 | -0.025 | 0.016 | 0.13 |
| Escherichia.Shigella | rs2267739 | G | C | 0.086 | 0.116 | 0.024 | 1.42E-06 | 0.005 | 0.027 | 0.85 |
| Escherichia.Shigella | rs2798105 | A | G | 0.118 | -0.101 | 0.022 | 8.25E-06 | -0.053 | 0.026 | 0.04 |
| Escherichia.Shigella | rs35555519 | C | G | 0.114 | 0.102 | 0.022 | 4.92E-06 | 0.033 | 0.023 | 0.14 |
| Escherichia.Shigella | rs4731451 | G | A | 0.372 | -0.061 | 0.014 | 7.47E-06 | -0.024 | 0.015 | 0.11 |
| Escherichia.Shigella | rs57024273 | T | C | 0.318 | 0.063 | 0.014 | 9.70E-06 | 0.017 | 0.016 | 0.29 |
| Escherichia.Shigella | rs592299 | T | C | 0.468 | -0.059 | 0.013 | 4.77E-06 | -0.017 | 0.014 | 0.24 |
| Escherichia.Shigella | rs73208162 | A | G | 0.059 | -0.119 | 0.025 | 2.19E-06 | -0.018 | 0.031 | 0.58 |
| Escherichia.Shigella | rs7502686 | G | C | 0.063 | -0.136 | 0.030 | 5.90E-06 | 0.013 | 0.031 | 0.69 |
| DefluviitaleaceaeUCG011 | rs112893842 | T | C | 0.097 | 0.114 | 0.023 | 1.45E-06 | 0.003 | 0.020 | 0.90 |
| DefluviitaleaceaeUCG011 | rs1582238 | T | C | 0.372 | 0.081 | 0.017 | 1.57E-06 | 0.015 | 0.016 | 0.34 |
| DefluviitaleaceaeUCG011 | rs28696126 | A | T | 0.192 | -0.107 | 0.024 | 6.63E-06 | -0.035 | 0.024 | 0.14 |
| DefluviitaleaceaeUCG011 | rs2892880 | G | A | 0.260 | 0.082 | 0.018 | 6.83E-06 | -0.013 | 0.016 | 0.43 |
| DefluviitaleaceaeUCG011 | rs4344384 | G | T | 0.474 | 0.072 | 0.016 | 4.83E-06 | 0.007 | 0.014 | 0.60 |
| DefluviitaleaceaeUCG011 | rs4677103 | A | G | 0.171 | 0.098 | 0.020 | 9.60E-07 | -0.015 | 0.018 | 0.40 |
| DefluviitaleaceaeUCG011 | rs55658617 | T | C | 0.070 | 0.174 | 0.036 | 2.15E-06 | -0.058 | 0.031 | 0.06 |
| DefluviitaleaceaeUCG011 | rs72731813 | C | T | 0.082 | -0.147 | 0.029 | 4.33E-07 | 0.045 | 0.030 | 0.14 |
| DefluviitaleaceaeUCG011 | rs9608282 | T | G | 0.066 | 0.143 | 0.030 | 2.52E-06 | 0.026 | 0.028 | 0.36 |
| DefluviitaleaceaeUCG011 | rs9725395 | A | G | 0.088 | -0.138 | 0.030 | 3.52E-06 | 0.078 | 0.028 | 0.01 |
| RuminococcaceaeUCG009 | rs113006825 | T | C | 0.180 | -0.093 | 0.021 | 7.98E-06 | -0.017 | 0.018 | 0.35 |
| RuminococcaceaeUCG009 | rs12508214 | C | T | 0.351 | -0.077 | 0.017 | 4.75E-06 | -0.007 | 0.015 | 0.63 |
| RuminococcaceaeUCG009 | rs138460696 | A | G | 0.106 | 0.139 | 0.032 | 9.81E-06 | -0.045 | 0.030 | 0.13 |
| RuminococcaceaeUCG009 | rs1550196 | G | A | 0.117 | 0.131 | 0.026 | 1.13E-06 | 0.028 | 0.021 | 0.17 |
| RuminococcaceaeUCG009 | rs2058609 | A | G | 0.314 | 0.082 | 0.017 | 3.12E-06 | 0.008 | 0.015 | 0.62 |
| RuminococcaceaeUCG009 | rs2192926 | A | G | 0.237 | -0.089 | 0.019 | 4.88E-06 | 0.001 | 0.016 | 0.93 |
| RuminococcaceaeUCG009 | rs4079028 | C | T | 0.194 | 0.092 | 0.020 | 3.28E-06 | 0.012 | 0.018 | 0.50 |
| RuminococcaceaeUCG009 | rs4708333 | T | G | 0.302 | -0.084 | 0.017 | 1.56E-06 | 0.005 | 0.015 | 0.77 |
| RuminococcaceaeUCG009 | rs61779334 | G | C | 0.104 | -0.138 | 0.029 | 1.94E-06 | -0.005 | 0.027 | 0.86 |
| RuminococcaceaeUCG009 | rs6952765 | G | A | 0.461 | 0.073 | 0.017 | 8.13E-06 | -0.023 | 0.015 | 0.14 |
| RuminococcaceaeUCG009 | rs758191 | T | G | 0.064 | 0.177 | 0.038 | 9.01E-06 | -0.047 | 0.033 | 0.16 |
| RuminococcaceaeUCG009 | rs78410648 | A | G | 0.097 | 0.121 | 0.028 | 9.67E-06 | -0.005 | 0.022 | 0.82 |
| RuminococcaceaeUCG009 | rs8009993 | G | C | 0.129 | -0.136 | 0.024 | 4.42E-08 | 0.013 | 0.025 | 0.59 |
| RuminococcaceaeUCG009 | rs9558661 | T | C | 0.187 | -0.090 | 0.020 | 7.01E-06 | 0.013 | 0.017 | 0.45 |
| Ruminiclostridium9 | rs113048721 | C | G | 0.239 | 0.060 | 0.013 | 4.10E-06 | 0.006 | 0.018 | 0.73 |
| Ruminiclostridium9 | rs115044523 | G | A | 0.092 | -0.098 | 0.020 | 2.37E-06 | -0.013 | 0.033 | 0.69 |
| Ruminiclostridium9 | rs12040548 | G | T | 0.311 | 0.057 | 0.012 | 3.15E-06 | 0.022 | 0.017 | 0.20 |
| Ruminiclostridium9 | rs12419854 | T | A | 0.178 | -0.073 | 0.016 | 3.18E-06 | 0.006 | 0.021 | 0.79 |
| Ruminiclostridium9 | rs57665991 | C | G | 0.239 | -0.064 | 0.012 | 2.07E-07 | -0.013 | 0.016 | 0.43 |
| Ruminiclostridium9 | rs6082461 | A | C | 0.207 | 0.059 | 0.013 | 4.87E-06 | 0.006 | 0.017 | 0.72 |
| Ruminiclostridium9 | rs7137760 | C | T | 0.415 | 0.051 | 0.011 | 7.07E-06 | 0.012 | 0.015 | 0.43 |
| Ruminiclostridium9 | rs73592673 | A | T | 0.147 | -0.082 | 0.017 | 2.14E-06 | -0.036 | 0.022 | 0.10 |
| Ruminiclostridium9 | rs74303178 | T | C | 0.320 | 0.053 | 0.012 | 7.92E-06 | 0.007 | 0.015 | 0.66 |
| Ruminiclostridium9 | rs78191726 | T | C | 0.091 | 0.094 | 0.021 | 7.58E-06 | -0.051 | 0.028 | 0.07 |
| Ruminiclostridium9 | rs79082720 | C | G | 0.095 | 0.093 | 0.020 | 6.47E-06 | 0.007 | 0.026 | 0.78 |
| Ruminiclostridium9 | rs918449 | A | G | 0.070 | -0.095 | 0.020 | 2.56E-06 | -0.020 | 0.022 | 0.37 |
| Ruminiclostridium9 | rs9522712 | T | C | 0.145 | 0.070 | 0.015 | 4.66E-06 | -0.040 | 0.019 | 0.04 |
| Ruminiclostridium9 | rs9809789 | C | T | 0.168 | -0.072 | 0.016 | 8.72E-06 | -0.032 | 0.022 | 0.14 |
| Sellimonas | rs113379006 | T | C | 0.168 | -0.163 | 0.036 | 7.21E-06 | -0.021 | 0.022 | 0.35 |
| Sellimonas | rs13417181 | T | C | 0.216 | 0.167 | 0.034 | 7.62E-07 | 0.007 | 0.039 | 0.85 |
| Sellimonas | rs2016057 | A | C | 0.391 | -0.126 | 0.026 | 1.03E-06 | 0.017 | 0.016 | 0.30 |
| Sellimonas | rs2187447 | A | C | 0.061 | 0.243 | 0.053 | 3.98E-06 | 0.027 | 0.032 | 0.39 |
| Sellimonas | rs2371572 | A | C | 0.549 | 0.127 | 0.025 | 4.46E-07 | 0.004 | 0.015 | 0.79 |
| Sellimonas | rs41816 | A | G | 0.252 | 0.132 | 0.029 | 8.39E-06 | -0.002 | 0.016 | 0.89 |
| Sellimonas | rs4600608 | A | G | 0.239 | -0.137 | 0.030 | 4.95E-06 | -0.018 | 0.017 | 0.29 |
| Sellimonas | rs553697 | T | C | 0.162 | -0.154 | 0.034 | 6.13E-06 | -0.029 | 0.019 | 0.14 |
| Sellimonas | rs56203279 | T | C | 0.330 | -0.124 | 0.027 | 3.72E-06 | 0.004 | 0.015 | 0.82 |
| Sellimonas | rs72553859 | G | C | 0.175 | -0.150 | 0.033 | 5.38E-06 | 0.001 | 0.019 | 0.95 |
| Sellimonas | rs7968030 | A | T | 0.283 | -0.127 | 0.028 | 5.56E-06 | 0.009 | 0.020 | 0.65 |
| Parabacteroides | rs114567323 | T | C | 0.047 | 0.186 | 0.041 | 5.65E-06 | -0.113 | 0.044 | 0.01 |
| Parabacteroides | rs115602804 | G | A | 0.067 | 0.103 | 0.022 | 1.93E-06 | -0.054 | 0.032 | 0.09 |
| Parabacteroides | rs11965579 | G | C | 0.035 | 0.163 | 0.038 | 8.87E-06 | 0.005 | 0.028 | 0.86 |
| Parabacteroides | rs3860755 | G | C | 0.308 | 0.056 | 0.012 | 1.71E-06 | 0.002 | 0.015 | 0.89 |
| Parabacteroides | rs4236095 | G | A | 0.125 | 0.076 | 0.016 | 1.93E-06 | -0.023 | 0.022 | 0.29 |
| Parabacteroides | rs60884758 | C | T | 0.165 | -0.070 | 0.014 | 5.71E-07 | -0.015 | 0.018 | 0.40 |
| Parabacteroides | rs6657302 | T | C | 0.052 | -0.105 | 0.023 | 9.76E-06 | 0.005 | 0.030 | 0.87 |
| Parabacteroides | rs72893646 | A | T | 0.128 | -0.072 | 0.016 | 8.83E-06 | 0.010 | 0.022 | 0.64 |
| Parabacteroides | rs7298818 | C | T | 0.077 | 0.089 | 0.020 | 8.54E-06 | -0.034 | 0.028 | 0.21 |
| Sutterella | rs1145877 | A | G | 0.159 | -0.074 | 0.016 | 7.20E-06 | 0.020 | 0.019 | 0.29 |
| Sutterella | rs11591622 | T | G | 0.216 | -0.069 | 0.015 | 6.50E-06 | -0.006 | 0.019 | 0.77 |
| Sutterella | rs13173038 | A | G | 0.221 | -0.072 | 0.015 | 2.73E-06 | -0.029 | 0.017 | 0.09 |
| Sutterella | rs143438747 | T | C | 0.055 | -0.146 | 0.031 | 3.28E-06 | -0.053 | 0.032 | 0.10 |
| Sutterella | rs2050185 | G | A | 0.378 | 0.058 | 0.013 | 7.97E-06 | -0.002 | 0.015 | 0.88 |
| Sutterella | rs2321387 | G | A | 0.530 | -0.059 | 0.012 | 1.87E-06 | -0.003 | 0.015 | 0.83 |
| Sutterella | rs2613606 | C | T | 0.394 | -0.056 | 0.012 | 7.20E-06 | 0.018 | 0.016 | 0.25 |
| Sutterella | rs607327 | C | T | 0.377 | 0.058 | 0.013 | 6.63E-06 | 0.022 | 0.015 | 0.15 |
| Sutterella | rs62501473 | G | A | 0.229 | 0.069 | 0.015 | 5.52E-06 | -0.022 | 0.018 | 0.22 |
| Sutterella | rs7499539 | A | G | 0.323 | 0.062 | 0.013 | 2.36E-06 | -0.009 | 0.015 | 0.57 |
| Sutterella | rs7638039 | T | C | 0.233 | 0.065 | 0.014 | 8.66E-06 | -0.001 | 0.017 | 0.97 |
| Sutterella | rs9350083 | T | G | 0.299 | -0.059 | 0.013 | 8.23E-06 | 0.006 | 0.016 | 0.73 |
| Flavonifractor | rs114873521 | C | T | 0.077 | -0.130 | 0.029 | 7.13E-06 | -0.063 | 0.032 | 0.05 |
| Flavonifractor | rs11642826 | G | C | 0.064 | 0.147 | 0.033 | 6.65E-06 | -0.026 | 0.033 | 0.43 |
| Flavonifractor | rs11811696 | T | C | 0.110 | -0.116 | 0.024 | 2.07E-06 | -0.018 | 0.027 | 0.51 |
| Flavonifractor | rs12030302 | A | G | 0.491 | -0.069 | 0.014 | 5.61E-07 | -0.016 | 0.015 | 0.28 |
| Flavonifractor | rs12038887 | C | G | 0.109 | 0.094 | 0.021 | 9.37E-06 | 0.005 | 0.019 | 0.79 |
| Flavonifractor | rs34066017 | A | G | 0.246 | 0.076 | 0.016 | 1.52E-06 | -0.009 | 0.018 | 0.61 |
| Flavonifractor | rs6761463 | G | C | 0.165 | -0.083 | 0.018 | 8.11E-06 | 0.022 | 0.020 | 0.26 |
| Flavonifractor | rs806808 | T | C | 0.476 | 0.067 | 0.014 | 1.18E-06 | -0.023 | 0.014 | 0.10 |
| Coprobacter | rs11532348 | C | T | 0.183 | -0.104 | 0.023 | 5.71E-06 | -0.037 | 0.019 | 0.05 |
| Coprobacter | rs12684609 | T | C | 0.198 | 0.101 | 0.022 | 6.10E-06 | -0.006 | 0.018 | 0.75 |
| Coprobacter | rs12996055 | A | C | 0.192 | 0.092 | 0.021 | 8.08E-06 | -0.019 | 0.018 | 0.29 |
| Coprobacter | rs143662916 | C | T | 0.058 | 0.253 | 0.054 | 3.07E-06 | -0.015 | 0.036 | 0.67 |
| Coprobacter | rs189356 | G | A | 0.522 | 0.078 | 0.017 | 6.26E-06 | -0.006 | 0.016 | 0.71 |
| Coprobacter | rs213863 | C | T | 0.309 | -0.089 | 0.019 | 2.35E-06 | -0.003 | 0.015 | 0.87 |
| Coprobacter | rs28402691 | T | C | 0.167 | 0.111 | 0.025 | 9.56E-06 | 0.015 | 0.021 | 0.47 |
| Coprobacter | rs305411 | A | G | 0.135 | 0.129 | 0.026 | 1.01E-06 | -0.005 | 0.021 | 0.83 |
| Coprobacter | rs3828477 | G | T | 0.281 | -0.091 | 0.020 | 2.89E-06 | 0.023 | 0.016 | 0.15 |
| Coprobacter | rs5011652 | G | C | 0.246 | 0.090 | 0.020 | 5.51E-06 | 0.000 | 0.016 | 0.99 |
| Coprobacter | rs55672356 | T | A | 0.053 | -0.193 | 0.041 | 2.74E-06 | -0.010 | 0.032 | 0.76 |
| Coprobacter | rs72821405 | T | C | 0.089 | -0.147 | 0.032 | 4.76E-06 | -0.022 | 0.028 | 0.44 |
| Coprobacter | rs74919520 | G | A | 0.128 | 0.126 | 0.028 | 5.76E-06 | -0.020 | 0.023 | 0.38 |
| Coprobacter | rs76001613 | C | G | 0.070 | 0.216 | 0.049 | 9.13E-06 | -0.039 | 0.031 | 0.21 |
| RuminococcaceaeUCG013 | rs11581881 | C | T | 0.181 | 0.066 | 0.014 | 4.73E-06 | -0.001 | 0.020 | 0.96 |
| RuminococcaceaeUCG013 | rs12189346 | G | A | 0.163 | 0.068 | 0.015 | 1.68E-06 | 0.007 | 0.018 | 0.71 |
| RuminococcaceaeUCG013 | rs12336782 | T | C | 0.082 | -0.086 | 0.019 | 8.60E-06 | 0.004 | 0.025 | 0.88 |
| RuminococcaceaeUCG013 | rs12485353 | G | A | 0.179 | -0.061 | 0.013 | 4.19E-06 | -0.004 | 0.016 | 0.81 |
| RuminococcaceaeUCG013 | rs12781711 | C | T | 0.313 | -0.066 | 0.012 | 2.55E-08 | -0.036 | 0.016 | 0.03 |
| RuminococcaceaeUCG013 | rs16918863 | A | C | 0.061 | 0.111 | 0.024 | 4.16E-06 | -0.042 | 0.027 | 0.12 |
| RuminococcaceaeUCG013 | rs1729063 | G | C | 0.328 | -0.053 | 0.012 | 9.64E-06 | 0.008 | 0.017 | 0.66 |
| RuminococcaceaeUCG013 | rs2730183 | G | A | 0.464 | -0.049 | 0.011 | 8.44E-06 | 0.026 | 0.015 | 0.07 |
| RuminococcaceaeUCG013 | rs4385846 | G | T | 0.207 | 0.060 | 0.013 | 6.46E-06 | -0.010 | 0.017 | 0.53 |
| RuminococcaceaeUCG013 | rs75088940 | T | C | 0.093 | -0.094 | 0.020 | 2.55E-06 | -0.016 | 0.029 | 0.57 |
| RuminococcaceaeUCG013 | rs76973485 | G | T | 0.037 | 0.195 | 0.042 | 3.35E-06 | 0.008 | 0.035 | 0.81 |
| RuminococcaceaeUCG013 | rs7784330 | G | A | 0.389 | -0.050 | 0.011 | 8.16E-06 | -0.016 | 0.016 | 0.32 |
| RuminococcaceaeUCG013 | rs9313055 | T | C | 0.069 | 0.105 | 0.023 | 9.55E-06 | -0.024 | 0.029 | 0.41 |
| RuminococcaceaeUCG013 | rs9565219 | T | A | 0.337 | -0.052 | 0.012 | 8.73E-06 | 0.016 | 0.015 | 0.30 |
| Bacteroides | rs11585893 | A | G | 0.148 | -0.074 | 0.015 | 1.80E-06 | 0.012 | 0.018 | 0.52 |
| Bacteroides | rs13207588 | A | G | 0.233 | -0.059 | 0.013 | 7.49E-06 | 0.031 | 0.020 | 0.11 |
| Bacteroides | rs1340391 | T | C | 0.192 | -0.059 | 0.013 | 6.73E-06 | 0.002 | 0.019 | 0.94 |
| Bacteroides | rs17619981 | T | G | 0.066 | 0.088 | 0.019 | 2.69E-06 | -0.012 | 0.024 | 0.63 |
| Bacteroides | rs2023437 | T | C | 0.124 | -0.078 | 0.017 | 5.02E-06 | 0.006 | 0.025 | 0.81 |
| Bacteroides | rs2366421 | T | A | 0.234 | -0.053 | 0.012 | 7.65E-06 | 0.003 | 0.017 | 0.87 |
| Bacteroides | rs28757219 | T | A | 0.152 | 0.082 | 0.017 | 1.29E-06 | 0.010 | 0.044 | 0.82 |
| Bacteroides | rs495004 | C | G | 0.210 | -0.061 | 0.013 | 3.42E-06 | 0.006 | 0.017 | 0.73 |
| Bacteroides | rs66474973 | G | T | 0.115 | 0.081 | 0.016 | 6.81E-07 | -0.021 | 0.028 | 0.46 |
| Bacteroides | rs66710942 | C | T | 0.416 | 0.049 | 0.011 | 5.86E-06 | -0.022 | 0.015 | 0.13 |
| Bacteroides | rs6795673 | C | T | 0.412 | 0.054 | 0.011 | 3.38E-07 | -0.011 | 0.014 | 0.42 |
| Bacteroides | rs9507307 | C | T | 0.190 | 0.060 | 0.013 | 2.13E-06 | 0.017 | 0.020 | 0.40 |
| Ruminococcusgnavusgroup | rs11597105 | A | G | 0.112 | 0.115 | 0.025 | 6.95E-06 | -0.004 | 0.020 | 0.84 |
| Ruminococcusgnavusgroup | rs11864644 | T | C | 0.071 | -0.140 | 0.032 | 5.01E-06 | 0.047 | 0.023 | 0.04 |
| Ruminococcusgnavusgroup | rs12136548 | C | T | 0.291 | 0.090 | 0.020 | 3.10E-06 | 0.023 | 0.016 | 0.16 |
| Ruminococcusgnavusgroup | rs12989336 | G | A | 0.305 | -0.085 | 0.019 | 7.12E-06 | -0.002 | 0.016 | 0.88 |
| Ruminococcusgnavusgroup | rs13163520 | G | A | 0.181 | -0.127 | 0.023 | 5.61E-08 | -0.017 | 0.018 | 0.37 |
| Ruminococcusgnavusgroup | rs2909242 | C | A | 0.386 | -0.091 | 0.018 | 7.41E-07 | 0.006 | 0.015 | 0.69 |
| Ruminococcusgnavusgroup | rs3124783 | A | G | 0.120 | -0.116 | 0.025 | 2.67E-06 | -0.006 | 0.019 | 0.76 |
| Ruminococcusgnavusgroup | rs4388134 | C | T | 0.246 | -0.090 | 0.020 | 9.12E-06 | -0.008 | 0.016 | 0.61 |
| Ruminococcusgnavusgroup | rs62167033 | T | C | 0.071 | 0.185 | 0.040 | 3.50E-06 | -0.014 | 0.033 | 0.66 |
| Ruminococcusgnavusgroup | rs78399089 | T | C | 0.083 | 0.144 | 0.033 | 6.63E-06 | -0.054 | 0.027 | 0.05 |
| Ruminococcusgnavusgroup | rs934940 | A | C | 0.185 | -0.105 | 0.023 | 2.74E-06 | -0.054 | 0.020 | 0.01 |
| Ruminococcusgnavusgroup | rs9872758 | T | C | 0.477 | 0.085 | 0.018 | 1.66E-06 | -0.016 | 0.016 | 0.32 |
| Eubacteriumventriosumgroup | rs11617697 | A | G | 0.048 | -0.143 | 0.029 | 7.22E-07 | -0.031 | 0.036 | 0.40 |
| Eubacteriumventriosumgroup | rs12964517 | G | A | 0.316 | 0.059 | 0.012 | 2.07E-06 | 0.001 | 0.016 | 0.96 |
| Eubacteriumventriosumgroup | rs13082419 | C | T | 0.170 | -0.072 | 0.016 | 9.56E-06 | 0.006 | 0.022 | 0.78 |
| Eubacteriumventriosumgroup | rs16884680 | G | T | 0.107 | -0.091 | 0.019 | 1.74E-06 | 0.009 | 0.023 | 0.71 |
| Eubacteriumventriosumgroup | rs35179274 | C | T | 0.244 | -0.063 | 0.014 | 5.76E-06 | -0.021 | 0.018 | 0.24 |
| Eubacteriumventriosumgroup | rs3809430 | T | C | 0.348 | -0.055 | 0.012 | 3.55E-06 | -0.020 | 0.015 | 0.17 |
| Eubacteriumventriosumgroup | rs57199565 | T | C | 0.241 | 0.078 | 0.016 | 7.97E-07 | 0.070 | 0.042 | 0.10 |
| Eubacteriumventriosumgroup | rs66746423 | C | T | 0.156 | 0.075 | 0.016 | 6.11E-06 | 0.005 | 0.022 | 0.83 |
| Eubacteriumventriosumgroup | rs6704822 | A | G | 0.116 | 0.074 | 0.017 | 6.62E-06 | -0.025 | 0.021 | 0.22 |
| Eubacteriumventriosumgroup | rs72783037 | C | A | 0.187 | 0.066 | 0.014 | 6.55E-06 | -0.020 | 0.020 | 0.30 |
| Eubacteriumventriosumgroup | rs73615400 | T | C | 0.091 | -0.096 | 0.019 | 9.54E-07 | -0.034 | 0.022 | 0.11 |
| Eubacteriumventriosumgroup | rs73849225 | T | C | 0.080 | 0.098 | 0.022 | 5.21E-06 | 0.005 | 0.031 | 0.88 |
| Eubacteriumventriosumgroup | rs78250280 | G | A | 0.149 | 0.075 | 0.016 | 3.36E-06 | -0.003 | 0.022 | 0.90 |
| Eubacteriumventriosumgroup | rs876734 | C | T | 0.289 | -0.062 | 0.013 | 2.89E-06 | -0.014 | 0.016 | 0.38 |
| Eubacteriumventriosumgroup | rs9316536 | T | G | 0.136 | -0.082 | 0.018 | 7.84E-06 | 0.014 | 0.021 | 0.53 |
| Marvinbryantia | rs11620597 | T | C | 0.053 | 0.119 | 0.027 | 7.80E-06 | -0.035 | 0.035 | 0.30 |
| Marvinbryantia | rs1187983 | C | T | 0.122 | -0.094 | 0.019 | 2.02E-06 | 0.014 | 0.020 | 0.47 |
| Marvinbryantia | rs146541147 | G | A | 0.071 | 0.119 | 0.027 | 6.86E-06 | 0.046 | 0.034 | 0.18 |
| Marvinbryantia | rs2724813 | A | G | 0.201 | -0.084 | 0.017 | 6.28E-07 | -0.004 | 0.019 | 0.85 |
| Marvinbryantia | rs2842896 | C | T | 0.528 | -0.065 | 0.013 | 7.25E-07 | 0.008 | 0.015 | 0.61 |
| Marvinbryantia | rs2863363 | A | G | 0.368 | 0.063 | 0.014 | 3.11E-06 | -0.012 | 0.015 | 0.44 |
| Marvinbryantia | rs3125832 | A | C | 0.262 | 0.068 | 0.015 | 5.03E-06 | -0.003 | 0.016 | 0.86 |
| Marvinbryantia | rs61884471 | G | A | 0.085 | 0.124 | 0.025 | 1.01E-06 | -0.025 | 0.029 | 0.39 |
| Marvinbryantia | rs72948274 | A | C | 0.058 | -0.126 | 0.027 | 3.26E-06 | -0.037 | 0.033 | 0.27 |
| Marvinbryantia | rs8006832 | G | T | 0.111 | -0.095 | 0.022 | 6.58E-06 | 0.007 | 0.024 | 0.77 |
| Roseburia | rs116270582 | T | A | 0.049 | -0.154 | 0.033 | 1.20E-06 | 0.015 | 0.034 | 0.66 |
| Roseburia | rs12740451 | T | C | 0.125 | 0.070 | 0.015 | 7.34E-06 | 0.002 | 0.022 | 0.94 |
| Roseburia | rs16910295 | T | C | 0.061 | -0.098 | 0.021 | 2.91E-06 | -0.019 | 0.026 | 0.46 |
| Roseburia | rs2034589 | G | C | 0.228 | 0.063 | 0.012 | 5.01E-07 | 0.011 | 0.017 | 0.53 |
| Roseburia | rs2160994 | T | C | 0.380 | 0.055 | 0.011 | 9.70E-07 | -0.028 | 0.016 | 0.09 |
| Roseburia | rs2943022 | T | C | 0.460 | 0.049 | 0.011 | 4.11E-06 | -0.008 | 0.015 | 0.60 |
| Roseburia | rs302266 | T | C | 0.120 | -0.078 | 0.017 | 8.13E-06 | 0.031 | 0.024 | 0.21 |
| Roseburia | rs329182 | T | C | 0.157 | 0.069 | 0.015 | 5.90E-06 | 0.007 | 0.019 | 0.73 |
| Roseburia | rs55858165 | A | C | 0.078 | 0.179 | 0.040 | 9.99E-06 | -0.085 | 0.036 | 0.02 |
| Roseburia | rs57466170 | C | T | 0.087 | 0.074 | 0.017 | 8.30E-06 | -0.001 | 0.022 | 0.96 |
| Roseburia | rs6445851 | G | A | 0.401 | -0.050 | 0.011 | 3.53E-06 | 0.016 | 0.015 | 0.31 |
| Roseburia | rs6930661 | C | T | 0.076 | -0.096 | 0.020 | 2.48E-06 | 0.031 | 0.031 | 0.32 |
| Roseburia | rs75326254 | C | T | 0.064 | -0.105 | 0.023 | 7.50E-06 | -0.004 | 0.030 | 0.91 |
| Roseburia | rs78753150 | A | C | 0.059 | 0.097 | 0.021 | 9.98E-06 | 0.020 | 0.025 | 0.44 |
| Roseburia | rs9300744 | C | T | 0.239 | -0.059 | 0.013 | 4.75E-06 | 0.007 | 0.016 | 0.69 |
| Oscillibacter | rs11627628 | T | C | 0.073 | 0.144 | 0.029 | 1.01E-06 | 0.052 | 0.030 | 0.09 |
| Oscillibacter | rs11990279 | T | C | 0.240 | -0.082 | 0.018 | 4.94E-06 | 0.001 | 0.019 | 0.95 |
| Oscillibacter | rs12417956 | C | G | 0.245 | 0.079 | 0.017 | 6.03E-06 | 0.002 | 0.016 | 0.92 |
| Oscillibacter | rs12649930 | T | G | 0.094 | 0.122 | 0.026 | 4.09E-06 | -0.026 | 0.022 | 0.23 |
| Oscillibacter | rs133832 | A | C | 0.313 | -0.080 | 0.016 | 1.15E-06 | 0.012 | 0.016 | 0.46 |
| Oscillibacter | rs137917150 | T | A | 0.062 | -0.175 | 0.039 | 4.62E-06 | 0.004 | 0.034 | 0.90 |
| Oscillibacter | rs16866406 | A | G | 0.139 | 0.099 | 0.021 | 3.08E-06 | 0.014 | 0.018 | 0.45 |
| Oscillibacter | rs16934185 | A | G | 0.104 | -0.130 | 0.028 | 4.38E-06 | -0.038 | 0.026 | 0.15 |
| Oscillibacter | rs234108 | A | G | 0.480 | 0.075 | 0.015 | 9.16E-07 | -0.011 | 0.015 | 0.47 |
| Oscillibacter | rs36095275 | C | T | 0.362 | -0.075 | 0.016 | 1.40E-06 | -0.009 | 0.015 | 0.55 |
| Oscillibacter | rs4506202 | A | G | 0.516 | -0.071 | 0.015 | 3.21E-06 | -0.021 | 0.014 | 0.14 |
| Oscillibacter | rs61883564 | A | G | 0.124 | -0.101 | 0.022 | 3.39E-06 | -0.033 | 0.023 | 0.16 |
| Oscillibacter | rs62206502 | C | A | 0.459 | -0.068 | 0.015 | 6.60E-06 | -0.005 | 0.018 | 0.77 |
| Oscillibacter | rs6901560 | C | G | 0.199 | 0.086 | 0.019 | 6.21E-06 | 0.048 | 0.019 | 0.01 |
| Oscillibacter | rs75453768 | G | T | 0.087 | 0.122 | 0.027 | 5.35E-06 | -0.014 | 0.028 | 0.62 |
| Oscillibacter | rs761240 | T | G | 0.067 | -0.177 | 0.039 | 2.04E-06 | 0.045 | 0.031 | 0.15 |
| Oscillibacter | rs9393920 | A | G | 0.435 | -0.074 | 0.015 | 9.92E-07 | 0.028 | 0.014 | 0.05 |
| Turicibacter | rs11649454 | G | C | 0.139 | 0.095 | 0.020 | 3.27E-06 | -0.016 | 0.019 | 0.40 |
| Turicibacter | rs11666533 | C | T | 0.080 | -0.112 | 0.025 | 7.37E-06 | 0.002 | 0.026 | 0.93 |
| Turicibacter | rs12603364 | T | C | 0.107 | 0.111 | 0.023 | 8.67E-07 | -0.013 | 0.028 | 0.64 |
| Turicibacter | rs149744580 | A | G | 0.053 | 0.170 | 0.032 | 7.01E-08 | 0.009 | 0.034 | 0.79 |
| Turicibacter | rs2834977 | T | C | 0.173 | -0.096 | 0.021 | 3.96E-06 | -0.003 | 0.020 | 0.87 |
| Turicibacter | rs2952020 | G | A | 0.223 | -0.076 | 0.017 | 5.63E-06 | 0.024 | 0.016 | 0.14 |
| Turicibacter | rs3734633 | G | A | 0.097 | -0.121 | 0.027 | 5.32E-06 | -0.011 | 0.026 | 0.69 |
| Turicibacter | rs4247078 | C | G | 0.338 | 0.071 | 0.016 | 5.46E-06 | 0.024 | 0.015 | 0.11 |
| Turicibacter | rs4869133 | G | A | 0.089 | 0.131 | 0.027 | 2.55E-06 | 0.022 | 0.026 | 0.39 |
| Turicibacter | rs55756211 | T | C | 0.116 | -0.115 | 0.024 | 2.81E-06 | -0.005 | 0.022 | 0.83 |
| Turicibacter | rs61265175 | G | C | 0.233 | -0.086 | 0.019 | 4.14E-06 | 0.003 | 0.020 | 0.89 |
| Turicibacter | rs7199484 | G | A | 0.332 | -0.073 | 0.016 | 5.77E-06 | -0.016 | 0.015 | 0.29 |
| Lactobacillus | rs11674854 | C | T | 0.427 | -0.085 | 0.018 | 1.59E-06 | 0.001 | 0.017 | 0.94 |
| Lactobacillus | rs12693845 | C | T | 0.357 | -0.081 | 0.018 | 8.96E-06 | -0.004 | 0.015 | 0.80 |
| Lactobacillus | rs1530559 | G | A | 0.568 | 0.080 | 0.018 | 4.93E-06 | -0.019 | 0.016 | 0.23 |
| Lactobacillus | rs16861661 | G | A | 0.050 | -0.183 | 0.038 | 1.28E-06 | 0.027 | 0.025 | 0.29 |
| Lactobacillus | rs62314653 | C | A | 0.063 | 0.188 | 0.039 | 2.24E-06 | -0.030 | 0.030 | 0.32 |
| Lactobacillus | rs7399658 | G | A | 0.216 | -0.107 | 0.022 | 3.12E-06 | 0.011 | 0.018 | 0.57 |
| Lactobacillus | rs75127669 | C | A | 0.071 | 0.140 | 0.031 | 6.83E-06 | 0.023 | 0.030 | 0.44 |
| Lactobacillus | rs768253 | T | G | 0.391 | -0.079 | 0.017 | 4.25E-06 | 0.003 | 0.014 | 0.84 |
| Lactobacillus | rs77478751 | A | G | 0.054 | -0.220 | 0.048 | 7.33E-06 | -0.007 | 0.030 | 0.81 |
| Lactobacillus | rs921925 | A | C | 0.262 | 0.099 | 0.020 | 9.72E-07 | 0.022 | 0.018 | 0.22 |
| Gordonibacter | rs117347059 | G | C | 0.207 | -0.128 | 0.029 | 9.17E-06 | -0.007 | 0.020 | 0.73 |
| Gordonibacter | rs13412653 | A | C | 0.354 | 0.108 | 0.024 | 8.61E-06 | -0.017 | 0.015 | 0.23 |
| Gordonibacter | rs16955299 | G | A | 0.084 | -0.196 | 0.043 | 6.37E-06 | 0.004 | 0.022 | 0.86 |
| Gordonibacter | rs322296 | G | A | 0.119 | 0.179 | 0.038 | 4.02E-06 | 0.032 | 0.026 | 0.22 |
| Gordonibacter | rs35042269 | C | A | 0.189 | -0.180 | 0.040 | 8.11E-06 | 0.072 | 0.025 | 0.00 |
| Gordonibacter | rs3765837 | T | G | 0.079 | -0.191 | 0.043 | 7.17E-06 | 0.054 | 0.027 | 0.04 |
| Gordonibacter | rs4596722 | A | G | 0.476 | 0.103 | 0.023 | 9.06E-06 | 0.020 | 0.014 | 0.16 |
| Gordonibacter | rs61934597 | C | T | 0.076 | -0.172 | 0.039 | 8.37E-06 | -0.004 | 0.031 | 0.89 |
| Gordonibacter | rs71545975 | A | G | 0.173 | -0.154 | 0.034 | 7.04E-06 | 0.017 | 0.025 | 0.49 |
| Gordonibacter | rs72714787 | C | A | 0.104 | 0.181 | 0.038 | 1.43E-06 | 0.024 | 0.023 | 0.29 |
| Gordonibacter | rs72939513 | A | G | 0.054 | -0.214 | 0.049 | 7.98E-06 | 0.058 | 0.035 | 0.09 |
| Gordonibacter | rs7294633 | C | T | 0.351 | 0.129 | 0.025 | 3.44E-07 | -0.009 | 0.015 | 0.57 |
| Gordonibacter | rs76287110 | A | T | 0.071 | -0.243 | 0.047 | 1.67E-07 | -0.014 | 0.029 | 0.62 |
| Gordonibacter | rs768830 | G | A | 0.129 | 0.150 | 0.033 | 7.76E-06 | 0.024 | 0.021 | 0.26 |
| LachnospiraceaeNC2004group | rs117467633 | T | C | 0.063 | -0.170 | 0.038 | 9.13E-06 | 0.072 | 0.033 | 0.03 |
| LachnospiraceaeNC2004group | rs12127733 | G | A | 0.178 | 0.115 | 0.025 | 3.11E-06 | -0.020 | 0.019 | 0.30 |
| LachnospiraceaeNC2004group | rs12208226 | C | A | 0.106 | -0.155 | 0.034 | 9.75E-06 | 0.021 | 0.027 | 0.45 |
| LachnospiraceaeNC2004group | rs12863463 | G | A | 0.055 | -0.156 | 0.035 | 6.04E-06 | 0.001 | 0.030 | 0.97 |
| LachnospiraceaeNC2004group | rs1331592 | C | G | 0.229 | 0.095 | 0.021 | 5.34E-06 | 0.012 | 0.016 | 0.47 |
| LachnospiraceaeNC2004group | rs17067076 | G | A | 0.077 | -0.155 | 0.035 | 5.61E-06 | -0.011 | 0.022 | 0.63 |
| LachnospiraceaeNC2004group | rs1928659 | T | C | 0.250 | 0.103 | 0.023 | 6.17E-06 | 0.016 | 0.019 | 0.40 |
| LachnospiraceaeNC2004group | rs1929743 | T | C | 0.312 | 0.084 | 0.019 | 9.06E-06 | -0.007 | 0.015 | 0.65 |
| LachnospiraceaeNC2004group | rs3756315 | A | G | 0.339 | -0.088 | 0.019 | 3.33E-06 | -0.001 | 0.015 | 0.94 |
| LachnospiraceaeNC2004group | rs6116753 | G | A | 0.240 | 0.099 | 0.021 | 2.92E-06 | -0.011 | 0.017 | 0.49 |
| Prevotella7 | rs118038478 | A | G | 0.076 | 0.206 | 0.047 | 7.85E-06 | 0.037 | 0.030 | 0.22 |
| Prevotella7 | rs12124567 | A | G | 0.262 | -0.121 | 0.028 | 9.49E-06 | -0.020 | 0.017 | 0.25 |
| Prevotella7 | rs12195431 | T | C | 0.079 | 0.197 | 0.044 | 8.73E-06 | 0.035 | 0.026 | 0.19 |
| Prevotella7 | rs16937247 | G | C | 0.140 | 0.146 | 0.035 | 9.64E-06 | -0.018 | 0.019 | 0.33 |
| Prevotella7 | rs2240542 | C | T | 0.258 | 0.121 | 0.026 | 4.84E-06 | 0.014 | 0.015 | 0.36 |
| Prevotella7 | rs2918132 | C | T | 0.368 | -0.115 | 0.025 | 6.42E-06 | -0.012 | 0.016 | 0.46 |
| Prevotella7 | rs430270 | A | C | 0.201 | 0.139 | 0.030 | 2.87E-06 | -0.005 | 0.017 | 0.78 |
| Prevotella7 | rs57404562 | C | A | 0.180 | 0.155 | 0.032 | 6.22E-07 | 0.032 | 0.018 | 0.08 |
| Prevotella7 | rs79263163 | A | C | 0.185 | -0.144 | 0.032 | 7.51E-06 | -0.025 | 0.018 | 0.18 |
| Prevotella7 | rs9426434 | T | C | 0.273 | -0.124 | 0.028 | 9.72E-06 | 0.003 | 0.016 | 0.84 |
| Prevotella7 | rs9608249 | A | G | 0.171 | -0.158 | 0.034 | 2.07E-06 | 0.039 | 0.020 | 0.06 |
| Prevotella7 | rs9959718 | G | A | 0.250 | 0.133 | 0.028 | 1.90E-06 | 0.051 | 0.016 | 0.00 |
| Victivallis | rs11899949 | G | A | 0.277 | 0.131 | 0.028 | 2.77E-06 | -0.003 | 0.016 | 0.86 |
| Victivallis | rs12512543 | A | C | 0.121 | -0.178 | 0.037 | 2.54E-06 | 0.003 | 0.023 | 0.90 |
| Victivallis | rs173120 | T | C | 0.246 | 0.134 | 0.029 | 7.65E-06 | 0.017 | 0.018 | 0.34 |
| Victivallis | rs1882775 | A | G | 0.224 | -0.138 | 0.031 | 8.73E-06 | -0.008 | 0.018 | 0.64 |
| Victivallis | rs2546432 | T | C | 0.466 | -0.111 | 0.025 | 9.93E-06 | 0.008 | 0.015 | 0.61 |
| Victivallis | rs342302 | A | G | 0.159 | -0.153 | 0.035 | 8.16E-06 | -0.041 | 0.021 | 0.05 |
| Victivallis | rs4764863 | G | A | 0.500 | 0.122 | 0.025 | 8.22E-07 | 0.019 | 0.014 | 0.18 |
| Victivallis | rs4895919 | T | C | 0.482 | -0.117 | 0.025 | 2.75E-06 | -0.012 | 0.014 | 0.40 |
| Victivallis | rs56349194 | A | G | 0.160 | -0.159 | 0.032 | 6.26E-07 | -0.015 | 0.018 | 0.41 |
| Victivallis | rs592514 | T | A | 0.128 | -0.181 | 0.039 | 2.60E-06 | 0.009 | 0.024 | 0.70 |
| Victivallis | rs911666 | T | C | 0.305 | -0.119 | 0.026 | 7.65E-06 | -0.012 | 0.015 | 0.43 |
| Phascolarctobacterium | rs11929846 | T | C | 0.210 | -0.070 | 0.016 | 8.88E-06 | -0.001 | 0.018 | 0.97 |
| Phascolarctobacterium | rs12618201 | A | G | 0.347 | 0.064 | 0.014 | 3.38E-06 | 0.004 | 0.015 | 0.81 |
| Phascolarctobacterium | rs1264476 | T | G | 0.200 | 0.077 | 0.017 | 4.30E-06 | 0.003 | 0.017 | 0.85 |
| Phascolarctobacterium | rs28525131 | G | A | 0.094 | -0.119 | 0.027 | 8.23E-06 | 0.011 | 0.028 | 0.70 |
| Phascolarctobacterium | rs56069061 | G | A | 0.101 | -0.111 | 0.023 | 1.87E-06 | 0.017 | 0.024 | 0.47 |
| Phascolarctobacterium | rs56157888 | A | C | 0.170 | 0.095 | 0.019 | 1.09E-06 | -0.016 | 0.021 | 0.43 |
| Phascolarctobacterium | rs6427992 | G | C | 0.393 | -0.065 | 0.014 | 2.09E-06 | -0.003 | 0.015 | 0.84 |
| Phascolarctobacterium | rs74540770 | G | A | 0.065 | -0.121 | 0.026 | 3.60E-06 | 0.034 | 0.028 | 0.23 |
| Phascolarctobacterium | rs74847270 | A | G | 0.088 | -0.105 | 0.023 | 5.73E-06 | -0.002 | 0.030 | 0.95 |
| Phascolarctobacterium | rs75882962 | T | C | 0.167 | 0.097 | 0.019 | 3.19E-07 | 0.013 | 0.022 | 0.54 |
| Phascolarctobacterium | rs76124218 | C | G | 0.052 | -0.159 | 0.034 | 2.67E-06 | 0.012 | 0.032 | 0.70 |
| Phascolarctobacterium | rs7982713 | G | A | 0.229 | 0.073 | 0.016 | 9.72E-06 | 0.011 | 0.016 | 0.52 |
| Eubacteriumoxidoreducensgroup | rs12129908 | C | A | 0.447 | 0.089 | 0.020 | 5.80E-06 | -0.007 | 0.015 | 0.61 |
| Eubacteriumoxidoreducensgroup | rs12423772 | G | T | 0.150 | 0.141 | 0.030 | 2.63E-06 | -0.004 | 0.021 | 0.87 |
| Eubacteriumoxidoreducensgroup | rs1425962 | G | C | 0.343 | 0.091 | 0.020 | 7.32E-06 | 0.002 | 0.015 | 0.90 |
| Eubacteriumoxidoreducensgroup | rs2973294 | G | T | 0.421 | 0.092 | 0.020 | 2.39E-06 | 0.003 | 0.014 | 0.81 |
| Eubacteriumoxidoreducensgroup | rs34561138 | G | A | 0.059 | 0.216 | 0.046 | 2.51E-06 | 0.011 | 0.029 | 0.70 |
| Eubacteriumoxidoreducensgroup | rs440215 | C | T | 0.462 | 0.093 | 0.020 | 1.65E-06 | 0.016 | 0.015 | 0.26 |
| Oscillospira | rs12206468 | G | A | 0.064 | -0.133 | 0.027 | 1.04E-06 | 0.025 | 0.030 | 0.39 |
| Oscillospira | rs12925026 | T | C | 0.073 | 0.136 | 0.031 | 9.31E-06 | 0.006 | 0.027 | 0.82 |
| Oscillospira | rs1954532 | T | C | 0.209 | -0.083 | 0.018 | 2.27E-06 | -0.004 | 0.019 | 0.85 |
| Oscillospira | rs28889936 | A | C | 0.108 | 0.114 | 0.025 | 3.37E-06 | -0.021 | 0.028 | 0.46 |
| Oscillospira | rs62422654 | C | T | 0.147 | 0.090 | 0.020 | 6.47E-06 | -0.002 | 0.019 | 0.92 |
| Oscillospira | rs72866977 | A | C | 0.070 | -0.131 | 0.028 | 5.63E-06 | -0.007 | 0.030 | 0.82 |
| Oscillospira | rs73038677 | T | A | 0.182 | -0.083 | 0.017 | 1.09E-06 | -0.014 | 0.017 | 0.42 |
| Oscillospira | rs751183 | T | C | 0.246 | -0.077 | 0.017 | 6.85E-06 | -0.021 | 0.017 | 0.22 |
| Oscillospira | rs8076323 | A | G | 0.258 | 0.072 | 0.016 | 5.61E-06 | 0.027 | 0.016 | 0.10 |
| Catenibacterium | rs12404911 | C | T | 0.170 | 0.141 | 0.030 | 2.80E-06 | 0.004 | 0.017 | 0.84 |
| Catenibacterium | rs212393 | G | A | 0.310 | -0.135 | 0.029 | 3.62E-06 | -0.006 | 0.017 | 0.71 |
| Catenibacterium | rs73128290 | A | G | 0.263 | 0.130 | 0.028 | 4.29E-06 | 0.028 | 0.018 | 0.12 |
| Catenibacterium | rs77285108 | G | A | 0.146 | -0.162 | 0.035 | 3.63E-06 | 0.037 | 0.025 | 0.14 |
| Catenibacterium | rs7742829 | C | T | 0.420 | 0.114 | 0.025 | 5.61E-06 | 0.007 | 0.014 | 0.64 |
| Ruminococcus2 | rs12406309 | A | C | 0.191 | -0.063 | 0.014 | 9.79E-06 | -0.011 | 0.017 | 0.50 |
| Ruminococcus2 | rs12986628 | C | T | 0.213 | 0.067 | 0.014 | 2.14E-06 | -0.007 | 0.019 | 0.71 |
| Ruminococcus2 | rs1819812 | G | T | 0.116 | 0.084 | 0.018 | 5.28E-06 | -0.021 | 0.024 | 0.37 |
| Ruminococcus2 | rs2368224 | T | G | 0.048 | 0.200 | 0.044 | 3.63E-06 | 0.014 | 0.028 | 0.60 |
| Ruminococcus2 | rs2846589 | G | T | 0.454 | 0.052 | 0.012 | 7.59E-06 | -0.001 | 0.014 | 0.93 |
| Ruminococcus2 | rs2997412 | A | G | 0.280 | -0.057 | 0.012 | 4.22E-06 | -0.005 | 0.016 | 0.75 |
| Ruminococcus2 | rs4400279 | A | G | 0.364 | 0.055 | 0.012 | 5.80E-06 | 0.007 | 0.015 | 0.67 |
| Ruminococcus2 | rs4799823 | C | T | 0.139 | 0.084 | 0.018 | 5.40E-06 | 0.045 | 0.024 | 0.06 |
| Ruminococcus2 | rs55707116 | C | A | 0.102 | 0.087 | 0.019 | 8.01E-06 | 0.036 | 0.023 | 0.12 |
| Ruminococcus2 | rs58681734 | A | G | 0.151 | 0.072 | 0.016 | 4.18E-06 | -0.023 | 0.021 | 0.26 |
| Ruminococcus2 | rs61791565 | T | C | 0.369 | -0.052 | 0.012 | 6.79E-06 | -0.014 | 0.014 | 0.33 |
| Ruminococcus2 | rs75140805 | T | G | 0.158 | 0.084 | 0.018 | 3.95E-06 | -0.013 | 0.023 | 0.59 |
| Ruminococcus2 | rs7635831 | G | A | 0.293 | 0.062 | 0.013 | 1.98E-06 | 0.015 | 0.017 | 0.37 |
| Ruminococcus2 | rs7693984 | G | A | 0.083 | -0.103 | 0.024 | 9.42E-06 | 0.019 | 0.034 | 0.57 |
| Ruminococcus2 | rs78120384 | A | G | 0.073 | -0.193 | 0.039 | 3.31E-07 | 0.038 | 0.034 | 0.26 |
| Holdemanella | rs12415649 | G | C | 0.238 | 0.084 | 0.019 | 7.88E-06 | 0.012 | 0.018 | 0.50 |
| Holdemanella | rs12513188 | G | A | 0.222 | 0.090 | 0.020 | 4.65E-06 | -0.014 | 0.017 | 0.41 |
| Holdemanella | rs17586763 | T | C | 0.018 | -0.227 | 0.051 | 7.72E-06 | -0.084 | 0.038 | 0.03 |
| Holdemanella | rs1830029 | C | G | 0.180 | -0.095 | 0.021 | 5.35E-06 | -0.024 | 0.018 | 0.18 |
| Holdemanella | rs1926302 | G | A | 0.153 | -0.108 | 0.023 | 7.50E-06 | 0.013 | 0.019 | 0.48 |
| Holdemanella | rs34187114 | C | A | 0.166 | -0.105 | 0.023 | 5.13E-06 | 0.011 | 0.021 | 0.62 |
| Holdemanella | rs35228298 | G | A | 0.247 | 0.093 | 0.020 | 7.30E-06 | 0.025 | 0.019 | 0.19 |
| Holdemanella | rs4541991 | T | C | 0.236 | -0.093 | 0.019 | 2.10E-06 | 0.013 | 0.017 | 0.45 |
| Holdemanella | rs607782 | T | C | 0.382 | -0.085 | 0.017 | 7.19E-07 | -0.010 | 0.015 | 0.50 |
| Holdemanella | rs62113381 | T | C | 0.159 | -0.105 | 0.023 | 5.54E-06 | 0.060 | 0.022 | 0.01 |
| Holdemanella | rs73011279 | T | C | 0.208 | -0.096 | 0.020 | 1.36E-06 | 0.036 | 0.018 | 0.05 |
| Holdemanella | rs75764681 | T | C | 0.063 | -0.283 | 0.060 | 1.94E-06 | -0.035 | 0.039 | 0.37 |
| Holdemanella | rs761624 | C | G | 0.308 | 0.096 | 0.018 | 1.38E-07 | -0.006 | 0.016 | 0.69 |
| Holdemanella | rs8113760 | G | A | 0.352 | 0.079 | 0.017 | 4.62E-06 | -0.013 | 0.017 | 0.44 |
| RikenellaceaeRC9gutgroup | rs12501673 | A | G | 0.325 | 0.116 | 0.026 | 6.29E-06 | 0.026 | 0.016 | 0.11 |
| RikenellaceaeRC9gutgroup | rs17032291 | T | C | 0.141 | -0.170 | 0.037 | 6.61E-06 | 0.008 | 0.021 | 0.69 |
| RikenellaceaeRC9gutgroup | rs17582787 | A | G | 0.129 | -0.158 | 0.034 | 3.55E-06 | -0.015 | 0.020 | 0.46 |
| RikenellaceaeRC9gutgroup | rs2074881 | T | C | 0.179 | -0.142 | 0.032 | 9.45E-06 | -0.020 | 0.022 | 0.36 |
| RikenellaceaeRC9gutgroup | rs2900503 | G | T | 0.171 | -0.172 | 0.033 | 1.55E-07 | 0.006 | 0.019 | 0.74 |
| RikenellaceaeRC9gutgroup | rs2998141 | T | C | 0.222 | -0.136 | 0.029 | 4.42E-06 | 0.003 | 0.017 | 0.86 |
| RikenellaceaeRC9gutgroup | rs4270579 | G | A | 0.304 | -0.118 | 0.027 | 5.46E-06 | 0.015 | 0.017 | 0.37 |
| RikenellaceaeRC9gutgroup | rs4717843 | G | T | 0.362 | -0.119 | 0.026 | 4.72E-06 | -0.030 | 0.016 | 0.06 |
| RikenellaceaeRC9gutgroup | rs7193937 | G | C | 0.289 | 0.124 | 0.028 | 6.19E-06 | 0.001 | 0.016 | 0.97 |
| RikenellaceaeRC9gutgroup | rs7712231 | A | G | 0.165 | 0.156 | 0.035 | 7.97E-06 | 0.014 | 0.019 | 0.44 |
| RikenellaceaeRC9gutgroup | rs80309088 | G | A | 0.129 | 0.174 | 0.038 | 4.56E-06 | 0.028 | 0.024 | 0.24 |
| RikenellaceaeRC9gutgroup | rs9887954 | G | A | 0.436 | -0.115 | 0.025 | 4.81E-06 | 0.005 | 0.014 | 0.71 |
| RuminococcaceaeUCG010 | rs12597105 | G | A | 0.277 | 0.067 | 0.014 | 4.87E-06 | 0.015 | 0.016 | 0.35 |
| RuminococcaceaeUCG010 | rs2820282 | A | C | 0.452 | -0.059 | 0.013 | 2.85E-06 | -0.003 | 0.015 | 0.86 |
| RuminococcaceaeUCG010 | rs35506912 | G | C | 0.250 | -0.069 | 0.015 | 3.21E-06 | -0.030 | 0.018 | 0.09 |
| RuminococcaceaeUCG010 | rs682403 | A | G | 0.476 | -0.059 | 0.012 | 2.37E-06 | -0.008 | 0.014 | 0.58 |
| RuminococcaceaeUCG010 | rs6958419 | C | T | 0.489 | -0.059 | 0.012 | 2.84E-06 | -0.012 | 0.014 | 0.42 |
| RuminococcaceaeUCG010 | rs73218807 | G | A | 0.058 | -0.166 | 0.037 | 6.43E-06 | -0.036 | 0.034 | 0.29 |
| RuminococcaceaeUCG010 | rs7441445 | C | T | 0.435 | -0.057 | 0.013 | 6.80E-06 | 0.005 | 0.014 | 0.74 |
| RuminococcaceaeUCG010 | rs7935775 | A | T | 0.300 | -0.063 | 0.014 | 4.99E-06 | 0.009 | 0.016 | 0.57 |
| Alloprevotella | rs12675596 | G | T | 0.245 | 0.146 | 0.029 | 9.64E-07 | -0.011 | 0.018 | 0.51 |
| Alloprevotella | rs17380632 | A | T | 0.303 | 0.126 | 0.028 | 9.27E-06 | -0.016 | 0.016 | 0.32 |
| Alloprevotella | rs2154444 | T | G | 0.223 | 0.138 | 0.031 | 8.37E-06 | 0.010 | 0.017 | 0.55 |
| Alloprevotella | rs34619204 | G | A | 0.200 | -0.156 | 0.034 | 8.84E-06 | 0.017 | 0.019 | 0.38 |
| Alloprevotella | rs4364940 | A | G | 0.279 | 0.126 | 0.028 | 8.58E-06 | -0.012 | 0.015 | 0.43 |
| Alloprevotella | rs4680035 | A | G | 0.546 | -0.120 | 0.026 | 4.99E-06 | -0.001 | 0.015 | 0.93 |
| Alloprevotella | rs58212166 | A | G | 0.133 | -0.162 | 0.036 | 7.94E-06 | 0.036 | 0.019 | 0.05 |
| Eubacteriumeligensgroup | rs12719051 | A | G | 0.100 | 0.092 | 0.021 | 7.12E-06 | 0.060 | 0.029 | 0.04 |
| Eubacteriumeligensgroup | rs158115 | G | C | 0.092 | 0.092 | 0.019 | 9.74E-06 | 0.003 | 0.025 | 0.90 |
| Eubacteriumeligensgroup | rs182318 | G | A | 0.078 | -0.082 | 0.020 | 8.40E-06 | -0.013 | 0.023 | 0.58 |
| Eubacteriumeligensgroup | rs2200429 | A | G | 0.074 | -0.089 | 0.020 | 5.30E-06 | -0.024 | 0.022 | 0.29 |
| Eubacteriumeligensgroup | rs265534 | T | G | 0.455 | -0.056 | 0.012 | 2.27E-06 | -0.018 | 0.016 | 0.25 |
| Eubacteriumeligensgroup | rs4583233 | A | C | 0.334 | 0.067 | 0.013 | 2.84E-07 | 0.002 | 0.016 | 0.91 |
| Eubacteriumeligensgroup | rs56080211 | C | T | 0.056 | 0.123 | 0.028 | 9.14E-06 | 0.038 | 0.032 | 0.23 |
| Eubacteriumeligensgroup | rs6923695 | T | G | 0.057 | 0.103 | 0.023 | 4.87E-06 | 0.008 | 0.030 | 0.79 |
| Eubacteriumeligensgroup | rs72839198 | C | G | 0.056 | 0.156 | 0.038 | 3.14E-06 | 0.023 | 0.024 | 0.34 |
| Eubacteriumeligensgroup | rs74606150 | C | G | 0.046 | -0.196 | 0.043 | 4.25E-06 | -0.004 | 0.038 | 0.91 |
| LachnospiraceaeND3007group | rs13110238 | C | G | 0.245 | -0.064 | 0.014 | 4.48E-06 | 0.031 | 0.022 | 0.15 |
| LachnospiraceaeND3007group | rs2861203 | G | A | 0.319 | 0.057 | 0.013 | 7.37E-06 | 0.012 | 0.016 | 0.45 |
| LachnospiraceaeND3007group | rs72776675 | T | C | 0.175 | -0.065 | 0.015 | 8.72E-06 | 0.025 | 0.019 | 0.20 |
| LachnospiraceaeND3007group | rs9932954 | A | G | 0.428 | -0.056 | 0.012 | 1.25E-06 | -0.005 | 0.014 | 0.75 |
| Lachnospira | rs13157098 | A | G | 0.167 | -0.077 | 0.016 | 5.99E-07 | 0.034 | 0.022 | 0.13 |
| Lachnospira | rs159484 | G | A | 0.113 | 0.079 | 0.018 | 6.68E-06 | 0.010 | 0.024 | 0.67 |
| Lachnospira | rs2326833 | C | G | 0.125 | -0.078 | 0.017 | 4.60E-06 | 0.002 | 0.024 | 0.92 |
| Lachnospira | rs2520509 | A | G | 0.318 | 0.052 | 0.012 | 7.42E-06 | -0.001 | 0.015 | 0.96 |
| Lachnospira | rs4686798 | T | C | 0.386 | 0.053 | 0.011 | 2.74E-06 | -0.024 | 0.015 | 0.10 |
| Lachnospira | rs4923324 | G | A | 0.239 | -0.062 | 0.013 | 2.44E-06 | 0.001 | 0.018 | 0.96 |
| Lachnospira | rs56791201 | T | C | 0.457 | 0.052 | 0.011 | 2.93E-06 | -0.002 | 0.014 | 0.89 |
| Erysipelatoclostridium | rs1434153 | G | A | 0.321 | -0.068 | 0.015 | 6.85E-06 | 0.005 | 0.015 | 0.75 |
| Erysipelatoclostridium | rs16936671 | C | T | 0.136 | -0.097 | 0.022 | 6.04E-06 | -0.004 | 0.023 | 0.85 |
| Erysipelatoclostridium | rs17804233 | T | C | 0.482 | -0.066 | 0.014 | 4.59E-06 | -0.004 | 0.015 | 0.77 |
| Erysipelatoclostridium | rs2901723 | C | A | 0.503 | 0.064 | 0.014 | 8.79E-06 | -0.035 | 0.015 | 0.02 |
| Erysipelatoclostridium | rs340991 | A | G | 0.298 | -0.074 | 0.016 | 3.75E-06 | 0.004 | 0.015 | 0.79 |
| Erysipelatoclostridium | rs34528142 | C | G | 0.171 | -0.088 | 0.020 | 6.13E-06 | 0.022 | 0.020 | 0.27 |
| Erysipelatoclostridium | rs3804326 | A | G | 0.068 | 0.141 | 0.034 | 9.85E-06 | 0.012 | 0.023 | 0.61 |
| Erysipelatoclostridium | rs45480394 | T | G | 0.362 | -0.069 | 0.015 | 7.66E-06 | -0.008 | 0.016 | 0.60 |
| Erysipelatoclostridium | rs4697572 | A | G | 0.246 | -0.081 | 0.016 | 7.59E-07 | -0.018 | 0.016 | 0.26 |
| Erysipelatoclostridium | rs58236560 | G | T | 0.137 | -0.111 | 0.023 | 2.16E-06 | 0.004 | 0.025 | 0.88 |
| Erysipelatoclostridium | rs61806970 | C | T | 0.065 | 0.143 | 0.032 | 9.09E-06 | 0.000 | 0.033 | 0.99 |
| Erysipelatoclostridium | rs622418 | A | G | 0.511 | -0.067 | 0.014 | 3.68E-06 | -0.002 | 0.015 | 0.88 |
| Erysipelatoclostridium | rs6474512 | A | C | 0.451 | 0.067 | 0.014 | 3.02E-06 | 0.010 | 0.015 | 0.48 |
| Erysipelatoclostridium | rs710230 | T | C | 0.087 | 0.143 | 0.028 | 6.33E-07 | 0.054 | 0.031 | 0.08 |
| Erysipelatoclostridium | rs7221249 | A | G | 0.547 | 0.084 | 0.014 | 4.31E-09 | 0.015 | 0.014 | 0.30 |
| Erysipelatoclostridium | rs9590927 | G | A | 0.494 | -0.065 | 0.014 | 6.39E-06 | 0.020 | 0.016 | 0.21 |
| Terrisporobacter | rs1883097 | C | T | 0.056 | 0.226 | 0.045 | 4.16E-07 | -0.037 | 0.029 | 0.20 |
| Terrisporobacter | rs2569953 | A | C | 0.420 | -0.078 | 0.017 | 8.95E-06 | 0.035 | 0.014 | 0.01 |
| Terrisporobacter | rs2872237 | C | A | 0.434 | -0.081 | 0.018 | 3.97E-06 | -0.005 | 0.014 | 0.72 |
| Terrisporobacter | rs58405430 | G | T | 0.115 | 0.135 | 0.030 | 7.94E-06 | 0.014 | 0.027 | 0.60 |
| Terrisporobacter | rs7184125 | T | C | 0.233 | 0.091 | 0.021 | 8.48E-06 | 0.010 | 0.017 | 0.58 |
| Allisonella | rs1901739 | T | G | 0.495 | 0.116 | 0.025 | 3.59E-06 | 0.014 | 0.014 | 0.32 |
| Allisonella | rs35110698 | T | C | 0.149 | -0.146 | 0.032 | 5.72E-06 | -0.007 | 0.018 | 0.73 |
| Allisonella | rs35778461 | C | T | 0.192 | 0.147 | 0.030 | 1.21E-06 | 0.007 | 0.017 | 0.66 |
| Allisonella | rs594561 | C | T | 0.441 | 0.112 | 0.025 | 9.41E-06 | 0.002 | 0.014 | 0.88 |
| Allisonella | rs602075 | A | G | 0.231 | 0.169 | 0.030 | 3.57E-08 | -0.002 | 0.017 | 0.91 |
| Allisonella | rs6742198 | G | A | 0.196 | 0.149 | 0.032 | 3.35E-06 | -0.017 | 0.018 | 0.33 |
| Allisonella | rs685403 | G | C | 0.108 | -0.175 | 0.040 | 4.88E-06 | 0.010 | 0.027 | 0.71 |
| Allisonella | rs76904847 | G | A | 0.190 | 0.149 | 0.033 | 6.09E-06 | -0.002 | 0.020 | 0.92 |
| Allisonella | rs7898615 | T | G | 0.115 | 0.168 | 0.037 | 8.87E-06 | -0.025 | 0.020 | 0.21 |

MR, Mendelian randomization; CRF, chronic renal failure; SNP, single nucleotide polymorphism; EAF, Effect Allele Frequency; SE, standard error.

**Supplementary Table 2** MR analysis results of gut microbiota from 119 genera related to CRF.

| **Exposure(Bacterial taxa)** | **MR method** | **No. of snp** | **Beta** | **OR** | **95% CI** | **P-value** |
| --- | --- | --- | --- | --- | --- | --- |
| Ruminiclostridium9 | MR Egger | 14 | -0.17 | 0.84 | 0.39-1.83 | 0.67 |
| Ruminiclostridium9 | Weighted median | 14 | 0.13 | 1.14 | 0.92-1.4 | 0.22 |
| Ruminiclostridium9 | **Inverse variance weighted** | 14 | 0.09 | 1.10 | 0.94-1.29 | 0.25 |
| Ruminiclostridium9 | Simple mode | 14 | 0.14 | 1.15 | 0.84-1.57 | 0.40 |
| Ruminiclostridium9 | Weighted mode | 14 | 0.15 | 1.16 | 0.86-1.57 | 0.35 |
| Lactobacillus | MR Egger | 10 | -0.07 | 0.94 | 0.69-1.26 | 0.68 |
| Lactobacillus | Weighted median | 10 | -0.03 | 0.97 | 0.85-1.11 | 0.65 |
| Lactobacillus | **Inverse variance weighted** | 10 | -0.03 | 0.97 | 0.87-1.08 | 0.56 |
| Lactobacillus | Simple mode | 10 | -0.05 | 0.95 | 0.76-1.2 | 0.70 |
| Lactobacillus | Weighted mode | 10 | -0.06 | 0.94 | 0.76-1.17 | 0.61 |
| Streptococcus | MR Egger | 17 | -0.29 | 0.75 | 0.41-1.37 | 0.36 |
| Streptococcus | Weighted median | 17 | -0.02 | 0.98 | 0.82-1.19 | 0.87 |
| Streptococcus | **Inverse variance weighted** | 17 | 0.05 | 1.05 | 0.9-1.22 | 0.56 |
| Streptococcus | Simple mode | 17 | -0.05 | 0.95 | 0.7-1.3 | 0.76 |
| Streptococcus | Weighted mode | 17 | -0.08 | 0.93 | 0.68-1.27 | 0.65 |
| Ruminiclostridium5 | MR Egger | 14 | -0.19 | 0.82 | 0.58-1.17 | 0.30 |
| Ruminiclostridium5 | Weighted median | 14 | 0.03 | 1.03 | 0.86-1.23 | 0.76 |
| Ruminiclostridium5 | **Inverse variance weighted** | 14 | 0.03 | 1.03 | 0.9-1.18 | 0.66 |
| Ruminiclostridium5 | Simple mode | 14 | 0.07 | 1.08 | 0.78-1.49 | 0.66 |
| Ruminiclostridium5 | Weighted mode | 14 | -0.05 | 0.95 | 0.73-1.22 | 0.68 |
| Butyricicoccus | MR Egger | 9 | 0.05 | 1.05 | 0.79-1.39 | 0.76 |
| Butyricicoccus | Weighted median | 9 | -0.02 | 0.98 | 0.8-1.19 | 0.84 |
| Butyricicoccus | **Inverse variance weighted** | 9 | -0.12 | 0.89 | 0.76-1.04 | 0.15 |
| Butyricicoccus | Simple mode | 9 | -0.10 | 0.91 | 0.69-1.18 | 0.49 |
| Butyricicoccus | Weighted mode | 9 | -0.02 | 0.98 | 0.79-1.22 | 0.86 |
| Victivallis | MR Egger | 11 | 0.00 | 1.00 | 0.62-1.63 | 0.99 |
| Victivallis | Weighted median | 11 | 0.09 | 1.09 | 0.99-1.21 | 0.07 |
| Victivallis | **Inverse variance weighted** | 11 | 0.07 | 1.07 | 0.99-1.15 | 0.07 |
| Victivallis | Simple mode | 11 | 0.10 | 1.10 | 0.94-1.29 | 0.25 |
| Victivallis | Weighted mode | 11 | 0.10 | 1.10 | 0.95-1.28 | 0.23 |
| ErysipelotrichaceaeUCG003 | MR Egger | 17 | -0.16 | 0.85 | 0.55-1.33 | 0.49 |
| ErysipelotrichaceaeUCG003 | Weighted median | 17 | -0.08 | 0.92 | 0.77-1.1 | 0.37 |
| ErysipelotrichaceaeUCG003 | **Inverse variance weighted** | 17 | -0.05 | 0.95 | 0.82-1.12 | 0.57 |
| ErysipelotrichaceaeUCG003 | Simple mode | 17 | 0.02 | 1.02 | 0.72-1.45 | 0.92 |
| ErysipelotrichaceaeUCG003 | Weighted mode | 17 | -0.03 | 0.97 | 0.72-1.31 | 0.83 |
| Haemophilus | MR Egger | 13 | -0.32 | 0.73 | 0.54-0.98 | 0.06 |
| Haemophilus | Weighted median | 13 | 0.01 | 1.01 | 0.87-1.17 | 0.91 |
| Haemophilus | **Inverse variance weighted** | 13 | 0.02 | 1.02 | 0.89-1.16 | 0.83 |
| Haemophilus | Simple mode | 13 | -0.06 | 0.94 | 0.72-1.23 | 0.64 |
| Haemophilus | Weighted mode | 13 | -0.05 | 0.95 | 0.76-1.19 | 0.65 |
| Blautia | MR Egger | 13 | 0.07 | 1.08 | 0.71-1.62 | 0.73 |
| Blautia | Weighted median | 13 | 0.00 | 1.00 | 0.81-1.23 | 0.96 |
| Blautia | **Inverse variance weighted** | 13 | 0.04 | 1.04 | 0.89-1.22 | 0.62 |
| Blautia | Simple mode | 13 | -0.02 | 0.98 | 0.71-1.35 | 0.91 |
| Blautia | Weighted mode | 13 | -0.02 | 0.98 | 0.71-1.36 | 0.92 |
| Oxalobacter | MR Egger | 12 | 0.08 | 1.09 | 0.71-1.66 | 0.71 |
| Oxalobacter | Weighted median | 12 | 0.03 | 1.03 | 0.92-1.15 | 0.62 |
| Oxalobacter | **Inverse variance weighted** | 12 | 0.04 | 1.05 | 0.96-1.14 | 0.34 |
| Oxalobacter | Simple mode | 12 | 0.04 | 1.04 | 0.88-1.22 | 0.68 |
| Oxalobacter | Weighted mode | 12 | 0.03 | 1.03 | 0.89-1.2 | 0.68 |
| Anaerofilum | MR Egger | 12 | 0.02 | 1.02 | 0.68-1.54 | 0.92 |
| Anaerofilum | Weighted median | 12 | -0.06 | 0.94 | 0.84-1.04 | 0.24 |
| Anaerofilum | **Inverse variance weighted** | 12 | -0.04 | 0.96 | 0.88-1.05 | 0.36 |
| Anaerofilum | Simple mode | 12 | -0.08 | 0.92 | 0.77-1.11 | 0.41 |
| Anaerofilum | Weighted mode | 12 | -0.08 | 0.93 | 0.78-1.1 | 0.40 |
| LachnospiraceaeNK4A136group | MR Egger | 16 | 0.11 | 1.12 | 0.86-1.47 | 0.42 |
| LachnospiraceaeNK4A136group | Weighted median | 16 | 0.03 | 1.03 | 0.86-1.22 | 0.75 |
| LachnospiraceaeNK4A136group | **Inverse variance weighted** | 16 | -0.05 | 0.95 | 0.84-1.09 | 0.48 |
| LachnospiraceaeNK4A136group | Simple mode | 16 | 0.01 | 1.01 | 0.72-1.41 | 0.95 |
| LachnospiraceaeNK4A136group | Weighted mode | 16 | 0.07 | 1.07 | 0.86-1.33 | 0.55 |
| Eisenbergiella | MR Egger | 12 | -0.49 | 0.61 | 0.31-1.2 | 0.18 |
| Eisenbergiella | Weighted median | 12 | 0.07 | 1.08 | 0.95-1.22 | 0.25 |
| Eisenbergiella | **Inverse variance weighted** | 12 | 0.08 | 1.08 | 0.99-1.19 | 0.08 |
| Eisenbergiella | Simple mode | 12 | 0.18 | 1.20 | 0.99-1.46 | 0.09 |
| Eisenbergiella | Weighted mode | 12 | 0.15 | 1.16 | 0.95-1.42 | 0.18 |
| Prevotella7 | MR Egger | 12 | 0.08 | 1.08 | 0.56-2.09 | 0.82 |
| Prevotella7 | Weighted median | 12 | 0.13 | 1.13 | 1.02-1.26 | 0.02 |
| Prevotella7 | **Inverse variance weighted** | 12 | 0.09 | 1.09 | 0.99-1.21 | 0.07 |
| Prevotella7 | Simple mode | 12 | 0.17 | 1.18 | 0.98-1.43 | 0.12 |
| Prevotella7 | Weighted mode | 12 | 0.17 | 1.18 | 0.97-1.44 | 0.13 |
| Eggerthella | MR Egger | 11 | 0.23 | 1.26 | 0.78-2.02 | 0.37 |
| Eggerthella | Weighted median | 11 | -0.02 | 0.98 | 0.86-1.11 | 0.72 |
| Eggerthella | **Inverse variance weighted** | 11 | 0.01 | 1.01 | 0.92-1.12 | 0.77 |
| Eggerthella | Simple mode | 11 | -0.03 | 0.97 | 0.79-1.19 | 0.78 |
| Eggerthella | Weighted mode | 11 | -0.04 | 0.96 | 0.79-1.17 | 0.68 |
| FamilyXIIIUCG001 | MR Egger | 10 | -0.09 | 0.91 | 0.47-1.76 | 0.79 |
| FamilyXIIIUCG001 | Weighted median | 10 | 0.13 | 1.14 | 0.92-1.43 | 0.23 |
| FamilyXIIIUCG001 | **Inverse variance weighted** | 10 | 0.13 | 1.14 | 0.92-1.4 | 0.23 |
| FamilyXIIIUCG001 | Simple mode | 10 | 0.35 | 1.41 | 0.98-2.04 | 0.10 |
| FamilyXIIIUCG001 | Weighted mode | 10 | 0.34 | 1.40 | 1-1.96 | 0.08 |
| RikenellaceaeRC9gutgroup | MR Egger | 12 | 0.00 | 1.00 | 0.63-1.6 | 0.99 |
| RikenellaceaeRC9gutgroup | Weighted median | 12 | 0.00 | 1.00 | 0.91-1.11 | 0.96 |
| RikenellaceaeRC9gutgroup | **Inverse variance weighted** | 12 | 0.05 | 1.05 | 0.98-1.13 | 0.19 |
| RikenellaceaeRC9gutgroup | Simple mode | 12 | -0.02 | 0.98 | 0.82-1.16 | 0.79 |
| RikenellaceaeRC9gutgroup | Weighted mode | 12 | -0.02 | 0.98 | 0.84-1.14 | 0.76 |
| Paraprevotella | MR Egger | 13 | 0.09 | 1.09 | 0.78-1.52 | 0.61 |
| Paraprevotella | Weighted median | 13 | -0.02 | 0.98 | 0.87-1.1 | 0.72 |
| Paraprevotella | **Inverse variance weighted** | 13 | 0.01 | 1.01 | 0.92-1.11 | 0.82 |
| Paraprevotella | Simple mode | 13 | -0.03 | 0.97 | 0.8-1.19 | 0.79 |
| Paraprevotella | Weighted mode | 13 | -0.03 | 0.97 | 0.81-1.18 | 0.79 |
| Parabacteroides | MR Egger | 9 | -0.39 | 0.68 | 0.41-1.13 | 0.18 |
| Parabacteroides | Weighted median | 9 | -0.04 | 0.96 | 0.76-1.23 | 0.76 |
| Parabacteroides | **Inverse variance weighted** | 9 | -0.16 | 0.85 | 0.71-1.03 | 0.10 |
| Parabacteroides | Simple mode | 9 | -0.03 | 0.97 | 0.65-1.45 | 0.87 |
| Parabacteroides | Weighted mode | 9 | 0.01 | 1.01 | 0.72-1.4 | 0.96 |
| Ruminococcustorquesgroup | MR Egger | 13 | 0.14 | 1.15 | 0.66-1.99 | 0.64 |
| Ruminococcustorquesgroup | Weighted median | 13 | 0.05 | 1.05 | 0.84-1.32 | 0.64 |
| Ruminococcustorquesgroup | **Inverse variance weighted** | 13 | 0.07 | 1.07 | 0.88-1.3 | 0.51 |
| Ruminococcustorquesgroup | Simple mode | 13 | 0.11 | 1.11 | 0.78-1.6 | 0.57 |
| Ruminococcustorquesgroup | Weighted mode | 13 | 0.05 | 1.05 | 0.8-1.38 | 0.75 |
| Alistipes | MR Egger | 15 | -0.11 | 0.90 | 0.35-2.33 | 0.83 |
| Alistipes | Weighted median | 15 | -0.19 | 0.82 | 0.66-1.03 | 0.09 |
| Alistipes | **Inverse variance weighted** | 15 | -0.07 | 0.93 | 0.78-1.12 | 0.46 |
| Alistipes | Simple mode | 15 | -0.24 | 0.78 | 0.54-1.14 | 0.22 |
| Alistipes | Weighted mode | 15 | -0.24 | 0.78 | 0.54-1.14 | 0.23 |
| Escherichia.Shigella | MR Egger | 15 | -0.06 | 0.94 | 0.65-1.35 | 0.74 |
| Escherichia.Shigella | Weighted median | 15 | 0.15 | 1.16 | 0.98-1.37 | 0.09 |
| Escherichia.Shigella | **Inverse variance weighted** | 15 | 0.20 | 1.22 | 1.08-1.38 | 0.00 |
| Escherichia.Shigella | Simple mode | 15 | 0.34 | 1.41 | 1.03-1.92 | 0.05 |
| Escherichia.Shigella | Weighted mode | 15 | 0.33 | 1.39 | 1.01-1.9 | 0.06 |
| Desulfovibrio | MR Egger | 10 | 0.12 | 1.13 | 0.76-1.68 | 0.57 |
| Desulfovibrio | Weighted median | 10 | 0.06 | 1.06 | 0.9-1.26 | 0.47 |
| Desulfovibrio | **Inverse variance weighted** | 10 | 0.03 | 1.03 | 0.91-1.17 | 0.62 |
| Desulfovibrio | Simple mode | 10 | 0.10 | 1.10 | 0.84-1.44 | 0.51 |
| Desulfovibrio | Weighted mode | 10 | 0.10 | 1.10 | 0.83-1.46 | 0.52 |
| Adlercreutzia | MR Egger | 12 | -0.10 | 0.90 | 0.52-1.56 | 0.72 |
| Adlercreutzia | Weighted median | 12 | 0.06 | 1.06 | 0.91-1.24 | 0.44 |
| Adlercreutzia | **Inverse variance weighted** | 12 | 0.05 | 1.05 | 0.94-1.18 | 0.39 |
| Adlercreutzia | Simple mode | 12 | 0.07 | 1.07 | 0.84-1.37 | 0.59 |
| Adlercreutzia | Weighted mode | 12 | 0.07 | 1.07 | 0.85-1.36 | 0.57 |
| LachnospiraceaeFCS020group | MR Egger | 15 | -0.19 | 0.82 | 0.63-1.08 | 0.19 |
| LachnospiraceaeFCS020group | Weighted median | 15 | -0.09 | 0.91 | 0.78-1.07 | 0.27 |
| LachnospiraceaeFCS020group | **Inverse variance weighted** | 15 | -0.02 | 0.98 | 0.87-1.1 | 0.69 |
| LachnospiraceaeFCS020group | Simple mode | 15 | -0.11 | 0.89 | 0.68-1.17 | 0.43 |
| LachnospiraceaeFCS020group | Weighted mode | 15 | -0.12 | 0.89 | 0.71-1.11 | 0.31 |
| Flavonifractor | MR Egger | 8 | 0.33 | 1.39 | 0.65-2.97 | 0.43 |
| Flavonifractor | Weighted median | 8 | -0.03 | 0.97 | 0.78-1.2 | 0.78 |
| Flavonifractor | **Inverse variance weighted** | 8 | 0.00 | 1.00 | 0.82-1.2 | 0.97 |
| Flavonifractor | Simple mode | 8 | -0.15 | 0.86 | 0.61-1.22 | 0.43 |
| Flavonifractor | Weighted mode | 8 | -0.11 | 0.90 | 0.61-1.31 | 0.59 |
| Sellimonas | MR Egger | 11 | 0.37 | 1.45 | 0.89-2.36 | 0.17 |
| Sellimonas | Weighted median | 11 | 0.02 | 1.02 | 0.92-1.12 | 0.71 |
| Sellimonas | **Inverse variance weighted** | 11 | 0.04 | 1.04 | 0.96-1.12 | 0.37 |
| Sellimonas | Simple mode | 11 | 0.00 | 1.00 | 0.85-1.18 | 0.98 |
| Sellimonas | Weighted mode | 11 | 0.00 | 1.00 | 0.85-1.16 | 0.96 |
| Romboutsia | MR Egger | 14 | -0.31 | 0.73 | 0.49-1.09 | 0.15 |
| Romboutsia | Weighted median | 14 | -0.09 | 0.91 | 0.76-1.09 | 0.31 |
| Romboutsia | **Inverse variance weighted** | 14 | -0.12 | 0.89 | 0.77-1.03 | 0.12 |
| Romboutsia | Simple mode | 14 | -0.04 | 0.96 | 0.69-1.32 | 0.80 |
| Romboutsia | Weighted mode | 14 | -0.05 | 0.95 | 0.68-1.34 | 0.79 |
| Marvinbryantia | MR Egger | 10 | 0.16 | 1.17 | 0.64-2.14 | 0.63 |
| Marvinbryantia | Weighted median | 10 | -0.10 | 0.90 | 0.74-1.09 | 0.29 |
| Marvinbryantia | **Inverse variance weighted** | 10 | -0.05 | 0.95 | 0.82-1.11 | 0.52 |
| Marvinbryantia | Simple mode | 10 | -0.14 | 0.87 | 0.65-1.17 | 0.39 |
| Marvinbryantia | Weighted mode | 10 | -0.13 | 0.88 | 0.66-1.16 | 0.37 |
| Collinsella | MR Egger | 11 | -0.51 | 0.60 | 0.29-1.25 | 0.20 |
| Collinsella | Weighted median | 11 | 0.07 | 1.07 | 0.85-1.36 | 0.57 |
| Collinsella | **Inverse variance weighted** | 11 | 0.02 | 1.02 | 0.84-1.23 | 0.87 |
| Collinsella | Simple mode | 11 | 0.07 | 1.08 | 0.72-1.6 | 0.73 |
| Collinsella | Weighted mode | 11 | 0.09 | 1.09 | 0.74-1.61 | 0.68 |
| Faecalibacterium | MR Egger | 13 | 0.17 | 1.18 | 0.89-1.57 | 0.28 |
| Faecalibacterium | Weighted median | 13 | 0.07 | 1.07 | 0.89-1.29 | 0.45 |
| Faecalibacterium | **Inverse variance weighted** | 13 | 0.04 | 1.04 | 0.9-1.19 | 0.62 |
| Faecalibacterium | Simple mode | 13 | 0.09 | 1.09 | 0.82-1.45 | 0.57 |
| Faecalibacterium | Weighted mode | 13 | 0.06 | 1.06 | 0.85-1.32 | 0.62 |
| Oscillospira | MR Egger | 9 | -0.48 | 0.62 | 0.33-1.16 | 0.18 |
| Oscillospira | Weighted median | 9 | 0.04 | 1.05 | 0.87-1.26 | 0.64 |
| Oscillospira | **Inverse variance weighted** | 9 | 0.07 | 1.07 | 0.93-1.23 | 0.37 |
| Oscillospira | Simple mode | 9 | 0.04 | 1.04 | 0.77-1.41 | 0.79 |
| Oscillospira | Weighted mode | 9 | 0.05 | 1.05 | 0.78-1.4 | 0.77 |
| Roseburia | MR Egger | 15 | -0.15 | 0.86 | 0.58-1.27 | 0.45 |
| Roseburia | Weighted median | 15 | -0.08 | 0.92 | 0.76-1.12 | 0.41 |
| Roseburia | **Inverse variance weighted** | 15 | -0.11 | 0.89 | 0.78-1.02 | 0.11 |
| Roseburia | Simple mode | 15 | 0.03 | 1.03 | 0.71-1.49 | 0.89 |
| Roseburia | Weighted mode | 15 | 0.02 | 1.02 | 0.71-1.46 | 0.93 |
| Intestinibacter | MR Egger | 15 | -0.05 | 0.95 | 0.63-1.43 | 0.82 |
| Intestinibacter | Weighted median | 15 | 0.02 | 1.02 | 0.86-1.21 | 0.81 |
| Intestinibacter | **Inverse variance weighted** | 15 | 0.04 | 1.04 | 0.92-1.17 | 0.51 |
| Intestinibacter | Simple mode | 15 | -0.07 | 0.93 | 0.69-1.26 | 0.66 |
| Intestinibacter | Weighted mode | 15 | -0.04 | 0.96 | 0.71-1.28 | 0.77 |
| Butyrivibrio | MR Egger | 16 | -0.18 | 0.84 | 0.64-1.1 | 0.22 |
| Butyrivibrio | Weighted median | 16 | 0.03 | 1.03 | 0.95-1.13 | 0.44 |
| Butyrivibrio | **Inverse variance weighted** | 16 | 0.04 | 1.04 | 0.98-1.11 | 0.19 |
| Butyrivibrio | Simple mode | 16 | 0.01 | 1.01 | 0.87-1.18 | 0.89 |
| Butyrivibrio | Weighted mode | 16 | 0.02 | 1.02 | 0.88-1.18 | 0.78 |
| Turicibacter | MR Egger | 12 | 0.00 | 1.00 | 0.61-1.64 | 1.00 |
| Turicibacter | Weighted median | 12 | 0.04 | 1.04 | 0.89-1.22 | 0.62 |
| Turicibacter | **Inverse variance weighted** | 12 | 0.03 | 1.03 | 0.91-1.16 | 0.64 |
| Turicibacter | Simple mode | 12 | 0.03 | 1.03 | 0.79-1.35 | 0.83 |
| Turicibacter | Weighted mode | 12 | 0.04 | 1.04 | 0.8-1.36 | 0.78 |
| DefluviitaleaceaeUCG011 | MR Egger | 10 | -0.51 | 0.60 | 0.34-1.06 | 0.12 |
| DefluviitaleaceaeUCG011 | Weighted median | 10 | -0.05 | 0.95 | 0.8-1.13 | 0.54 |
| DefluviitaleaceaeUCG011 | **Inverse variance weighted** | 10 | -0.08 | 0.93 | 0.78-1.09 | 0.37 |
| DefluviitaleaceaeUCG011 | Simple mode | 10 | 0.10 | 1.10 | 0.78-1.56 | 0.60 |
| DefluviitaleaceaeUCG011 | Weighted mode | 10 | 0.06 | 1.07 | 0.78-1.46 | 0.70 |
| LachnospiraceaeUCG001 | MR Egger | 16 | 0.35 | 1.41 | 0.86-2.34 | 0.20 |
| LachnospiraceaeUCG001 | Weighted median | 16 | -0.05 | 0.96 | 0.83-1.1 | 0.53 |
| LachnospiraceaeUCG001 | **Inverse variance weighted** | 16 | -0.06 | 0.94 | 0.85-1.05 | 0.27 |
| LachnospiraceaeUCG001 | Simple mode | 16 | -0.05 | 0.95 | 0.76-1.19 | 0.65 |
| LachnospiraceaeUCG001 | Weighted mode | 16 | -0.05 | 0.95 | 0.76-1.19 | 0.68 |
| Eubacteriumxylanophilumgroup | MR Egger | 11 | 0.26 | 1.30 | 0.76-2.22 | 0.36 |
| Eubacteriumxylanophilumgroup | Weighted median | 11 | -0.05 | 0.96 | 0.77-1.18 | 0.68 |
| Eubacteriumxylanophilumgroup | **Inverse variance weighted** | 11 | 0.03 | 1.03 | 0.87-1.2 | 0.76 |
| Eubacteriumxylanophilumgroup | Simple mode | 11 | -0.11 | 0.90 | 0.64-1.25 | 0.53 |
| Eubacteriumxylanophilumgroup | Weighted mode | 11 | -0.12 | 0.89 | 0.65-1.21 | 0.46 |
| Akkermansia | MR Egger | 12 | -0.19 | 0.83 | 0.51-1.36 | 0.47 |
| Akkermansia | Weighted median | 12 | -0.14 | 0.87 | 0.73-1.02 | 0.09 |
| Akkermansia | **Inverse variance weighted** | 12 | -0.12 | 0.89 | 0.78-1.01 | 0.08 |
| Akkermansia | Simple mode | 12 | -0.13 | 0.88 | 0.66-1.16 | 0.38 |
| Akkermansia | Weighted mode | 12 | -0.14 | 0.87 | 0.67-1.14 | 0.34 |
| LachnospiraceaeNC2004group | MR Egger | 10 | -0.25 | 0.78 | 0.5-1.22 | 0.31 |
| LachnospiraceaeNC2004group | Weighted median | 10 | -0.02 | 0.98 | 0.84-1.14 | 0.82 |
| LachnospiraceaeNC2004group | **Inverse variance weighted** | 10 | -0.05 | 0.95 | 0.86-1.06 | 0.38 |
| LachnospiraceaeNC2004group | Simple mode | 10 | -0.06 | 0.94 | 0.75-1.18 | 0.61 |
| LachnospiraceaeNC2004group | Weighted mode | 10 | -0.04 | 0.96 | 0.78-1.18 | 0.73 |
| RuminococcaceaeUCG005 | MR Egger | 17 | -0.37 | 0.69 | 0.46-1.05 | 0.10 |
| RuminococcaceaeUCG005 | Weighted median | 17 | -0.01 | 0.99 | 0.83-1.18 | 0.90 |
| RuminococcaceaeUCG005 | **Inverse variance weighted** | 17 | 0.10 | 1.10 | 0.94-1.29 | 0.24 |
| RuminococcaceaeUCG005 | Simple mode | 17 | -0.07 | 0.93 | 0.67-1.3 | 0.68 |
| RuminococcaceaeUCG005 | Weighted mode | 17 | -0.08 | 0.92 | 0.72-1.19 | 0.55 |
| Bilophila | MR Egger | 16 | 0.27 | 1.31 | 0.75-2.3 | 0.36 |
| Bilophila | Weighted median | 16 | 0.05 | 1.05 | 0.89-1.24 | 0.56 |
| Bilophila | **Inverse variance weighted** | 16 | 0.07 | 1.07 | 0.94-1.22 | 0.29 |
| Bilophila | Simple mode | 16 | 0.01 | 1.01 | 0.72-1.4 | 0.97 |
| Bilophila | Weighted mode | 16 | 0.00 | 1.00 | 0.71-1.41 | 0.99 |
| Slackia | MR Egger | 9 | -0.17 | 0.84 | 0.51-1.38 | 0.51 |
| Slackia | Weighted median | 9 | -0.07 | 0.94 | 0.81-1.08 | 0.38 |
| Slackia | **Inverse variance weighted** | 9 | -0.07 | 0.93 | 0.84-1.04 | 0.24 |
| Slackia | Simple mode | 9 | -0.07 | 0.93 | 0.74-1.17 | 0.55 |
| Slackia | Weighted mode | 9 | -0.07 | 0.93 | 0.76-1.14 | 0.51 |
| Eubacteriumbrachygroup | MR Egger | 11 | 0.06 | 1.06 | 0.74-1.52 | 0.76 |
| Eubacteriumbrachygroup | Weighted median | 11 | 0.04 | 1.04 | 0.92-1.18 | 0.55 |
| Eubacteriumbrachygroup | **Inverse variance weighted** | 11 | 0.03 | 1.03 | 0.93-1.13 | 0.60 |
| Eubacteriumbrachygroup | Simple mode | 11 | 0.09 | 1.09 | 0.85-1.39 | 0.50 |
| Eubacteriumbrachygroup | Weighted mode | 11 | 0.09 | 1.09 | 0.88-1.36 | 0.46 |
| FamilyXIIIAD3011group | MR Egger | 15 | -0.01 | 0.99 | 0.47-2.06 | 0.97 |
| FamilyXIIIAD3011group | Weighted median | 15 | -0.02 | 0.98 | 0.82-1.18 | 0.86 |
| FamilyXIIIAD3011group | **Inverse variance weighted** | 15 | -0.03 | 0.97 | 0.84-1.12 | 0.69 |
| FamilyXIIIAD3011group | Simple mode | 15 | 0.06 | 1.06 | 0.78-1.44 | 0.72 |
| FamilyXIIIAD3011group | Weighted mode | 15 | -0.11 | 0.90 | 0.66-1.21 | 0.48 |
| Lactococcus | MR Egger | 11 | 0.06 | 1.06 | 0.73-1.54 | 0.77 |
| Lactococcus | Weighted median | 11 | 0.09 | 1.09 | 0.98-1.21 | 0.11 |
| Lactococcus | **Inverse variance weighted** | 11 | 0.10 | 1.10 | 1.02-1.19 | 0.01 |
| Lactococcus | Simple mode | 11 | 0.10 | 1.10 | 0.93-1.31 | 0.28 |
| Lactococcus | Weighted mode | 11 | 0.08 | 1.09 | 0.93-1.27 | 0.31 |
| ChristensenellaceaeR.7group | MR Egger | 10 | -0.20 | 0.82 | 0.48-1.42 | 0.50 |
| ChristensenellaceaeR.7group | Weighted median | 10 | 0.02 | 1.02 | 0.8-1.31 | 0.85 |
| ChristensenellaceaeR.7group | **Inverse variance weighted** | 10 | 0.04 | 1.04 | 0.87-1.24 | 0.67 |
| ChristensenellaceaeR.7group | Simple mode | 10 | 0.05 | 1.05 | 0.7-1.56 | 0.83 |
| ChristensenellaceaeR.7group | Weighted mode | 10 | 0.03 | 1.04 | 0.67-1.59 | 0.88 |
| Eubacteriumnodatumgroup | MR Egger | 11 | -0.18 | 0.84 | 0.6-1.17 | 0.32 |
| Eubacteriumnodatumgroup | Weighted median | 11 | -0.02 | 0.98 | 0.89-1.09 | 0.74 |
| Eubacteriumnodatumgroup | **Inverse variance weighted** | 11 | -0.01 | 0.99 | 0.92-1.07 | 0.79 |
| Eubacteriumnodatumgroup | Simple mode | 11 | -0.03 | 0.97 | 0.82-1.16 | 0.77 |
| Eubacteriumnodatumgroup | Weighted mode | 11 | -0.03 | 0.97 | 0.83-1.14 | 0.72 |
| Clostridiumsensustricto1 | MR Egger | 9 | -0.06 | 0.94 | 0.67-1.34 | 0.76 |
| Clostridiumsensustricto1 | Weighted median | 9 | 0.05 | 1.05 | 0.87-1.27 | 0.60 |
| Clostridiumsensustricto1 | **Inverse variance weighted** | 9 | 0.04 | 1.04 | 0.9-1.21 | 0.59 |
| Clostridiumsensustricto1 | Simple mode | 9 | 0.08 | 1.08 | 0.81-1.44 | 0.61 |
| Clostridiumsensustricto1 | Weighted mode | 9 | 0.02 | 1.02 | 0.79-1.32 | 0.86 |
| RuminococcaceaeUCG003 | MR Egger | 14 | 0.28 | 1.32 | 0.87-1.99 | 0.21 |
| RuminococcaceaeUCG003 | Weighted median | 14 | 0.10 | 1.11 | 0.93-1.32 | 0.26 |
| RuminococcaceaeUCG003 | **Inverse variance weighted** | 14 | 0.06 | 1.06 | 0.93-1.21 | 0.37 |
| RuminococcaceaeUCG003 | Simple mode | 14 | 0.17 | 1.18 | 0.87-1.61 | 0.30 |
| RuminococcaceaeUCG003 | Weighted mode | 14 | 0.15 | 1.16 | 0.88-1.54 | 0.31 |
| Subdoligranulum | MR Egger | 13 | -0.12 | 0.89 | 0.58-1.37 | 0.61 |
| Subdoligranulum | Weighted median | 13 | -0.15 | 0.86 | 0.7-1.06 | 0.15 |
| Subdoligranulum | **Inverse variance weighted** | 13 | -0.05 | 0.95 | 0.81-1.11 | 0.52 |
| Subdoligranulum | Simple mode | 13 | -0.17 | 0.85 | 0.61-1.17 | 0.33 |
| Subdoligranulum | Weighted mode | 13 | -0.16 | 0.85 | 0.64-1.14 | 0.31 |
| Lachnospira | MR Egger | 7 | -0.01 | 0.99 | 0.3-3.26 | 0.99 |
| Lachnospira | Weighted median | 7 | -0.03 | 0.97 | 0.72-1.3 | 0.83 |
| Lachnospira | **Inverse variance weighted** | 7 | -0.13 | 0.88 | 0.71-1.09 | 0.23 |
| Lachnospira | Simple mode | 7 | -0.02 | 0.98 | 0.64-1.5 | 0.92 |
| Lachnospira | Weighted mode | 7 | -0.02 | 0.98 | 0.64-1.49 | 0.91 |
| Methanobrevibacter | MR Egger | 8 | -0.21 | 0.81 | 0.5-1.32 | 0.43 |
| Methanobrevibacter | Weighted median | 8 | 0.01 | 1.01 | 0.88-1.15 | 0.94 |
| Methanobrevibacter | **Inverse variance weighted** | 8 | -0.05 | 0.95 | 0.84-1.08 | 0.46 |
| Methanobrevibacter | Simple mode | 8 | 0.05 | 1.05 | 0.86-1.29 | 0.63 |
| Methanobrevibacter | Weighted mode | 8 | 0.04 | 1.04 | 0.87-1.26 | 0.66 |
| RuminococcaceaeUCG013 | MR Egger | 14 | 0.01 | 1.01 | 0.65-1.56 | 0.98 |
| RuminococcaceaeUCG013 | Weighted median | 14 | 0.00 | 1.00 | 0.82-1.22 | 1.00 |
| RuminococcaceaeUCG013 | **Inverse variance weighted** | 14 | -0.03 | 0.97 | 0.84-1.13 | 0.69 |
| RuminococcaceaeUCG013 | Simple mode | 14 | -0.02 | 0.98 | 0.7-1.38 | 0.93 |
| RuminococcaceaeUCG013 | Weighted mode | 14 | 0.01 | 1.01 | 0.74-1.39 | 0.93 |
| Odoribacter | MR Egger | 8 | 0.36 | 1.44 | 0.76-2.72 | 0.31 |
| Odoribacter | Weighted median | 8 | 0.25 | 1.28 | 1.01-1.64 | 0.04 |
| Odoribacter | **Inverse variance weighted** | 8 | 0.21 | 1.23 | 1.03-1.49 | 0.03 |
| Odoribacter | Simple mode | 8 | 0.25 | 1.29 | 0.88-1.87 | 0.23 |
| Odoribacter | Weighted mode | 8 | 0.25 | 1.29 | 0.9-1.85 | 0.21 |
| Tyzzerella3 | MR Egger | 14 | -0.33 | 0.72 | 0.43-1.18 | 0.21 |
| Tyzzerella3 | Weighted median | 14 | -0.06 | 0.94 | 0.84-1.05 | 0.29 |
| Tyzzerella3 | **Inverse variance weighted** | 14 | -0.06 | 0.94 | 0.87-1.02 | 0.15 |
| Tyzzerella3 | Simple mode | 14 | -0.08 | 0.93 | 0.76-1.13 | 0.46 |
| Tyzzerella3 | Weighted mode | 14 | -0.06 | 0.94 | 0.8-1.11 | 0.49 |
| Anaerostipes | MR Egger | 15 | -0.28 | 0.75 | 0.5-1.13 | 0.19 |
| Anaerostipes | Weighted median | 15 | -0.05 | 0.95 | 0.79-1.15 | 0.61 |
| Anaerostipes | **Inverse variance weighted** | 15 | -0.02 | 0.98 | 0.85-1.13 | 0.77 |
| Anaerostipes | Simple mode | 15 | -0.02 | 0.98 | 0.72-1.33 | 0.91 |
| Anaerostipes | Weighted mode | 15 | -0.04 | 0.96 | 0.73-1.27 | 0.80 |
| Enterorhabdus | MR Egger | 7 | -0.01 | 0.99 | 0.71-1.37 | 0.95 |
| Enterorhabdus | Weighted median | 7 | 0.11 | 1.12 | 0.95-1.31 | 0.18 |
| Enterorhabdus | **Inverse variance weighted** | 7 | 0.13 | 1.14 | 1-1.29 | 0.05 |
| Enterorhabdus | Simple mode | 7 | 0.04 | 1.04 | 0.82-1.32 | 0.75 |
| Enterorhabdus | Weighted mode | 7 | 0.11 | 1.11 | 0.9-1.37 | 0.35 |
| Erysipelatoclostridium | MR Egger | 16 | 0.33 | 1.39 | 0.92-2.09 | 0.14 |
| Erysipelatoclostridium | Weighted median | 16 | 0.06 | 1.06 | 0.92-1.22 | 0.44 |
| Erysipelatoclostridium | **Inverse variance weighted** | 16 | 0.02 | 1.02 | 0.92-1.13 | 0.71 |
| Erysipelatoclostridium | Simple mode | 16 | 0.05 | 1.05 | 0.82-1.33 | 0.71 |
| Erysipelatoclostridium | Weighted mode | 16 | 0.07 | 1.07 | 0.86-1.32 | 0.55 |
| RuminococcaceaeUCG014 | MR Egger | 18 | 0.26 | 1.29 | 0.91-1.83 | 0.17 |
| RuminococcaceaeUCG014 | Weighted median | 18 | 0.10 | 1.10 | 0.93-1.31 | 0.25 |
| RuminococcaceaeUCG014 | **Inverse variance weighted** | 18 | 0.11 | 1.12 | 0.99-1.26 | 0.07 |
| RuminococcaceaeUCG014 | Simple mode | 18 | 0.07 | 1.07 | 0.81-1.4 | 0.65 |
| RuminococcaceaeUCG014 | Weighted mode | 18 | 0.08 | 1.09 | 0.85-1.39 | 0.51 |
| LachnospiraceaeUCG008 | MR Egger | 12 | -0.19 | 0.83 | 0.48-1.42 | 0.51 |
| LachnospiraceaeUCG008 | Weighted median | 12 | -0.01 | 0.99 | 0.87-1.13 | 0.90 |
| LachnospiraceaeUCG008 | **Inverse variance weighted** | 12 | 0.03 | 1.03 | 0.93-1.14 | 0.54 |
| LachnospiraceaeUCG008 | Simple mode | 12 | -0.02 | 0.98 | 0.79-1.21 | 0.85 |
| LachnospiraceaeUCG008 | Weighted mode | 12 | -0.03 | 0.98 | 0.79-1.21 | 0.82 |
| Bacteroides | MR Egger | 12 | 0.11 | 1.12 | 0.43-2.91 | 0.83 |
| Bacteroides | Weighted median | 12 | -0.14 | 0.87 | 0.7-1.08 | 0.22 |
| Bacteroides | **Inverse variance weighted** | 12 | -0.15 | 0.86 | 0.72-1.02 | 0.09 |
| Bacteroides | Simple mode | 12 | -0.11 | 0.90 | 0.65-1.24 | 0.53 |
| Bacteroides | Weighted mode | 12 | -0.12 | 0.89 | 0.64-1.23 | 0.49 |
| Dorea | MR Egger | 12 | 0.14 | 1.15 | 0.73-1.81 | 0.57 |
| Dorea | Weighted median | 12 | 0.15 | 1.17 | 0.94-1.45 | 0.16 |
| Dorea | **Inverse variance weighted** | 12 | 0.04 | 1.04 | 0.88-1.22 | 0.64 |
| Dorea | Simple mode | 12 | 0.16 | 1.17 | 0.82-1.67 | 0.40 |
| Dorea | Weighted mode | 12 | 0.17 | 1.18 | 0.85-1.64 | 0.34 |
| Eubacteriumeligensgroup | MR Egger | 10 | 0.07 | 1.07 | 0.72-1.61 | 0.74 |
| Eubacteriumeligensgroup | Weighted median | 10 | 0.14 | 1.15 | 0.95-1.39 | 0.16 |
| Eubacteriumeligensgroup | **Inverse variance weighted** | 10 | 0.17 | 1.18 | 1.02-1.37 | 0.02 |
| Eubacteriumeligensgroup | Simple mode | 10 | 0.09 | 1.10 | 0.82-1.47 | 0.55 |
| Eubacteriumeligensgroup | Weighted mode | 10 | 0.10 | 1.10 | 0.88-1.39 | 0.42 |
| Clostridiuminnocuumgroup | MR Egger | 11 | -0.05 | 0.95 | 0.53-1.71 | 0.87 |
| Clostridiuminnocuumgroup | Weighted median | 11 | 0.00 | 1.00 | 0.89-1.13 | 0.97 |
| Clostridiuminnocuumgroup | **Inverse variance weighted** | 11 | 0.02 | 1.02 | 0.92-1.13 | 0.70 |
| Clostridiuminnocuumgroup | Simple mode | 11 | -0.11 | 0.90 | 0.73-1.11 | 0.35 |
| Clostridiuminnocuumgroup | Weighted mode | 11 | -0.09 | 0.92 | 0.75-1.12 | 0.40 |
| Holdemanella | MR Egger | 14 | 0.28 | 1.33 | 0.88-2.02 | 0.21 |
| Holdemanella | Weighted median | 14 | -0.06 | 0.95 | 0.82-1.09 | 0.45 |
| Holdemanella | **Inverse variance weighted** | 14 | -0.01 | 0.99 | 0.87-1.13 | 0.88 |
| Holdemanella | Simple mode | 14 | -0.09 | 0.91 | 0.71-1.18 | 0.51 |
| Holdemanella | Weighted mode | 14 | -0.07 | 0.94 | 0.73-1.2 | 0.61 |
| RuminococcaceaeUCG010 | MR Egger | 8 | 0.35 | 1.43 | 0.76-2.67 | 0.31 |
| RuminococcaceaeUCG010 | Weighted median | 8 | 0.17 | 1.19 | 0.96-1.47 | 0.11 |
| RuminococcaceaeUCG010 | **Inverse variance weighted** | 8 | 0.13 | 1.14 | 0.97-1.35 | 0.12 |
| RuminococcaceaeUCG010 | Simple mode | 8 | 0.19 | 1.21 | 0.87-1.67 | 0.30 |
| RuminococcaceaeUCG010 | Weighted mode | 8 | 0.19 | 1.21 | 0.88-1.67 | 0.28 |
| Butyricimonas | MR Egger | 16 | 0.04 | 1.04 | 0.68-1.57 | 0.87 |
| Butyricimonas | Weighted median | 16 | 0.08 | 1.08 | 0.93-1.27 | 0.30 |
| Butyricimonas | **Inverse variance weighted** | 16 | 0.05 | 1.05 | 0.94-1.18 | 0.39 |
| Butyricimonas | Simple mode | 16 | 0.11 | 1.12 | 0.86-1.47 | 0.42 |
| Butyricimonas | Weighted mode | 16 | 0.12 | 1.13 | 0.86-1.48 | 0.39 |
| Dialister | MR Egger | 12 | -0.27 | 0.76 | 0.38-1.54 | 0.47 |
| Dialister | Weighted median | 12 | -0.05 | 0.95 | 0.78-1.16 | 0.64 |
| Dialister | **Inverse variance weighted** | 12 | -0.11 | 0.89 | 0.77-1.04 | 0.15 |
| Dialister | Simple mode | 12 | 0.09 | 1.09 | 0.75-1.59 | 0.65 |
| Dialister | Weighted mode | 12 | 0.08 | 1.08 | 0.75-1.56 | 0.69 |
| RuminococcaceaeUCG002 | MR Egger | 24 | -0.16 | 0.85 | 0.61-1.18 | 0.35 |
| RuminococcaceaeUCG002 | Weighted median | 24 | -0.02 | 0.98 | 0.83-1.15 | 0.76 |
| RuminococcaceaeUCG002 | **Inverse variance weighted** | 24 | 0.00 | 1.00 | 0.88-1.12 | 0.95 |
| RuminococcaceaeUCG002 | Simple mode | 24 | 0.01 | 1.01 | 0.72-1.41 | 0.97 |
| RuminococcaceaeUCG002 | Weighted mode | 24 | -0.05 | 0.95 | 0.71-1.27 | 0.75 |
| LachnospiraceaeND3007group | MR Egger | 4 | -4.44 | 0.01 | 0-1.15 | 0.20 |
| LachnospiraceaeND3007group | Weighted median | 4 | -0.02 | 0.98 | 0.69-1.4 | 0.93 |
| LachnospiraceaeND3007group | **Inverse variance weighted** | 4 | -0.09 | 0.91 | 0.66-1.26 | 0.57 |
| LachnospiraceaeND3007group | Simple mode | 4 | -0.39 | 0.68 | 0.37-1.23 | 0.29 |
| LachnospiraceaeND3007group | Weighted mode | 4 | 0.11 | 1.11 | 0.73-1.69 | 0.66 |
| Sutterella | MR Egger | 12 | 0.54 | 1.72 | 0.86-3.45 | 0.16 |
| Sutterella | Weighted median | 12 | -0.02 | 0.98 | 0.8-1.2 | 0.86 |
| Sutterella | **Inverse variance weighted** | 12 | 0.03 | 1.03 | 0.88-1.19 | 0.74 |
| Sutterella | Simple mode | 12 | -0.07 | 0.94 | 0.65-1.35 | 0.73 |
| Sutterella | Weighted mode | 12 | -0.06 | 0.94 | 0.65-1.36 | 0.75 |
| Eubacteriumfissicatenagroup | MR Egger | 9 | -0.15 | 0.86 | 0.55-1.37 | 0.55 |
| Eubacteriumfissicatenagroup | Weighted median | 9 | 0.07 | 1.07 | 0.95-1.21 | 0.27 |
| Eubacteriumfissicatenagroup | **Inverse variance weighted** | 9 | 0.02 | 1.02 | 0.93-1.11 | 0.68 |
| Eubacteriumfissicatenagroup | Simple mode | 9 | 0.10 | 1.11 | 0.9-1.37 | 0.37 |
| Eubacteriumfissicatenagroup | Weighted mode | 9 | 0.10 | 1.11 | 0.91-1.34 | 0.35 |
| Peptococcus | MR Egger | 16 | -0.21 | 0.81 | 0.6-1.09 | 0.18 |
| Peptococcus | Weighted median | 16 | -0.03 | 0.97 | 0.88-1.08 | 0.63 |
| Peptococcus | **Inverse variance weighted** | 16 | -0.01 | 0.99 | 0.91-1.07 | 0.82 |
| Peptococcus | Simple mode | 16 | -0.05 | 0.95 | 0.79-1.14 | 0.59 |
| Peptococcus | Weighted mode | 16 | -0.04 | 0.96 | 0.8-1.14 | 0.63 |
| Coprococcus2 | MR Egger | 9 | 0.36 | 1.44 | 0.6-3.48 | 0.44 |
| Coprococcus2 | Weighted median | 9 | -0.07 | 0.94 | 0.76-1.15 | 0.53 |
| Coprococcus2 | **Inverse variance weighted** | 9 | -0.06 | 0.95 | 0.81-1.1 | 0.48 |
| Coprococcus2 | Simple mode | 9 | -0.07 | 0.93 | 0.69-1.26 | 0.65 |
| Coprococcus2 | Weighted mode | 9 | -0.06 | 0.94 | 0.69-1.29 | 0.73 |
| RuminococcaceaeNK4A214group | MR Egger | 16 | 0.10 | 1.11 | 0.75-1.64 | 0.61 |
| RuminococcaceaeNK4A214group | Weighted median | 16 | 0.00 | 1.00 | 0.84-1.2 | 0.97 |
| RuminococcaceaeNK4A214group | **Inverse variance weighted** | 16 | 0.06 | 1.06 | 0.93-1.2 | 0.38 |
| RuminococcaceaeNK4A214group | Simple mode | 16 | -0.05 | 0.95 | 0.7-1.3 | 0.76 |
| RuminococcaceaeNK4A214group | Weighted mode | 16 | -0.02 | 0.98 | 0.71-1.35 | 0.90 |
| RuminococcaceaeUCG011 | MR Egger | 8 | -0.11 | 0.89 | 0.57-1.41 | 0.65 |
| RuminococcaceaeUCG011 | Weighted median | 8 | 0.01 | 1.01 | 0.9-1.13 | 0.88 |
| RuminococcaceaeUCG011 | **Inverse variance weighted** | 8 | 0.02 | 1.02 | 0.93-1.11 | 0.71 |
| RuminococcaceaeUCG011 | Simple mode | 8 | 0.03 | 1.03 | 0.86-1.22 | 0.78 |
| RuminococcaceaeUCG011 | Weighted mode | 8 | 0.02 | 1.02 | 0.86-1.22 | 0.80 |
| LachnospiraceaeUCG010 | MR Egger | 12 | -0.21 | 0.81 | 0.55-1.18 | 0.30 |
| LachnospiraceaeUCG010 | Weighted median | 12 | -0.10 | 0.91 | 0.75-1.1 | 0.31 |
| LachnospiraceaeUCG010 | **Inverse variance weighted** | 12 | -0.02 | 0.98 | 0.86-1.13 | 0.79 |
| LachnospiraceaeUCG010 | Simple mode | 12 | -0.13 | 0.88 | 0.63-1.23 | 0.48 |
| LachnospiraceaeUCG010 | Weighted mode | 12 | -0.12 | 0.88 | 0.68-1.16 | 0.39 |
| Alloprevotella | MR Egger | 7 | -0.50 | 0.61 | 0.26-1.41 | 0.30 |
| Alloprevotella | Weighted median | 7 | -0.10 | 0.91 | 0.8-1.03 | 0.14 |
| Alloprevotella | **Inverse variance weighted** | 7 | -0.08 | 0.92 | 0.84-1.01 | 0.08 |
| Alloprevotella | Simple mode | 7 | -0.10 | 0.90 | 0.76-1.07 | 0.29 |
| Alloprevotella | Weighted mode | 7 | -0.10 | 0.90 | 0.75-1.08 | 0.32 |
| Eubacteriumruminantiumgroup | MR Egger | 19 | 0.20 | 1.22 | 0.91-1.65 | 0.21 |
| Eubacteriumruminantiumgroup | Weighted median | 19 | 0.09 | 1.09 | 0.96-1.24 | 0.17 |
| Eubacteriumruminantiumgroup | **Inverse variance weighted** | 19 | 0.01 | 1.01 | 0.92-1.1 | 0.87 |
| Eubacteriumruminantiumgroup | Simple mode | 19 | 0.15 | 1.16 | 0.89-1.5 | 0.28 |
| Eubacteriumruminantiumgroup | Weighted mode | 19 | 0.16 | 1.17 | 0.92-1.48 | 0.21 |
| Barnesiella | MR Egger | 16 | -0.34 | 0.71 | 0.44-1.14 | 0.18 |
| Barnesiella | Weighted median | 16 | 0.05 | 1.05 | 0.89-1.24 | 0.58 |
| Barnesiella | **Inverse variance weighted** | 16 | -0.01 | 0.99 | 0.87-1.13 | 0.91 |
| Barnesiella | Simple mode | 16 | 0.10 | 1.11 | 0.8-1.53 | 0.54 |
| Barnesiella | Weighted mode | 16 | 0.09 | 1.10 | 0.82-1.46 | 0.54 |
| Ruminococcusgauvreauiigroup | MR Egger | 13 | -0.59 | 0.55 | 0.3-1.03 | 0.09 |
| Ruminococcusgauvreauiigroup | Weighted median | 13 | -0.14 | 0.87 | 0.72-1.06 | 0.16 |
| Ruminococcusgauvreauiigroup | **Inverse variance weighted** | 13 | -0.20 | 0.82 | 0.71-0.94 | 0.00 |
| Ruminococcusgauvreauiigroup | Simple mode | 13 | -0.10 | 0.90 | 0.65-1.25 | 0.54 |
| Ruminococcusgauvreauiigroup | Weighted mode | 13 | -0.11 | 0.90 | 0.68-1.19 | 0.47 |
| Coprococcus3 | MR Egger | 9 | 0.04 | 1.04 | 0.52-2.1 | 0.91 |
| Coprococcus3 | Weighted median | 9 | 0.14 | 1.15 | 0.91-1.44 | 0.24 |
| Coprococcus3 | **Inverse variance weighted** | 9 | 0.15 | 1.16 | 0.97-1.39 | 0.11 |
| Coprococcus3 | Simple mode | 9 | 0.13 | 1.14 | 0.82-1.59 | 0.46 |
| Coprococcus3 | Weighted mode | 9 | 0.13 | 1.14 | 0.83-1.56 | 0.43 |
| Catenibacterium | MR Egger | 5 | -0.71 | 0.49 | 0.17-1.44 | 0.29 |
| Catenibacterium | Weighted median | 5 | 0.05 | 1.05 | 0.9-1.22 | 0.55 |
| Catenibacterium | **Inverse variance weighted** | 5 | 0.04 | 1.04 | 0.92-1.17 | 0.57 |
| Catenibacterium | Simple mode | 5 | 0.04 | 1.05 | 0.86-1.27 | 0.68 |
| Catenibacterium | Weighted mode | 5 | 0.04 | 1.05 | 0.87-1.26 | 0.66 |
| Ruminococcus2 | MR Egger | 15 | -0.09 | 0.92 | 0.68-1.24 | 0.58 |
| Ruminococcus2 | Weighted median | 15 | 0.05 | 1.05 | 0.88-1.26 | 0.58 |
| Ruminococcus2 | **Inverse variance weighted** | 15 | 0.04 | 1.04 | 0.92-1.18 | 0.55 |
| Ruminococcus2 | Simple mode | 15 | -0.12 | 0.89 | 0.66-1.2 | 0.46 |
| Ruminococcus2 | Weighted mode | 15 | 0.06 | 1.06 | 0.82-1.38 | 0.65 |
| Intestinimonas | MR Egger | 20 | 0.05 | 1.05 | 0.75-1.47 | 0.78 |
| Intestinimonas | Weighted median | 20 | 0.03 | 1.03 | 0.89-1.19 | 0.67 |
| Intestinimonas | **Inverse variance weighted** | 20 | 0.09 | 1.10 | 0.97-1.25 | 0.15 |
| Intestinimonas | Simple mode | 20 | 0.03 | 1.03 | 0.8-1.33 | 0.83 |
| Intestinimonas | Weighted mode | 20 | 0.02 | 1.02 | 0.82-1.26 | 0.88 |
| CandidatusSoleaferrea | MR Egger | 13 | 0.25 | 1.29 | 0.92-1.8 | 0.16 |
| CandidatusSoleaferrea | Weighted median | 13 | 0.08 | 1.09 | 0.95-1.24 | 0.24 |
| CandidatusSoleaferrea | **Inverse variance weighted** | 13 | 0.02 | 1.02 | 0.93-1.13 | 0.62 |
| CandidatusSoleaferrea | Simple mode | 13 | 0.11 | 1.12 | 0.9-1.39 | 0.33 |
| CandidatusSoleaferrea | Weighted mode | 13 | 0.12 | 1.13 | 0.92-1.39 | 0.26 |
| RuminococcaceaeUCG004 | MR Egger | 12 | 0.47 | 1.61 | 0.76-3.39 | 0.24 |
| RuminococcaceaeUCG004 | Weighted median | 12 | -0.02 | 0.98 | 0.82-1.18 | 0.85 |
| RuminococcaceaeUCG004 | **Inverse variance weighted** | 12 | 0.00 | 1.00 | 0.88-1.13 | 0.96 |
| RuminococcaceaeUCG004 | Simple mode | 12 | 0.01 | 1.01 | 0.74-1.37 | 0.97 |
| RuminococcaceaeUCG004 | Weighted mode | 12 | -0.04 | 0.96 | 0.72-1.29 | 0.81 |
| Senegalimassilia | MR Egger | 5 | 0.08 | 1.09 | 0.49-2.43 | 0.85 |
| Senegalimassilia | Weighted median | 5 | 0.10 | 1.10 | 0.89-1.36 | 0.37 |
| Senegalimassilia | **Inverse variance weighted** | 5 | 0.01 | 1.01 | 0.82-1.23 | 0.93 |
| Senegalimassilia | Simple mode | 5 | 0.11 | 1.12 | 0.87-1.45 | 0.44 |
| Senegalimassilia | Weighted mode | 5 | 0.12 | 1.13 | 0.86-1.48 | 0.43 |
| Olsenella | MR Egger | 11 | 0.22 | 1.25 | 0.83-1.88 | 0.31 |
| Olsenella | Weighted median | 11 | -0.09 | 0.92 | 0.82-1.03 | 0.15 |
| Olsenella | **Inverse variance weighted** | 11 | -0.04 | 0.97 | 0.86-1.08 | 0.53 |
| Olsenella | Simple mode | 11 | -0.13 | 0.88 | 0.72-1.06 | 0.21 |
| Olsenella | Weighted mode | 11 | -0.13 | 0.88 | 0.74-1.05 | 0.19 |
| RuminococcaceaeUCG009 | MR Egger | 14 | -0.12 | 0.88 | 0.59-1.33 | 0.57 |
| RuminococcaceaeUCG009 | Weighted median | 14 | -0.02 | 0.98 | 0.86-1.12 | 0.77 |
| RuminococcaceaeUCG009 | **Inverse variance weighted** | 14 | -0.02 | 0.98 | 0.89-1.08 | 0.66 |
| RuminococcaceaeUCG009 | Simple mode | 14 | 0.02 | 1.02 | 0.81-1.3 | 0.85 |
| RuminococcaceaeUCG009 | Weighted mode | 14 | 0.01 | 1.01 | 0.79-1.3 | 0.92 |
| Phascolarctobacterium | MR Egger | 12 | -0.31 | 0.74 | 0.45-1.2 | 0.25 |
| Phascolarctobacterium | Weighted median | 12 | 0.01 | 1.01 | 0.86-1.19 | 0.89 |
| Phascolarctobacterium | **Inverse variance weighted** | 12 | -0.03 | 0.97 | 0.85-1.11 | 0.66 |
| Phascolarctobacterium | Simple mode | 12 | 0.04 | 1.04 | 0.8-1.34 | 0.79 |
| Phascolarctobacterium | Weighted mode | 12 | 0.04 | 1.04 | 0.8-1.35 | 0.78 |
| Fusicatenibacter | MR Egger | 18 | 0.08 | 1.08 | 0.65-1.8 | 0.77 |
| Fusicatenibacter | Weighted median | 18 | 0.03 | 1.03 | 0.86-1.22 | 0.76 |
| Fusicatenibacter | **Inverse variance weighted** | 18 | 0.03 | 1.03 | 0.9-1.18 | 0.65 |
| Fusicatenibacter | Simple mode | 18 | 0.06 | 1.06 | 0.79-1.44 | 0.69 |
| Fusicatenibacter | Weighted mode | 18 | 0.07 | 1.07 | 0.77-1.48 | 0.70 |
| Anaerotruncus | MR Egger | 15 | -0.08 | 0.92 | 0.6-1.42 | 0.71 |
| Anaerotruncus | Weighted median | 15 | -0.13 | 0.88 | 0.73-1.07 | 0.20 |
| Anaerotruncus | **Inverse variance weighted** | 15 | -0.01 | 0.99 | 0.86-1.13 | 0.84 |
| Anaerotruncus | Simple mode | 15 | -0.18 | 0.84 | 0.63-1.11 | 0.23 |
| Anaerotruncus | Weighted mode | 15 | -0.16 | 0.85 | 0.66-1.1 | 0.24 |
| Howardella | MR Egger | 11 | -0.04 | 0.97 | 0.69-1.36 | 0.84 |
| Howardella | Weighted median | 11 | 0.18 | 1.20 | 1.08-1.33 | 0.00 |
| Howardella | **Inverse variance weighted** | 11 | 0.17 | 1.18 | 1.09-1.28 | 0.00 |
| Howardella | Simple mode | 11 | 0.22 | 1.25 | 1.03-1.51 | 0.04 |
| Howardella | Weighted mode | 11 | 0.21 | 1.23 | 1.02-1.49 | 0.05 |
| Gordonibacter | MR Egger | 14 | -0.14 | 0.87 | 0.6-1.28 | 0.50 |
| Gordonibacter | Weighted median | 14 | 0.01 | 1.01 | 0.91-1.12 | 0.85 |
| Gordonibacter | **Inverse variance weighted** | 14 | -0.03 | 0.97 | 0.88-1.07 | 0.55 |
| Gordonibacter | Simple mode | 14 | 0.07 | 1.07 | 0.89-1.29 | 0.48 |
| Gordonibacter | Weighted mode | 14 | 0.05 | 1.05 | 0.88-1.25 | 0.60 |
| Coprobacter | MR Egger | 14 | -0.05 | 0.95 | 0.72-1.26 | 0.74 |
| Coprobacter | Weighted median | 14 | -0.05 | 0.95 | 0.84-1.08 | 0.42 |
| Coprobacter | **Inverse variance weighted** | 14 | -0.03 | 0.97 | 0.89-1.06 | 0.53 |
| Coprobacter | Simple mode | 14 | -0.04 | 0.96 | 0.79-1.17 | 0.70 |
| Coprobacter | Weighted mode | 14 | -0.04 | 0.96 | 0.78-1.17 | 0.69 |
| Eubacteriumventriosumgroup | MR Egger | 15 | 0.07 | 1.07 | 0.57-2.02 | 0.84 |
| Eubacteriumventriosumgroup | Weighted median | 15 | 0.05 | 1.05 | 0.86-1.27 | 0.64 |
| Eubacteriumventriosumgroup | **Inverse variance weighted** | 15 | 0.08 | 1.08 | 0.94-1.24 | 0.30 |
| Eubacteriumventriosumgroup | Simple mode | 15 | -0.01 | 0.99 | 0.69-1.42 | 0.94 |
| Eubacteriumventriosumgroup | Weighted mode | 15 | -0.01 | 0.99 | 0.71-1.38 | 0.94 |
| Allisonella | MR Egger | 9 | -0.34 | 0.71 | 0.42-1.21 | 0.25 |
| Allisonella | Weighted median | 9 | 0.00 | 1.00 | 0.9-1.11 | 0.94 |
| Allisonella | **Inverse variance weighted** | 9 | -0.01 | 0.99 | 0.91-1.07 | 0.78 |
| Allisonella | Simple mode | 9 | 0.01 | 1.01 | 0.86-1.19 | 0.91 |
| Allisonella | Weighted mode | 9 | 0.01 | 1.01 | 0.87-1.18 | 0.86 |
| Bifidobacterium | MR Egger | 15 | -0.13 | 0.88 | 0.62-1.24 | 0.49 |
| Bifidobacterium | Weighted median | 15 | -0.08 | 0.92 | 0.78-1.1 | 0.36 |
| Bifidobacterium | **Inverse variance weighted** | 15 | -0.08 | 0.92 | 0.82-1.04 | 0.20 |
| Bifidobacterium | Simple mode | 15 | -0.09 | 0.91 | 0.68-1.23 | 0.55 |
| Bifidobacterium | Weighted mode | 15 | -0.10 | 0.90 | 0.7-1.16 | 0.44 |
| Hungatella | MR Egger | 5 | -0.02 | 0.98 | 0.44-2.19 | 0.97 |
| Hungatella | Weighted median | 5 | -0.03 | 0.97 | 0.83-1.14 | 0.72 |
| Hungatella | **Inverse variance weighted** | 5 | -0.01 | 0.99 | 0.87-1.12 | 0.86 |
| Hungatella | Simple mode | 5 | -0.03 | 0.97 | 0.78-1.21 | 0.79 |
| Hungatella | Weighted mode | 5 | -0.03 | 0.97 | 0.78-1.2 | 0.78 |
| Terrisporobacter | MR Egger | 5 | -0.08 | 0.92 | 0.51-1.67 | 0.80 |
| Terrisporobacter | Weighted median | 5 | -0.01 | 0.99 | 0.82-1.2 | 0.93 |
| Terrisporobacter | **Inverse variance weighted** | 5 | -0.09 | 0.91 | 0.75-1.11 | 0.37 |
| Terrisporobacter | Simple mode | 5 | 0.09 | 1.10 | 0.83-1.45 | 0.55 |
| Terrisporobacter | Weighted mode | 5 | 0.09 | 1.10 | 0.83-1.45 | 0.56 |
| Ruminococcusgnavusgroup | MR Egger | 12 | -0.33 | 0.72 | 0.39-1.34 | 0.32 |
| Ruminococcusgnavusgroup | Weighted median | 12 | 0.01 | 1.01 | 0.88-1.16 | 0.89 |
| Ruminococcusgnavusgroup | **Inverse variance weighted** | 12 | 0.00 | 1.00 | 0.88-1.14 | 0.99 |
| Ruminococcusgnavusgroup | Simple mode | 12 | 0.01 | 1.01 | 0.8-1.27 | 0.94 |
| Ruminococcusgnavusgroup | Weighted mode | 12 | 0.02 | 1.02 | 0.81-1.27 | 0.88 |
| Eubacteriumcoprostanoligenesgroup | MR Egger | 15 | 0.08 | 1.08 | 0.49-2.4 | 0.85 |
| Eubacteriumcoprostanoligenesgroup | Weighted median | 15 | -0.07 | 0.93 | 0.75-1.14 | 0.48 |
| Eubacteriumcoprostanoligenesgroup | **Inverse variance weighted** | 15 | -0.09 | 0.91 | 0.75-1.1 | 0.33 |
| Eubacteriumcoprostanoligenesgroup | Simple mode | 15 | -0.10 | 0.90 | 0.62-1.3 | 0.59 |
| Eubacteriumcoprostanoligenesgroup | Weighted mode | 15 | -0.09 | 0.91 | 0.66-1.25 | 0.57 |
| Ruminiclostridium6 | MR Egger | 16 | -0.15 | 0.86 | 0.64-1.16 | 0.34 |
| Ruminiclostridium6 | Weighted median | 16 | 0.06 | 1.06 | 0.9-1.25 | 0.46 |
| Ruminiclostridium6 | **Inverse variance weighted** | 16 | 0.03 | 1.03 | 0.92-1.16 | 0.58 |
| Ruminiclostridium6 | Simple mode | 16 | 0.11 | 1.12 | 0.85-1.47 | 0.42 |
| Ruminiclostridium6 | Weighted mode | 16 | 0.05 | 1.05 | 0.8-1.37 | 0.72 |
| Coprococcus1 | MR Egger | 13 | 0.16 | 1.18 | 0.82-1.69 | 0.39 |
| Coprococcus1 | Weighted median | 13 | -0.02 | 0.98 | 0.81-1.19 | 0.84 |
| Coprococcus1 | **Inverse variance weighted** | 13 | 0.00 | 1.00 | 0.87-1.15 | 0.98 |
| Coprococcus1 | Simple mode | 13 | -0.07 | 0.93 | 0.66-1.33 | 0.71 |
| Coprococcus1 | Weighted mode | 13 | -0.06 | 0.94 | 0.69-1.28 | 0.70 |
| Oscillibacter | MR Egger | 17 | -0.01 | 0.99 | 0.63-1.55 | 0.96 |
| Oscillibacter | Weighted median | 17 | -0.01 | 0.99 | 0.86-1.15 | 0.95 |
| Oscillibacter | **Inverse variance weighted** | 17 | 0.03 | 1.03 | 0.92-1.16 | 0.60 |
| Oscillibacter | Simple mode | 17 | -0.04 | 0.96 | 0.72-1.28 | 0.78 |
| Oscillibacter | Weighted mode | 17 | -0.07 | 0.93 | 0.7-1.25 | 0.65 |
| Parasutterella | MR Egger | 17 | 0.22 | 1.25 | 0.89-1.74 | 0.22 |
| Parasutterella | Weighted median | 17 | 0.11 | 1.12 | 0.97-1.29 | 0.13 |
| Parasutterella | **Inverse variance weighted** | 17 | 0.08 | 1.08 | 0.97-1.2 | 0.14 |
| Parasutterella | Simple mode | 17 | 0.11 | 1.11 | 0.87-1.41 | 0.41 |
| Parasutterella | Weighted mode | 17 | 0.13 | 1.14 | 0.91-1.41 | 0.27 |
| Lachnoclostridium | MR Egger | 13 | 0.16 | 1.18 | 0.67-2.07 | 0.58 |
| Lachnoclostridium | Weighted median | 13 | 0.14 | 1.15 | 0.93-1.41 | 0.20 |
| Lachnoclostridium | **Inverse variance weighted** | 13 | 0.12 | 1.13 | 0.97-1.32 | 0.12 |
| Lachnoclostridium | Simple mode | 13 | 0.19 | 1.21 | 0.85-1.71 | 0.31 |
| Lachnoclostridium | Weighted mode | 13 | 0.19 | 1.21 | 0.85-1.72 | 0.31 |
| Holdemania | MR Egger | 18 | -0.07 | 0.93 | 0.71-1.21 | 0.60 |
| Holdemania | Weighted median | 18 | -0.04 | 0.96 | 0.85-1.08 | 0.48 |
| Holdemania | **Inverse variance weighted** | 18 | -0.02 | 0.98 | 0.9-1.08 | 0.70 |
| Holdemania | Simple mode | 18 | 0.10 | 1.11 | 0.89-1.38 | 0.38 |
| Holdemania | Weighted mode | 18 | -0.07 | 0.94 | 0.78-1.12 | 0.48 |
| Eubacteriumhalliigroup | MR Egger | 16 | 0.06 | 1.06 | 0.83-1.35 | 0.65 |
| Eubacteriumhalliigroup | Weighted median | 16 | 0.08 | 1.09 | 0.93-1.27 | 0.29 |
| Eubacteriumhalliigroup | **Inverse variance weighted** | 16 | 0.03 | 1.03 | 0.91-1.16 | 0.66 |
| Eubacteriumhalliigroup | Simple mode | 16 | 0.09 | 1.10 | 0.85-1.42 | 0.49 |
| Eubacteriumhalliigroup | Weighted mode | 16 | 0.12 | 1.12 | 0.88-1.43 | 0.36 |
| Eubacteriumrectalegroup | MR Egger | 12 | -0.18 | 0.83 | 0.52-1.32 | 0.46 |
| Eubacteriumrectalegroup | Weighted median | 12 | 0.08 | 1.08 | 0.88-1.34 | 0.46 |
| Eubacteriumrectalegroup | **Inverse variance weighted** | 12 | -0.01 | 0.99 | 0.85-1.16 | 0.93 |
| Eubacteriumrectalegroup | Simple mode | 12 | 0.06 | 1.06 | 0.76-1.5 | 0.73 |
| Eubacteriumrectalegroup | Weighted mode | 12 | 0.07 | 1.07 | 0.78-1.48 | 0.67 |
| Veillonella | MR Egger | 11 | -0.66 | 0.52 | 0.21-1.24 | 0.17 |
| Veillonella | Weighted median | 11 | -0.04 | 0.96 | 0.79-1.16 | 0.66 |
| Veillonella | **Inverse variance weighted** | 11 | 0.00 | 1.00 | 0.86-1.17 | 0.96 |
| Veillonella | Simple mode | 11 | -0.06 | 0.94 | 0.69-1.28 | 0.72 |
| Veillonella | Weighted mode | 11 | -0.07 | 0.94 | 0.68-1.29 | 0.70 |
| Actinomyces | MR Egger | 8 | 0.05 | 1.05 | 0.71-1.55 | 0.83 |
| Actinomyces | Weighted median | 8 | 0.07 | 1.07 | 0.9-1.26 | 0.44 |
| Actinomyces | **Inverse variance weighted** | 8 | 0.09 | 1.09 | 0.96-1.24 | 0.20 |
| Actinomyces | Simple mode | 8 | 0.20 | 1.22 | 0.96-1.56 | 0.16 |
| Actinomyces | Weighted mode | 8 | 0.09 | 1.10 | 0.87-1.37 | 0.45 |
| LachnospiraceaeUCG004 | MR Egger | 13 | -0.08 | 0.92 | 0.4-2.1 | 0.85 |
| LachnospiraceaeUCG004 | Weighted median | 13 | 0.00 | 1.00 | 0.8-1.25 | 1.00 |
| LachnospiraceaeUCG004 | **Inverse variance weighted** | 13 | -0.06 | 0.94 | 0.77-1.15 | 0.56 |
| LachnospiraceaeUCG004 | Simple mode | 13 | 0.01 | 1.01 | 0.68-1.49 | 0.96 |
| LachnospiraceaeUCG004 | Weighted mode | 13 | 0.01 | 1.01 | 0.67-1.53 | 0.95 |
| Eubacteriumoxidoreducensgroup | MR Egger | 6 | 0.03 | 1.03 | 0.67-1.58 | 0.90 |
| Eubacteriumoxidoreducensgroup | Weighted median | 6 | 0.03 | 1.03 | 0.89-1.19 | 0.67 |
| Eubacteriumoxidoreducensgroup | **Inverse variance weighted** | 6 | 0.03 | 1.03 | 0.91-1.17 | 0.61 |
| Eubacteriumoxidoreducensgroup | Simple mode | 6 | 0.03 | 1.03 | 0.83-1.27 | 0.80 |
| Eubacteriumoxidoreducensgroup | Weighted mode | 6 | 0.03 | 1.03 | 0.84-1.26 | 0.77 |
| Ruminococcus1 | MR Egger | 13 | -0.05 | 0.95 | 0.61-1.5 | 0.84 |
| Ruminococcus1 | Weighted median | 13 | 0.08 | 1.08 | 0.88-1.32 | 0.47 |
| Ruminococcus1 | **Inverse variance weighted** | 13 | 0.03 | 1.03 | 0.88-1.21 | 0.71 |
| Ruminococcus1 | Simple mode | 13 | 0.16 | 1.18 | 0.81-1.72 | 0.41 |
| Ruminococcus1 | Weighted mode | 13 | 0.14 | 1.15 | 0.8-1.66 | 0.45 |
| Prevotella9 | MR Egger | 18 | -0.19 | 0.82 | 0.64-1.06 | 0.16 |
| Prevotella9 | Weighted median | 18 | 0.00 | 1.00 | 0.88-1.14 | 1.00 |
| Prevotella9 | **Inverse variance weighted** | 18 | 0.03 | 1.03 | 0.93-1.13 | 0.59 |
| Prevotella9 | Simple mode | 18 | 0.01 | 1.01 | 0.8-1.27 | 0.95 |
| Prevotella9 | Weighted mode | 18 | -0.02 | 0.98 | 0.8-1.2 | 0.83 |

MR, Mendelian randomization; CRF, chronic renal failure; SNP, single nucleotide polymorphism; OR, odds ratio; CI, confidence interval.

**Supplementary Tables 3** Directional horizontal pleiotropy assessed by intercept term in MR Egger regression of the association between gut microbiota and CRF.

| **Exposure(Bacterial taxa)** | **Egger_intercept** | **SE** | **P-value** |
| --- | --- | --- | --- |
| Ruminiclostridium9 | 0.02 | 0.03 | 0.50 |
| Lactobacillus | 0.00 | 0.02 | 0.82 |
| Streptococcus | 0.03 | 0.02 | 0.27 |
| Ruminiclostridium5 | 0.02 | 0.01 | 0.21 |
| Butyricicoccus | -0.02 | 0.01 | 0.22 |
| Victivallis | 0.01 | 0.03 | 0.80 |
| ErysipelotrichaceaeUCG003 | 0.01 | 0.02 | 0.60 |
| Haemophilus | 0.04 | 0.02 | 0.03 |
| Blautia | 0.00 | 0.01 | 0.87 |
| Oxalobacter | -0.01 | 0.03 | 0.85 |
| Anaerofilum | -0.01 | 0.02 | 0.77 |
| LachnospiraceaeNK4A136group | -0.01 | 0.01 | 0.21 |
| Eisenbergiella | 0.06 | 0.04 | 0.12 |
| Prevotella7 | 0.00 | 0.05 | 0.97 |
| Eggerthella | -0.02 | 0.03 | 0.39 |
| FamilyXIIIUCG001 | 0.02 | 0.03 | 0.51 |
| RikenellaceaeRC9gutgroup | 0.01 | 0.03 | 0.85 |
| Paraprevotella | -0.01 | 0.02 | 0.64 |
| Parabacteroides | 0.02 | 0.02 | 0.37 |
| Ruminococcustorquesgroup | -0.01 | 0.02 | 0.79 |
| Alistipes | 0.00 | 0.03 | 0.93 |
| Escherichia.Shigella | 0.02 | 0.02 | 0.16 |
| Desulfovibrio | -0.01 | 0.02 | 0.66 |
| Adlercreutzia | 0.01 | 0.03 | 0.59 |
| LachnospiraceaeFCS020group | 0.02 | 0.01 | 0.20 |
| Flavonifractor | -0.03 | 0.03 | 0.41 |
| Sellimonas | -0.05 | 0.03 | 0.20 |
| Romboutsia | 0.02 | 0.02 | 0.33 |
| Marvinbryantia | -0.02 | 0.03 | 0.51 |
| Collinsella | 0.04 | 0.03 | 0.18 |
| Faecalibacterium | -0.01 | 0.01 | 0.33 |
| Oscillospira | 0.05 | 0.03 | 0.12 |
| Roseburia | 0.00 | 0.02 | 0.83 |
| Intestinibacter | 0.01 | 0.02 | 0.66 |
| Butyrivibrio | 0.03 | 0.02 | 0.12 |
| Turicibacter | 0.00 | 0.02 | 0.91 |
| DefluviitaleaceaeUCG011 | 0.05 | 0.03 | 0.16 |
| LachnospiraceaeUCG001 | -0.04 | 0.02 | 0.13 |
| Eubacteriumxylanophilumgroup | -0.02 | 0.02 | 0.39 |
| Akkermansia | 0.01 | 0.02 | 0.78 |
| LachnospiraceaeNC2004group | 0.02 | 0.03 | 0.39 |
| RuminococcaceaeUCG005 | 0.04 | 0.02 | 0.04 |
| Bilophila | -0.02 | 0.02 | 0.49 |
| Slackia | 0.01 | 0.03 | 0.68 |
| Eubacteriumbrachygroup | 0.00 | 0.02 | 0.86 |
| FamilyXIIIAD3011group | 0.00 | 0.03 | 0.96 |
| Lactococcus | 0.01 | 0.03 | 0.83 |
| ChristensenellaceaeR.7group | 0.02 | 0.02 | 0.39 |
| Eubacteriumnodatumgroup | 0.03 | 0.02 | 0.34 |
| Clostridiumsensustricto1 | 0.01 | 0.02 | 0.56 |
| RuminococcaceaeUCG003 | -0.02 | 0.02 | 0.30 |
| Subdoligranulum | 0.01 | 0.02 | 0.76 |
| Lachnospira | -0.01 | 0.04 | 0.85 |
| Methanobrevibacter | 0.02 | 0.03 | 0.52 |
| RuminococcaceaeUCG013 | 0.00 | 0.02 | 0.86 |
| Odoribacter | -0.01 | 0.02 | 0.64 |
| Tyzzerella3 | 0.04 | 0.04 | 0.30 |
| Anaerostipes | 0.02 | 0.01 | 0.19 |
| Enterorhabdus | 0.02 | 0.02 | 0.41 |
| Erysipelatoclostridium | -0.03 | 0.02 | 0.15 |
| RuminococcaceaeUCG014 | -0.01 | 0.01 | 0.39 |
| LachnospiraceaeUCG008 | 0.02 | 0.03 | 0.44 |
| Bacteroides | -0.02 | 0.03 | 0.60 |
| Dorea | -0.01 | 0.02 | 0.66 |
| Eubacteriumeligensgroup | 0.01 | 0.02 | 0.63 |
| Clostridiuminnocuumgroup | 0.01 | 0.04 | 0.81 |
| Holdemanella | -0.03 | 0.02 | 0.17 |
| RuminococcaceaeUCG010 | -0.02 | 0.02 | 0.50 |
| Butyricimonas | 0.00 | 0.02 | 0.95 |
| Dialister | 0.01 | 0.03 | 0.66 |
| RuminococcaceaeUCG002 | 0.01 | 0.01 | 0.33 |
| LachnospiraceaeND3007group | 0.26 | 0.14 | 0.20 |
| Sutterella | -0.03 | 0.02 | 0.17 |
| Eubacteriumfissicatenagroup | 0.02 | 0.03 | 0.49 |
| Peptococcus | 0.03 | 0.02 | 0.19 |
| Coprococcus2 | -0.03 | 0.03 | 0.37 |
| RuminococcaceaeNK4A214group | 0.00 | 0.02 | 0.81 |
| RuminococcaceaeUCG011 | 0.02 | 0.03 | 0.60 |
| LachnospiraceaeUCG010 | 0.02 | 0.02 | 0.31 |
| Alloprevotella | 0.06 | 0.06 | 0.37 |
| Eubacteriumruminantiumgroup | -0.02 | 0.01 | 0.20 |
| Barnesiella | 0.03 | 0.02 | 0.17 |
| Ruminococcusgauvreauiigroup | 0.03 | 0.02 | 0.23 |
| Coprococcus3 | 0.01 | 0.02 | 0.77 |
| Catenibacterium | 0.10 | 0.07 | 0.26 |
| Ruminococcus2 | 0.01 | 0.01 | 0.39 |
| Intestinimonas | 0.00 | 0.02 | 0.77 |
| CandidatusSoleaferrea | -0.03 | 0.02 | 0.19 |
| RuminococcaceaeUCG004 | -0.04 | 0.03 | 0.23 |
| Senegalimassilia | -0.01 | 0.04 | 0.86 |
| Olsenella | -0.03 | 0.03 | 0.23 |
| RuminococcaceaeUCG009 | 0.01 | 0.02 | 0.63 |
| Phascolarctobacterium | 0.03 | 0.02 | 0.28 |
| Fusicatenibacter | 0.00 | 0.02 | 0.85 |
| Anaerotruncus | 0.01 | 0.02 | 0.75 |
| Howardella | 0.03 | 0.03 | 0.27 |
| Gordonibacter | 0.02 | 0.03 | 0.59 |
| Coprobacter | 0.00 | 0.02 | 0.89 |
| Eubacteriumventriosumgroup | 0.00 | 0.02 | 0.98 |
| Allisonella | 0.05 | 0.04 | 0.26 |
| Bifidobacterium | 0.00 | 0.01 | 0.78 |
| Hungatella | 0.00 | 0.05 | 0.99 |
| Terrisporobacter | 0.00 | 0.03 | 0.98 |
| Ruminococcusgnavusgroup | 0.04 | 0.03 | 0.31 |
| Eubacteriumcoprostanoligenesgroup | -0.01 | 0.03 | 0.67 |
| Ruminiclostridium6 | 0.02 | 0.01 | 0.21 |
| Coprococcus1 | -0.01 | 0.01 | 0.36 |
| Oscillibacter | 0.00 | 0.02 | 0.85 |
| Parasutterella | -0.01 | 0.01 | 0.39 |
| Lachnoclostridium | 0.00 | 0.02 | 0.89 |
| Holdemania | 0.01 | 0.01 | 0.67 |
| Eubacteriumhalliigroup | 0.00 | 0.01 | 0.79 |
| Eubacteriumrectalegroup | 0.01 | 0.02 | 0.45 |
| Veillonella | 0.05 | 0.04 | 0.17 |
| Actinomyces | 0.00 | 0.02 | 0.84 |
| LachnospiraceaeUCG004 | 0.00 | 0.03 | 0.96 |
| Eubacteriumoxidoreducensgroup | 0.00 | 0.02 | 0.99 |
| Ruminococcus1 | 0.01 | 0.02 | 0.73 |
| Prevotella9 | 0.02 | 0.01 | 0.09 |

MR, Mendelian randomization; CRF, chronic renal failure; SE, standard error.

**Supplementary Tables 4** The heterogeneity of gut microbiota instrumental variables.

| **Exposure(Bacterial taxa)** | **method** | **Cochran's Q** | **Q_df** | **Q_pval** |
| --- | --- | --- | --- | --- |
| Ruminiclostridium9 | IVW | 15.25 | 13 | 0.29 |
| Lactobacillus | IVW | 5.79 | 9 | 0.76 |
| Streptococcus | IVW | 22.89 | 16 | 0.12 |
| Ruminiclostridium5 | IVW | 7.92 | 13 | 0.85 |
| Butyricicoccus | IVW | 9.15 | 8 | 0.33 |
| Victivallis | IVW | 5.96 | 10 | 0.82 |
| ErysipelotrichaceaeUCG003 | IVW | 28.79 | 16 | 0.03 |
| Haemophilus | IVW | 22.59 | 12 | 0.03 |
| Blautia | IVW | 10.57 | 12 | 0.57 |
| Oxalobacter | IVW | 13.11 | 11 | 0.29 |
| Anaerofilum | IVW | 3.33 | 11 | 0.99 |
| LachnospiraceaeNK4A136group | IVW | 17.62 | 15 | 0.28 |
| Eisenbergiella | IVW | 8.94 | 11 | 0.63 |
| Prevotella7 | IVW | 19.87 | 11 | 0.05 |
| Eggerthella | IVW | 8.64 | 10 | 0.57 |
| FamilyXIIIUCG001 | IVW | 18.89 | 9 | 0.03 |
| RikenellaceaeRC9gutgroup | IVW | 8.91 | 11 | 0.63 |
| Paraprevotella | IVW | 5.03 | 12 | 0.96 |
| Parabacteroides | IVW | 9.69 | 8 | 0.29 |
| Ruminococcustorquesgroup | IVW | 22.53 | 12 | 0.03 |
| Alistipes | IVW | 20.43 | 14 | 0.12 |
| Escherichia.Shigella | IVW | 8.73 | 14 | 0.85 |
| Desulfovibrio | IVW | 8.82 | 9 | 0.45 |
| Adlercreutzia | IVW | 8.19 | 11 | 0.70 |
| LachnospiraceaeFCS020group | IVW | 11.10 | 14 | 0.68 |
| Flavonifractor | IVW | 10.35 | 7 | 0.17 |
| Sellimonas | IVW | 5.59 | 10 | 0.85 |
| Romboutsia | IVW | 18.61 | 13 | 0.14 |
| Marvinbryantia | IVW | 5.94 | 9 | 0.75 |
| Collinsella | IVW | 15.15 | 10 | 0.13 |
| Faecalibacterium | IVW | 13.17 | 12 | 0.36 |
| Oscillospira | IVW | 5.51 | 8 | 0.70 |
| Roseburia | IVW | 11.51 | 14 | 0.65 |
| Intestinibacter | IVW | 13.02 | 14 | 0.52 |
| Butyrivibrio | IVW | 11.54 | 15 | 0.71 |
| Turicibacter | IVW | 7.59 | 11 | 0.75 |
| DefluviitaleaceaeUCG011 | IVW | 17.36 | 9 | 0.04 |
| LachnospiraceaeUCG001 | IVW | 12.84 | 15 | 0.61 |
| Eubacteriumxylanophilumgroup | IVW | 12.05 | 10 | 0.28 |
| Akkermansia | IVW | 6.47 | 11 | 0.84 |
| LachnospiraceaeNC2004group | IVW | 7.86 | 9 | 0.55 |
| RuminococcaceaeUCG005 | IVW | 29.32 | 16 | 0.02 |
| Bilophila | IVW | 17.08 | 15 | 0.31 |
| Slackia | IVW | 3.15 | 8 | 0.92 |
| Eubacteriumbrachygroup | IVW | 13.11 | 10 | 0.22 |
| FamilyXIIIAD3011group | IVW | 17.82 | 14 | 0.22 |
| Lactococcus | IVW | 9.41 | 10 | 0.49 |
| ChristensenellaceaeR.7group | IVW | 7.34 | 9 | 0.60 |
| Eubacteriumnodatumgroup | IVW | 9.11 | 10 | 0.52 |
| Clostridiumsensustricto1 | IVW | 3.32 | 8 | 0.91 |
| RuminococcaceaeUCG003 | IVW | 10.72 | 13 | 0.63 |
| Subdoligranulum | IVW | 14.18 | 12 | 0.29 |
| Lachnospira | IVW | 3.81 | 6 | 0.70 |
| Methanobrevibacter | IVW | 12.29 | 7 | 0.09 |
| RuminococcaceaeUCG013 | IVW | 14.26 | 13 | 0.36 |
| Odoribacter | IVW | 6.79 | 7 | 0.45 |
| Tyzzerella3 | IVW | 12.14 | 13 | 0.52 |
| Anaerostipes | IVW | 10.96 | 14 | 0.69 |
| Enterorhabdus | IVW | 4.77 | 6 | 0.57 |
| Erysipelatoclostridium | IVW | 15.11 | 15 | 0.44 |
| RuminococcaceaeUCG014 | IVW | 14.82 | 17 | 0.61 |
| LachnospiraceaeUCG008 | IVW | 7.29 | 11 | 0.77 |
| Bacteroides | IVW | 4.75 | 11 | 0.94 |
| Dorea | IVW | 9.10 | 11 | 0.61 |
| Eubacteriumeligensgroup | IVW | 4.43 | 9 | 0.88 |
| Clostridiuminnocuumgroup | IVW | 13.95 | 10 | 0.18 |
| Holdemanella | IVW | 24.02 | 13 | 0.03 |
| RuminococcaceaeUCG010 | IVW | 3.85 | 7 | 0.80 |
| Butyricimonas | IVW | 13.92 | 15 | 0.53 |
| Dialister | IVW | 13.54 | 11 | 0.26 |
| RuminococcaceaeUCG002 | IVW | 29.45 | 23 | 0.17 |
| LachnospiraceaeND3007group | IVW | 3.96 | 3 | 0.27 |
| Sutterella | IVW | 12.07 | 11 | 0.36 |
| Eubacteriumfissicatenagroup | IVW | 7.56 | 8 | 0.48 |
| Peptococcus | IVW | 9.30 | 15 | 0.86 |
| Coprococcus2 | IVW | 3.72 | 8 | 0.88 |
| RuminococcaceaeNK4A214group | IVW | 12.40 | 15 | 0.65 |
| RuminococcaceaeUCG011 | IVW | 4.28 | 7 | 0.75 |
| LachnospiraceaeUCG010 | IVW | 11.16 | 11 | 0.43 |
| Alloprevotella | IVW | 3.84 | 6 | 0.70 |
| Eubacteriumruminantiumgroup | IVW | 20.17 | 18 | 0.32 |
| Barnesiella | IVW | 10.70 | 15 | 0.77 |
| Ruminococcusgauvreauiigroup | IVW | 10.31 | 12 | 0.59 |
| Coprococcus3 | IVW | 4.09 | 8 | 0.85 |
| Catenibacterium | IVW | 4.69 | 4 | 0.32 |
| Ruminococcus2 | IVW | 12.47 | 14 | 0.57 |
| Intestinimonas | IVW | 33.32 | 19 | 0.02 |
| CandidatusSoleaferrea | IVW | 8.48 | 12 | 0.75 |
| RuminococcaceaeUCG004 | IVW | 10.97 | 11 | 0.45 |
| Senegalimassilia | IVW | 6.63 | 4 | 0.16 |
| Olsenella | IVW | 18.16 | 10 | 0.05 |
| RuminococcaceaeUCG009 | IVW | 10.96 | 13 | 0.61 |
| Phascolarctobacterium | IVW | 3.57 | 11 | 0.98 |
| Fusicatenibacter | IVW | 10.15 | 17 | 0.90 |
| Anaerotruncus | IVW | 11.54 | 14 | 0.64 |
| Howardella | IVW | 8.21 | 10 | 0.61 |
| Gordonibacter | IVW | 22.82 | 13 | 0.04 |
| Coprobacter | IVW | 10.78 | 13 | 0.63 |
| Eubacteriumventriosumgroup | IVW | 12.22 | 14 | 0.59 |
| Allisonella | IVW | 3.90 | 8 | 0.87 |
| Bifidobacterium | IVW | 12.54 | 14 | 0.56 |
| Hungatella | IVW | 2.62 | 4 | 0.62 |
| Terrisporobacter | IVW | 6.93 | 4 | 0.14 |
| Ruminococcusgnavusgroup | IVW | 20.09 | 11 | 0.04 |
| Eubacteriumcoprostanoligenesgroup | IVW | 22.32 | 14 | 0.07 |
| Ruminiclostridium6 | IVW | 14.35 | 15 | 0.50 |
| Coprococcus1 | IVW | 9.56 | 12 | 0.65 |
| Oscillibacter | IVW | 24.34 | 16 | 0.08 |
| Parasutterella | IVW | 16.10 | 16 | 0.45 |
| Lachnoclostridium | IVW | 8.35 | 12 | 0.76 |
| Holdemania | IVW | 6.43 | 17 | 0.99 |
| Eubacteriumhalliigroup | IVW | 10.42 | 15 | 0.79 |
| Eubacteriumrectalegroup | IVW | 11.35 | 11 | 0.41 |
| Veillonella | IVW | 13.30 | 10 | 0.21 |
| Actinomyces | IVW | 7.82 | 7 | 0.35 |
| LachnospiraceaeUCG004 | IVW | 23.01 | 12 | 0.03 |
| Eubacteriumoxidoreducensgroup | IVW | 1.49 | 5 | 0.91 |
| Ruminococcus1 | IVW | 14.16 | 12 | 0.29 |
| Prevotella9 | IVW | 17.21 | 17 | 0.44 |

df, degree of freedom
